# Evaluation of the Force System Acting Within an Orthodontic Appliance

**DOI:** 10.3390/bioengineering13091069

**Published:** 2026-09-14

**Authors:** Amelia Smaranda Roșianu, Dragoș Laurențiu Popa, Gabriel Buciu, Stelian-Mihai-Sever Petrescu

**Affiliations:** 1 Department of Oral Rehabilitation, University of Medicine and Pharmacy of Craiova, 200349 Craiova, Romania; amelia.popescu@umfcv.ro; 2 Department of Automotive, Transportation and Industrial Engineering, Faculty of Mechanics, University of Craiova, 200478 Craiova, Romania; 3 Department of General Nursing, Faculty of Nursing-Târgu Jiu, Titu Maiorescu University, 210102 Târgu Jiu, Romania; 4 Department of Orthodontics, University of Medicine and Pharmacy of Craiova, 200349 Craiova, Romania; mihaipetrescu2702@gmail.com

**Keywords:** pre-prosthetic orthodontics, three-dimensional modeling, CBCT, orthodontic biomechanics, elastic forces, bracket, nickel–titanium archwire

## Abstract

Background: Implant-prosthetic rehabilitation of single-tooth edentulism often requires orthodontic reshaping of the potential implant-prosthetic space because its dimensions change due to the migration of adjacent teeth. In this context, it is very important to understand the force system generated by orthodontic arches in order to control tooth movement and to achieve, at the end of orthodontic treatment, the correct dimensions of the potential implant-prosthetic space for dental implant placement. Materials and Methods: The study was based on CBCT images acquired from a 25-year-old female patient with maxillary and mandibular lateral edentulism. The images were processed using InVesalius and Geomagic to obtain a patient-specific three-dimensional reconstruction of the dento-maxillary structures. Orthodontic brackets, adhesive components, and round-section nickel–titanium archwires were subsequently modeled and positioned within a common three-dimensional coordinate system. The reconstructed geometry was used to extract local geometric parameters of the archwire. These parameters were subsequently introduced into a local equivalent Euler–Bernoulli beam formulation to estimate elastic force components and transverse reactions associated with the bracket-related points. Results: The study revealed an uneven distribution of forces along the orthodontic arches. For the upper arch, the elastic force values ranged from 0.044 to 0.383 N, and for the lower arch, from 0.050 to 0.308 N. The calculated reactions at the bracket components also varied depending on the orthodontic archwire geometry and the position of the support points. Conclusions: Patient-specific three-dimensional reconstruction combined with a local analytical beam approximation provides a framework for estimating geometry-dependent elastic force components within an orthodontic appliance. The calculated values should be interpreted as equivalent local force estimates derived from the reconstructed geometry and not as a complete three-dimensional force–moment system.

## 1. Introduction

Implant-prosthetic rehabilitation of single-tooth edentulism is a functionally and aesthetically predictable solution, but its success depends essentially on a well-designed interdisciplinary (prosthetic and orthodontic) treatment plan. One of the determining factors for correct implant placement is the availability of adequate dimensions of the potential implant-prosthetic space—mesiodistally, vertically, and buccal-orally—so that the final restoration complies with the biomechanical, aesthetic, and biological principles of implant-supported prosthetic restoration [1,2].

In many clinical situations, early tooth loss is followed by migration of adjacent teeth, tooth tilting, changes in occlusal contacts, and a reduction in the space required for implant placement. In these cases, orthodontic treatment plays a fundamental role by realigning dental axes, converging or parallelizing the roots, and restoring the size of the edentulous space to a position favorable for prosthetic restoration [2,3]. Thus, orthodontics serves not only to “create space” but also to optimize the three-dimensional conditions for the implant, contributing to the correct positioning of the future prosthetic abutment and the final crown.

The importance of this stage is particularly heightened in the anterior regions, where aesthetic demands are high, and any discrepancy in position or volume can compromise the final result. The literature indicates that implants placed without adequate orthodontic preparation may be associated with difficulties in prosthetic emergence, gingival margin asymmetries, or compromises in the aesthetic contour of the restoration [1,2]. Conversely, through proper treatment sequencing, orthodontics facilitates implant placement in an area with sufficient space and favorable root relationships, reducing the risk of prosthetic and surgical complications.

Furthermore, in growing patients, the timing of implant placement must be carefully considered, as implants behave like ankylosed structures and do not follow the physiological growth of the jawbones. For this reason, in certain situations, orthodontic treatment also serves to maintain or reopen the space until skeletal maturity is reached, at which point implantation can be performed under conditions of long-term stability [4]. This stage is essential for preventing vertical discrepancies and for maintaining dentoalveolar harmony over time.

Consequently, the role of orthodontics in creating space for implants goes beyond simple dental alignment, integrating biological, functional, and aesthetic principles into an interdisciplinary treatment plan [4,5]. However, to successfully achieve these objectives, it is necessary to understand and precisely adhere to the system of orthodontic forces capable of moving the teeth to align and reshape the implant-prosthetic space in accordance with bone biology, without causing excessive bone resorption. Only in this way can the primary and secondary stability of the dental implant be guaranteed [6,7].

In this context, the aim of the present study is to evaluate the entire system of forces acting on metal bracket-type components in an orthodontic setup designed to reshape the potential implant-prosthetic space.

## 2. Materials and Methods

### 2.1. CBCT Imaging Data

This study was based on a set of CBCT images acquired at a specialized dental clinic on 25 January 2024, for a 25-year-old female patient presenting with maxillary and mandibular lateral edentulism. This study was approved by Decision No. 156/30 August 2023, of the Ethics Committee and was conducted with the patient’s consent.

As part of the study, a geometric model of the tissues was created based on the set of CBCT (Cone Beam Computed Tomography) images. The data were processed using software that converts CBCT images into primary geometries for the constituent tissues using specific filters based on different shades of gray. The initial geometry, which consisted of a “point cloud,” was processed using techniques and methods specific to reverse engineering. Initially, this “point cloud” was transformed into a multitude of spatial triangular surfaces, which subsequently formed perfectly closed surfaces for the bone components (maxilla and mandible) and for the specific dentition. Using techniques specific to both Reverse Engineering and Direct Engineering, based on “offset” surfaces, models of the periodontal ligaments for each tooth were obtained.

The models were loaded, one by one, into a CAD (Computer-Aided Design) program, where they were aligned based on a common coordinate system and converted into virtual solids. Within the same software, the metal components for the orthodontic wires and bracket-type elements were modeled using techniques specific to Direct Engineering. These models were added to the models of the bone, tooth, and periodontal ligament components. Elastic deformations were measured on the orthodontic archwire models in the virtual environment. Based on specific equations from the field of Strength of Materials, the elastic forces were determined, followed by the reactions; finally, the entire system of forces acting on the bracket-type components was evaluated.

**Figure 1:**
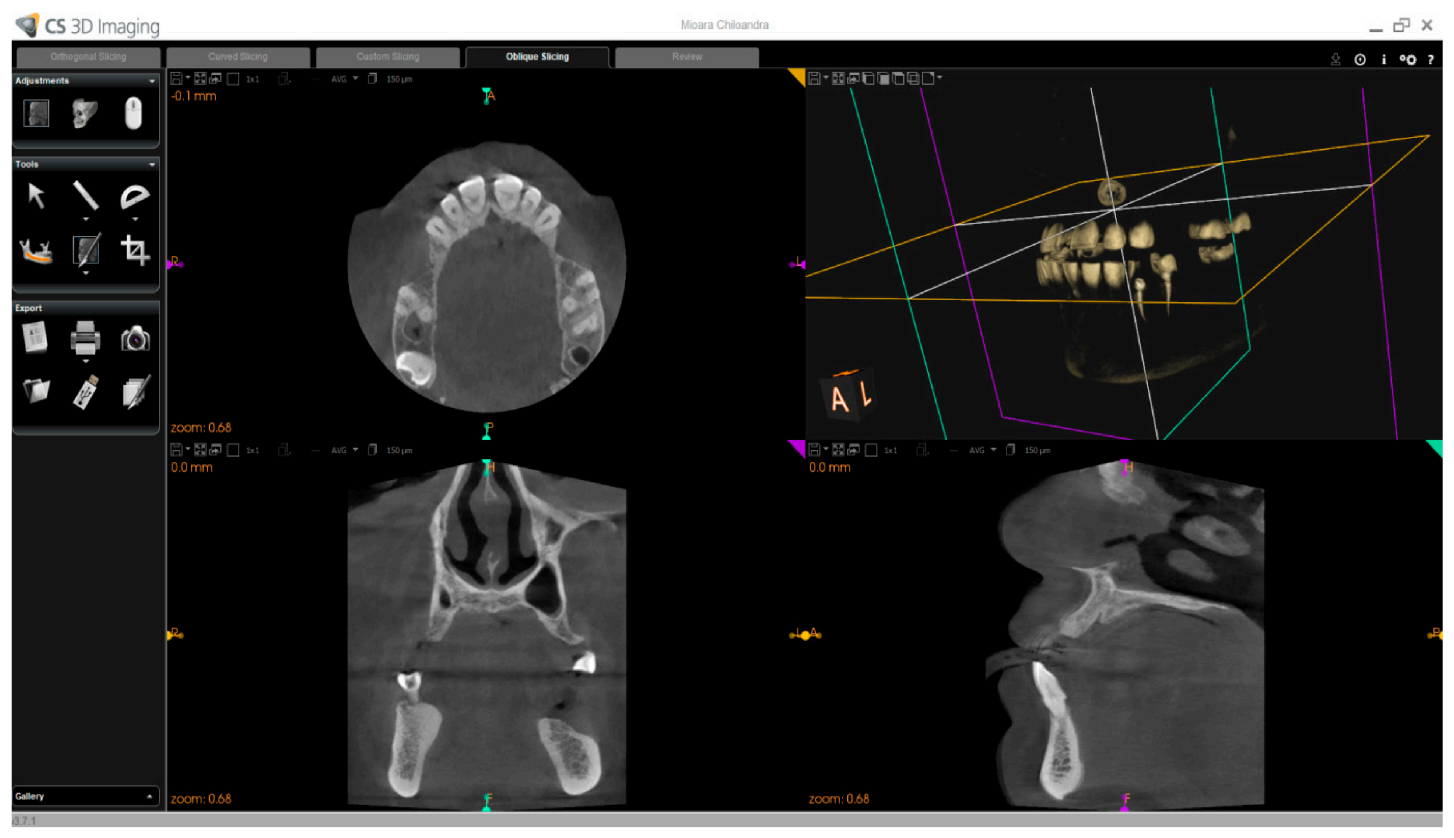
CBCT images of the patient under study.

Figure 1 shows a screenshot from the CS 3D Imaging program (version 3.10.43) for the set of tomographic images containing 658 CBCT images for the patient under study.

### 2.2. Orthodontic Materials

**Figure 2:**
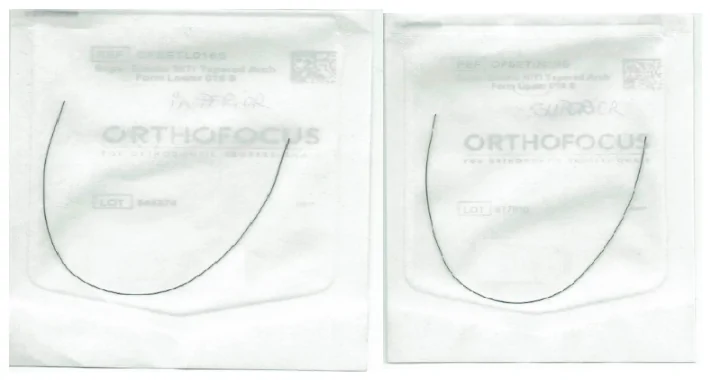
Orthofocus Orthodontic Archwire Type.

For the orthodontic assembly, we analyzed, from a dimensional perspective, Orthofocus-type orthodontic wires for the maxilla and mandible with a circular cross-section and a diameter of 0.012 inches (approx. 0.3 mm), as shown in Figure 2.

**Figure 3:**
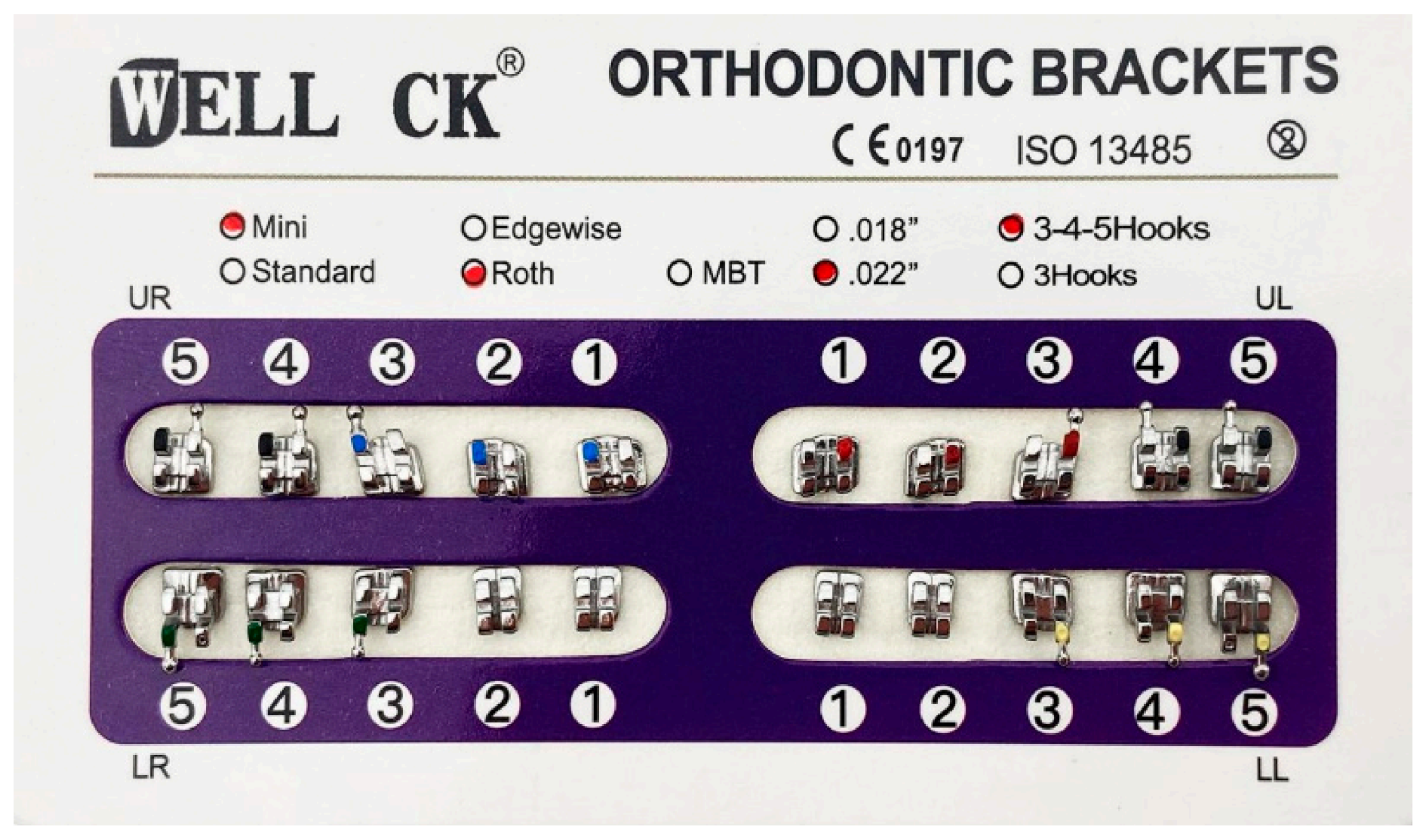
Bracket-type elements.

Similarly, the bracket-type elements shown in Figure 3 were studied.

### 2.3. Software Used

#### 2.3.1. InVesalius

InVesalius 3.1.1 is open-source research software used for the three-dimensional reconstruction of medical images obtained through computed tomography (CT) or magnetic resonance imaging (MRI). The software converts tomographic images into 3D models of the body’s anatomical tissues to better understand internal biological structures. The resulting 3D models are available in standard formats (such as STL, OBJ, or PLY) and can be used for virtual prototyping and 3D printing. The software is used for medical diagnosis, surgical planning, dentistry, research, education, veterinary medicine, and even in engineering for the virtual prototyping of medical devices. It was developed by the Renato Archer Information Technology Center (CTI) in Brazil (CTI, Campinas, Brazil).

#### 2.3.2. Geomagic

Geomagic Wrap 2021.2.0 is a professional software program for reverse engineering, 3D scanning, and dimensional inspection, used in the medical, automotive, and aerospace industries. Geomagic enables the conversion of 3D scans—which initially consist of “point clouds”—into CAD models. The software enables the conversion of 3D scans into CAD models consisting of surfaces or solids, the reconstruction of a digital model from a physical part, the comparison of manufactured parts with the original CAD model, and the “cleaning and repair” of meshes such as STL, OBJ, etc. (3D Systems, Rock Hill, SC, USA).

#### 2.3.3. SolidWorks

SolidWorks 2021 is a professional computer-aided design (CAD) program used to generate 3D models of components, assemblies, and technical documentation specific to mechanical engineering and industrial design. It also allows for virtual testing and simulations for strength analysis (Simulation module), kinematic analysis (Motion module), thermal analysis, vibration analysis, and fluid flow analysis (Flow module), as well as fatigue testing (Fatigue module).

This software uses methods specific to Direct Engineering and may contain specialized modules (Dassault Systèmes, Velizy-Villacoublay, France).

#### 2.3.4. Microsoft Office Software Components

Microsoft Office is a suite of software modules developed by Microsoft, used for working with data, documents, presentations, and diagrams and charts.

The Microsoft Office suite was used to draft documents, organize, manage, and analyze data, as well as to implement formulas and perform repetitive calculations (Microsoft Corporation, Redmond, WA, USA).

#### 2.3.5. Artificial Intelligence (AI) Modules

These were used to explain terms, techniques, concepts, and methods, or to detail the advantages and disadvantages of certain software modules.

### 2.4. Hardware Systems

A Hewlett Packard Z2 workstation with the following technical specifications was used for 3D modeling:

- 13th-generation Intel^®^ Core™ i9 processor;
- 64 GB DDR5-4800 RAM (4 x 16 GB);
- Graphics: NVIDIA RTX™ A4500 (20 GB dedicated GDDR6 memory), memory slots: 4 DIMM, internal storage: 1 TB HP Z Turbo Drive PCIe^®^ NVMe™ TLC SSD;
- Graphics: Intel^®^ UHD Graphics, memory and storage: 64 GB memory/1 TB SSD storage;
- Processor: Intel^®^ Core™ i9-13900K (up to 5.8 GHz, with Intel^®^ Turbo Boost technology);
- 36 MB L3 cache, 24 cores, processor L3 cache: 36 MB, processor family;
- Operating system: Windows 11 Pro.

To generate charts and graphs and for computational data analysis, several desktop computers with the following technical specifications were used:

- Intel Core i3 processor with a clock speed of 3.7 GHz;
- 8 GB of RAM;
- 466 GB hard drive;
- 64-bit Windows 10 operating system.

For similar purposes, a Lenovo laptop was also used with the following technical specifications:

- Intel Core i5 processor with a clock speed of 2.9 GHz;
- 476 GB SSD;
- 930 GB hard drive;
- 16 GB RAM;
- 64-bit Windows 10 operating system.

### 2.5. Methods

#### 2.5.1. Methods Specific to Medical Imaging

Medical imaging methods are investigative techniques that allow for the visualization of the body’s structures, tissues, and functions without (or with minimal) surgical intervention. They are very important for medical diagnosis, treatment guidance, and patient monitoring [8].

The main medical imaging methods are based on radiography (X-ray), computed tomography (CT or CBCT), magnetic resonance imaging (MRI), ultrasound, nuclear medicine, fluoroscopy, or angiography.

#### 2.5.2. Methods of Elasticity Theory

The methods of Elasticity Theory constitute a set of mechanical and mathematical procedures used to analyze the behavior of bodies considered elastic (deformable solids) when subjected to a system of forces or other external actions [9,10,11].

The use of these methods leads to the determination of strains, stresses, and displacements in a deformable solid [12,13,14].

The main methods of the Theory of Elasticity are: the analytical (exact) method, the energy method (energetic methods), the approximate method, the numerical method, and the experimental method [15,16,17].

#### 2.5.3. Methods of Strength of Materials

The methods of strength of materials consist of a set of computational procedures, principles, and assumptions used to analyze the behavior of deformable solid bodies (plates, beams, bars, shafts) when they are subjected to a system of forces and moments [18,19].

By applying these methods, it is possible to determine the deformations, displacements, and stresses in the deformable solid under analysis. Furthermore, the conditions for strength, stability, and stiffness can be established [20,21,22]

These methods include: the section method, the method of static equilibrium, the allowable stress method, the deformation method, the deformation energy method, the superposition of effects method, and (modern) numerical methods [23,24,25].

#### 2.5.4. Reverse Engineering Methods

Reverse engineering methods consist of a set of processes and techniques used to study an existing product, system, or software in order to understand its structure, operation, and design principles without access to the original documentation.

This study will utilize methods specific to mechanical engineering, which may be based on 3D scanning, manual measurements, CAD reconstruction, materials analysis, and physical disassembly. Analytical and functional methods may also be used, such as functional analysis, mathematical modeling, numerical simulation, benchmarking, or AI-based reverse engineering.

#### 2.5.5. Direct Engineering Methods

Direct Engineering Methods (also known as Forward Engineering) refer to the set of techniques and methods used to design and build a system, starting from requirements or design briefs and gradually moving toward actual implementation. In practice, the process begins with an idea, leads to a model, and then to a final product.

Forward engineering is a system development process that begins with requirements and specifications, proceeds to the creation of conceptual and design models, and culminates in implementation, testing, and operation. It is the opposite of reverse engineering, which starts with an existing system.

In this study, these methods were used to model orthodontic wires and bracket-type elements.

### 2.6. Three-Dimensional Model of an Orthodontic System

#### 2.6.1. Three-Dimensional Models of the Bony Components (Maxilla and Mandible)

**Figure 4:**
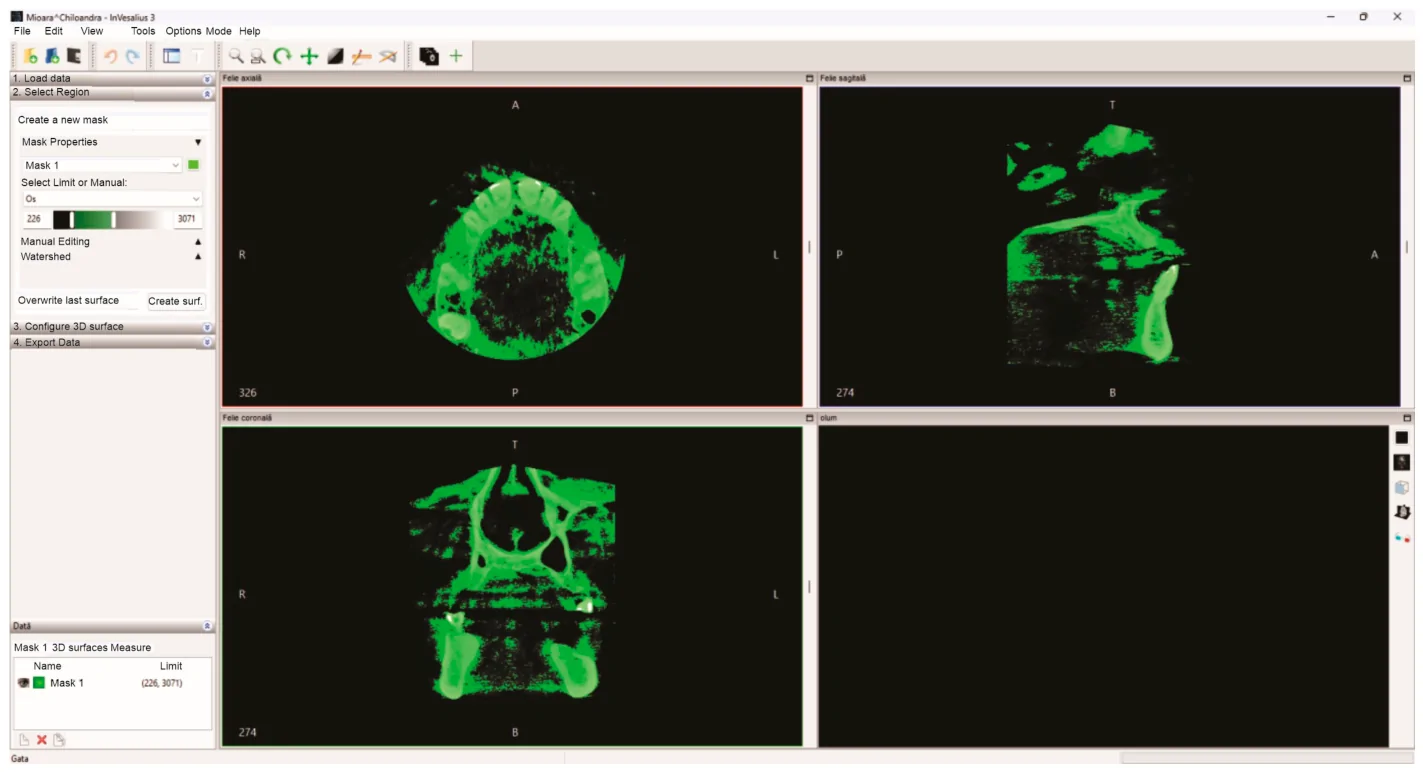
Interface of the InVesalius Software.

The patient’s CBCT scans were imported into the InVesalius program. Figure 4 shows the program interface after importing the set of scans.

**Figure 5:**
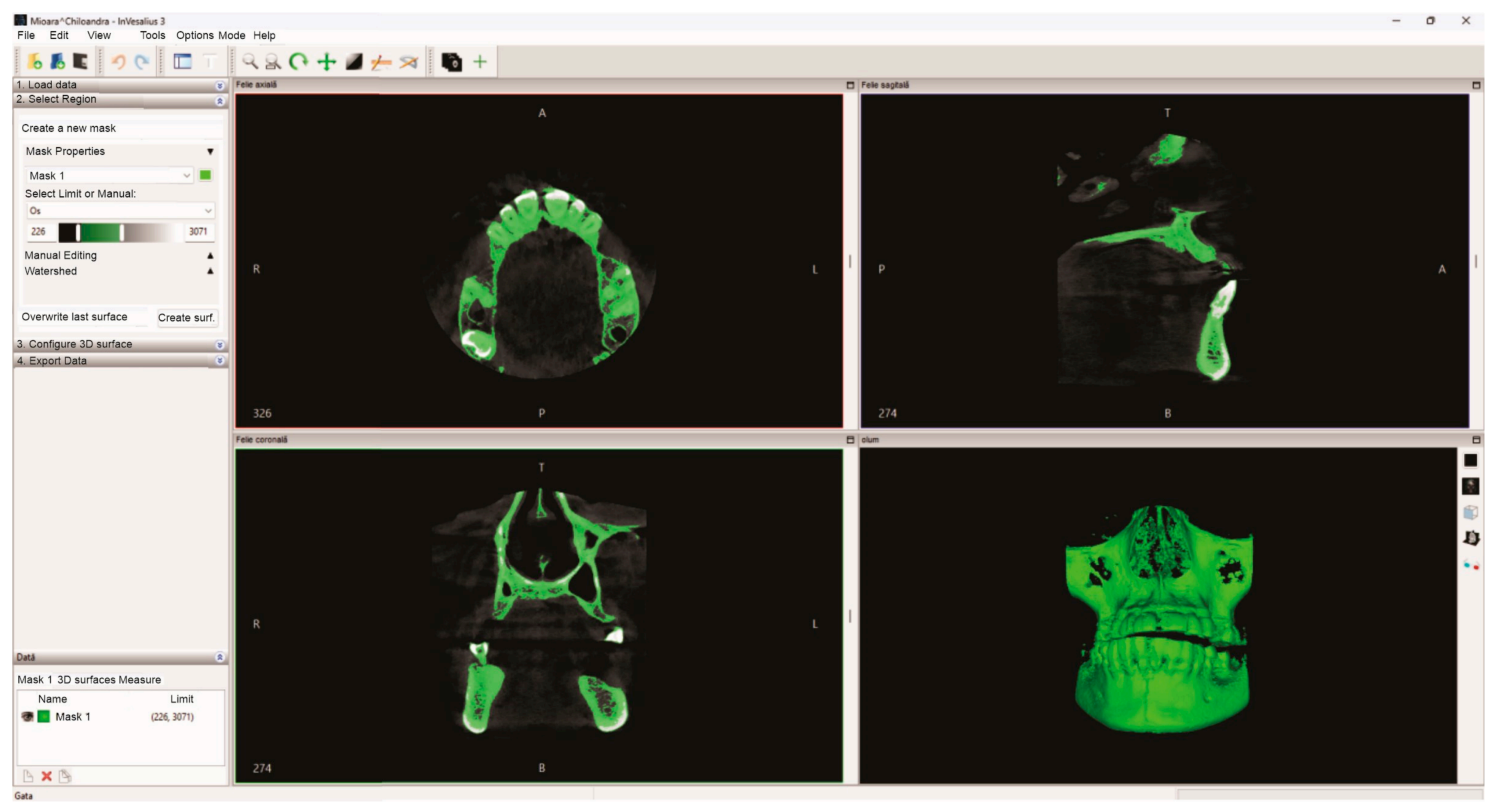
Use of the dental enamel filter.

To obtain a primary “point cloud” geometry, the dental enamel (adult) filter was used, as shown in Figure 5.

**Figure 6:**
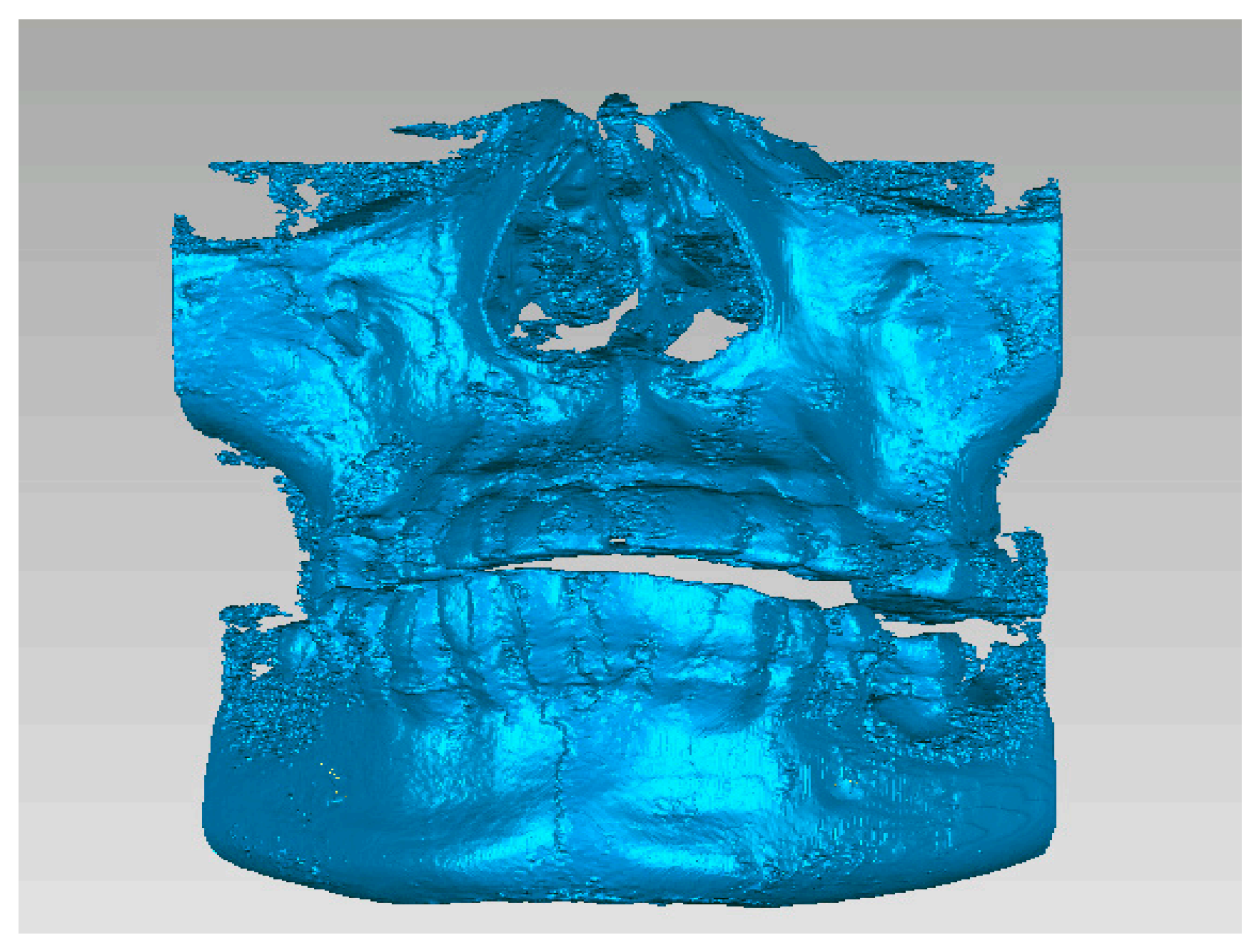
The initial geometry of the model in the Geomagic Software.

Because the shades of gray in enamel-type dental tissues are similar to those of bone components, the model was imported into the Geomagic software for editing and processing. Figure 6 shows the Geomagic interface after importing the model from InVesalius as a “point cloud.” In the first phase, this software automatically transforms the “point cloud” into spatial triangular surfaces. Initially, this model contained 5,093,918 primary triangular surfaces.

**Figure 7:**
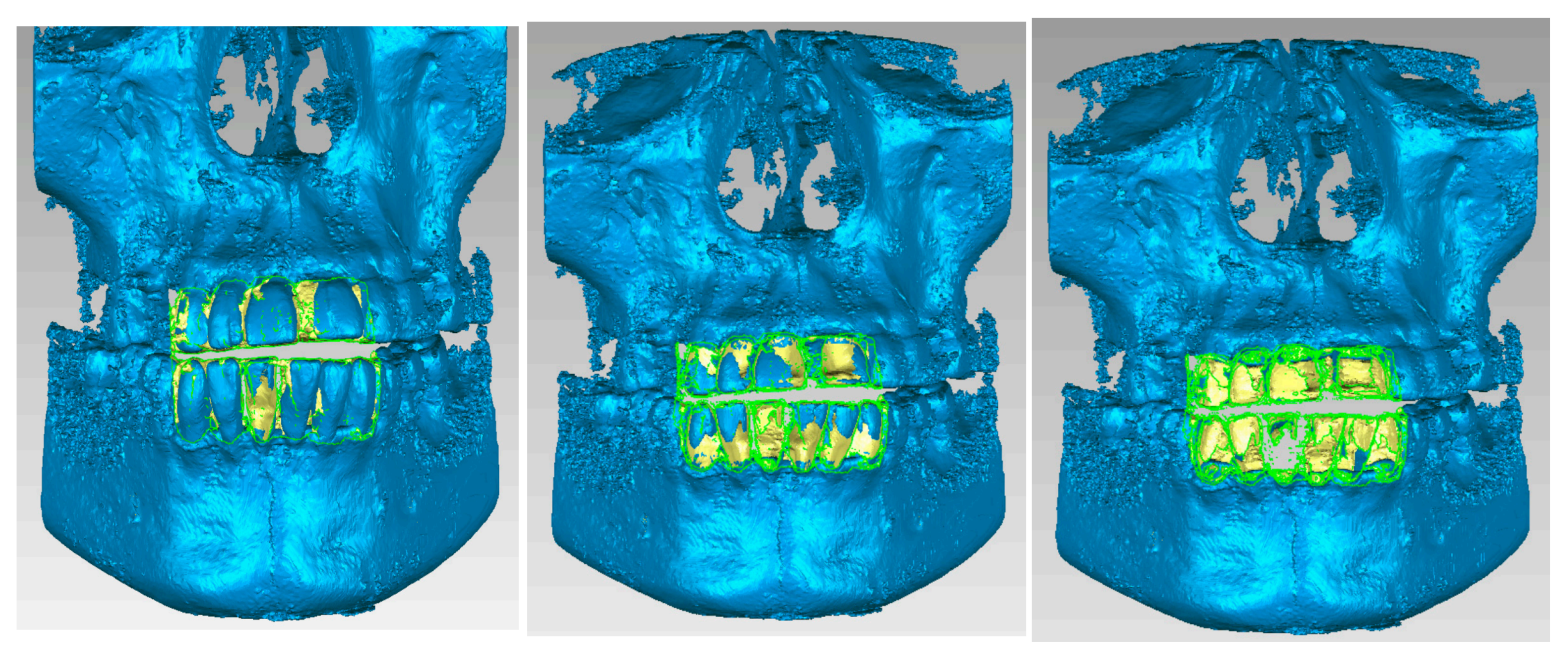

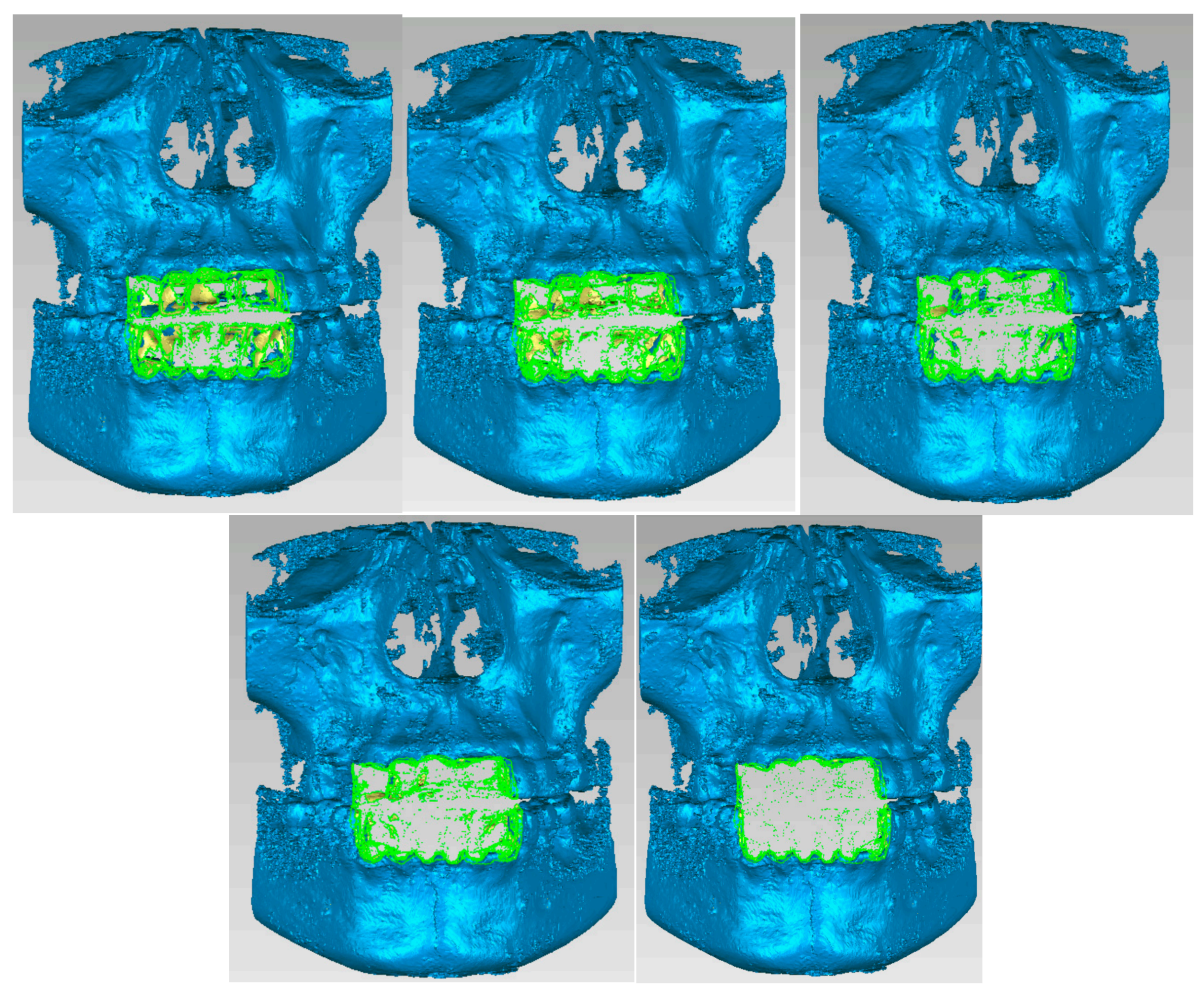
Extraction of front teeth.

This model also contains elements that are part of the dentition due to the similar densities of the tooth enamel. These components were manually removed using specific techniques. Figure 7 shows the steps involved in these removal operations for the anterior region.

**Figure 8:**
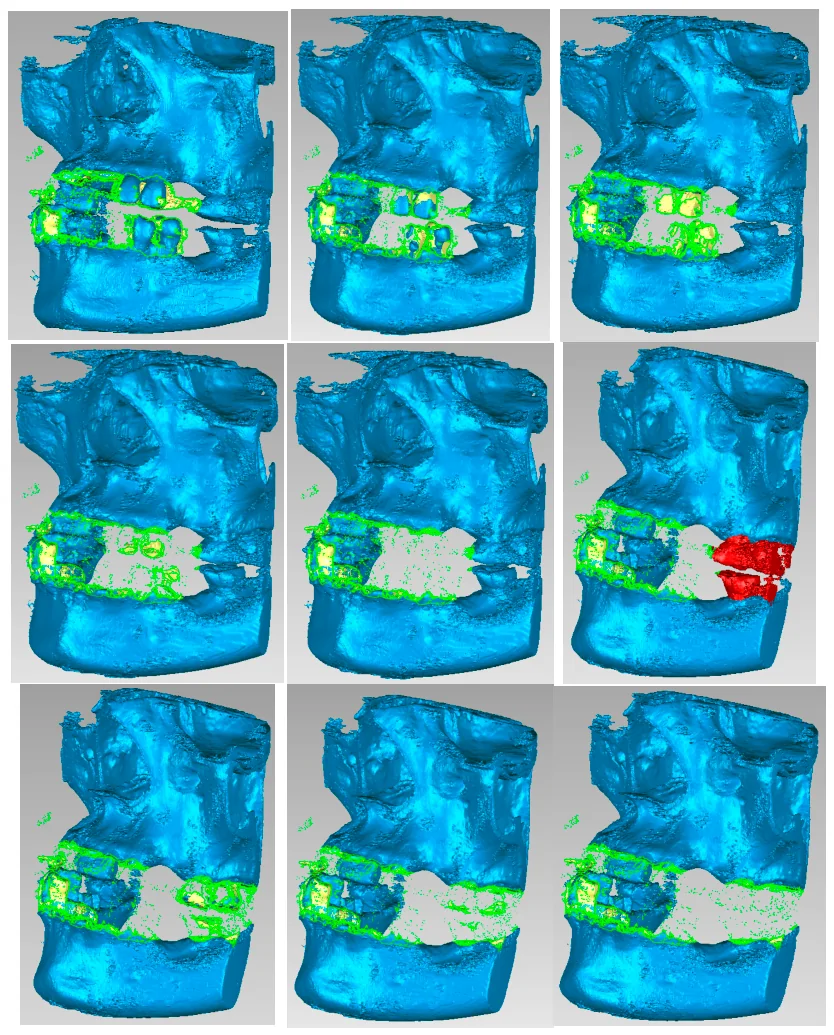
Extraction of the lateral left dentition.

Next, the left lateral dentition was removed; some of these steps are shown in Figure 8.

**Figure 9:**
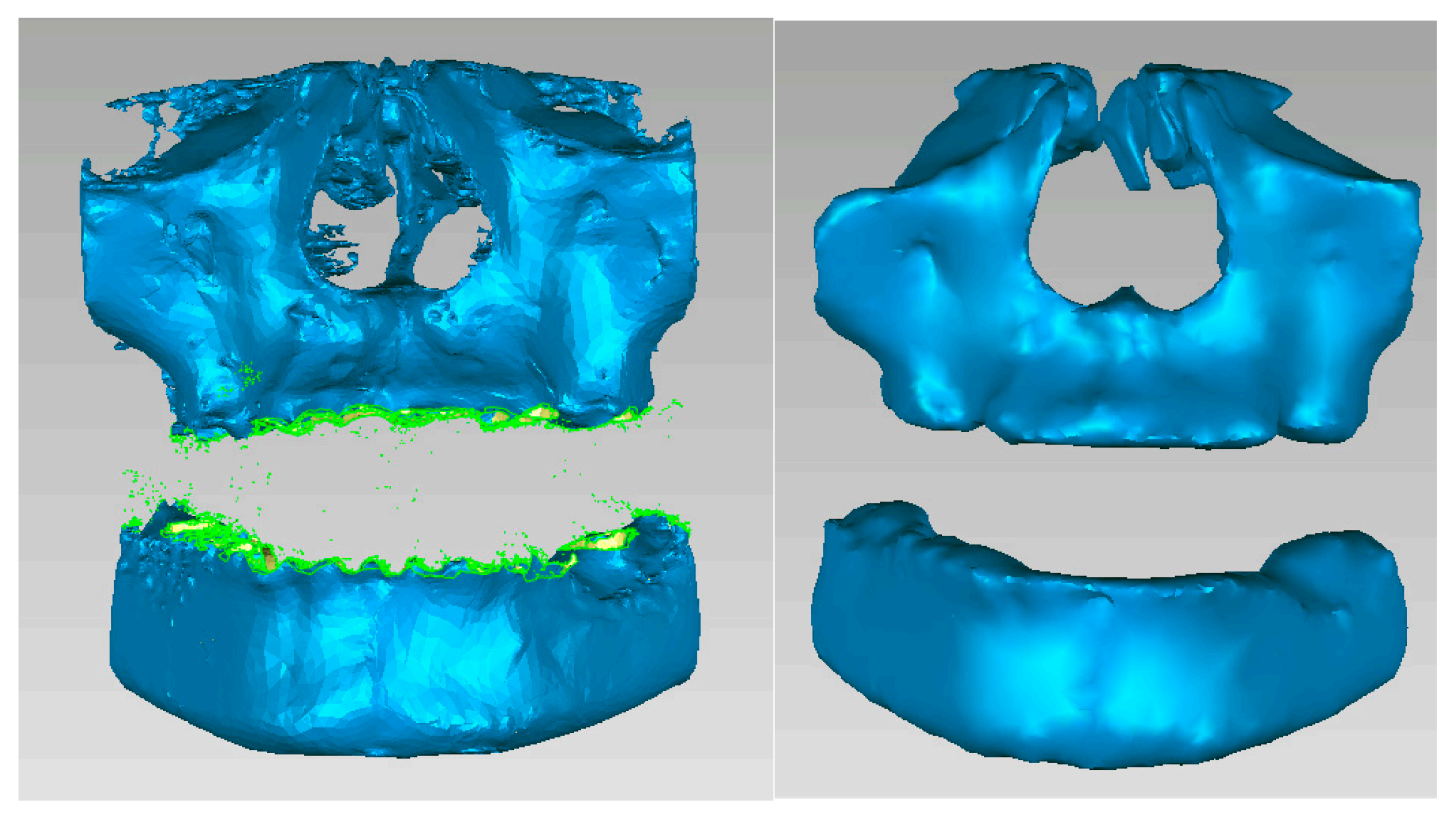
Finalizing the models for the bone components.

Next, various reverse engineering techniques were used to process the model; certain gaps were filled; non-conforming surfaces were identified and removed; finishing techniques were applied; and, finally, the number of elementary triangular surfaces was reduced to 24,198. Figure 9 illustrates some of these operations.

**Figure 10:**
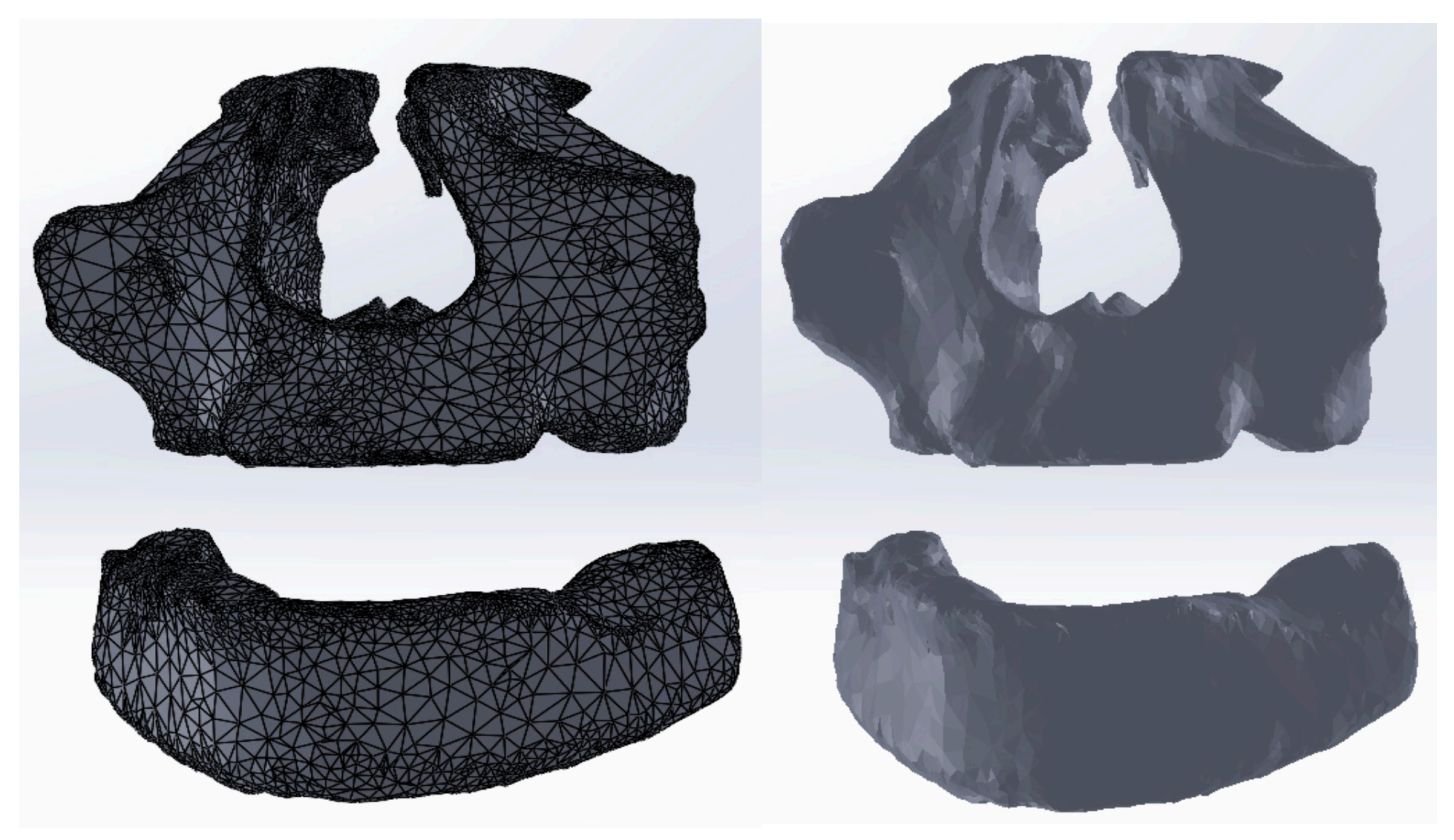
Models imported in SolidWorks.

Finally, the models were imported into SolidWorks, where they were converted into virtual solids, as shown in Figure 10.

#### 2.6.2. Three-Dimensional Models of the Dentition

**Figure 11:**
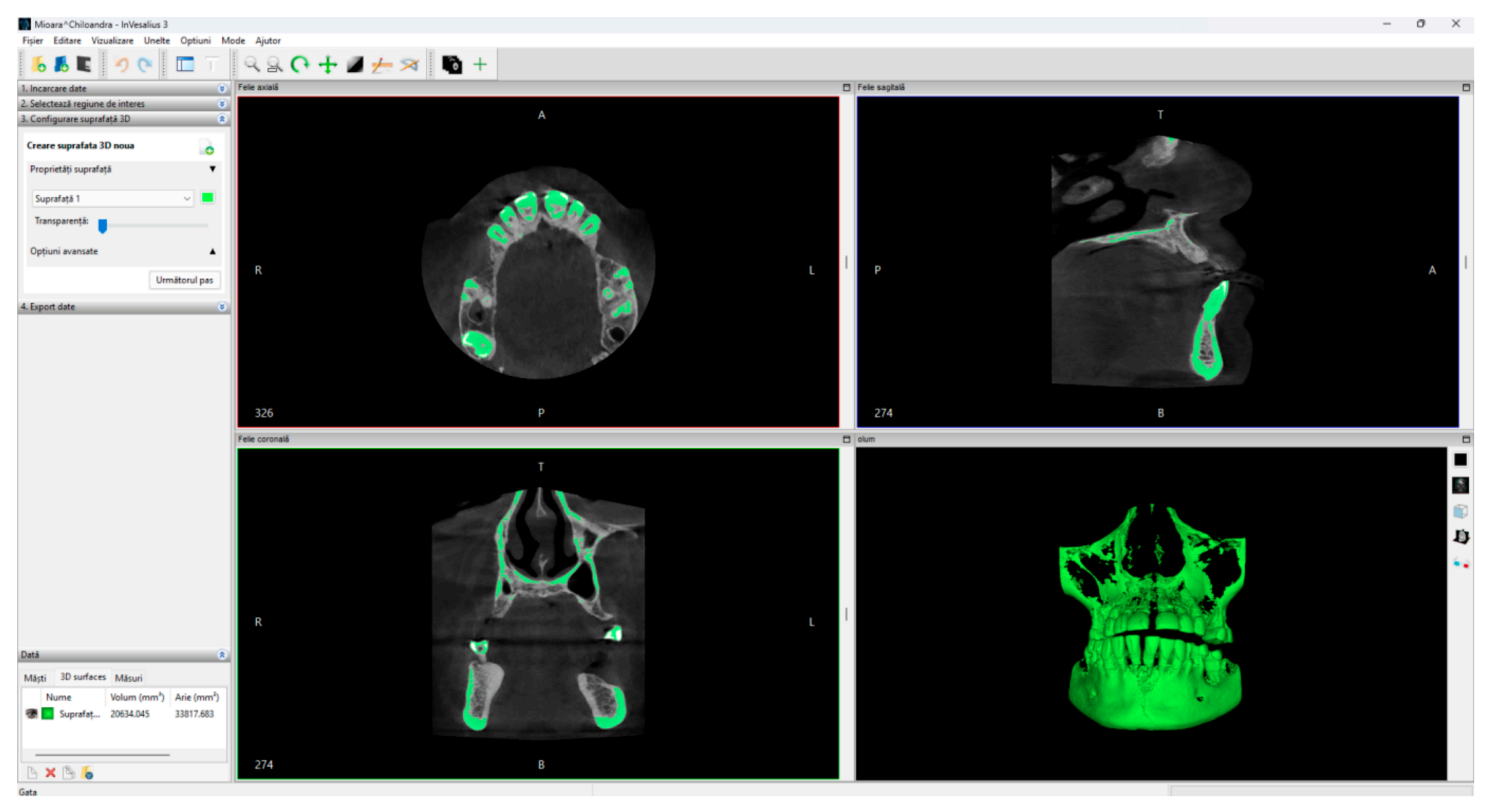
Primary Model of the dentition in InVesalius.

To create the tooth models, the dental enamel filter in the InVesalius program was used, and the initial model shown in Figure 11 was generated.

**Figure 12:**
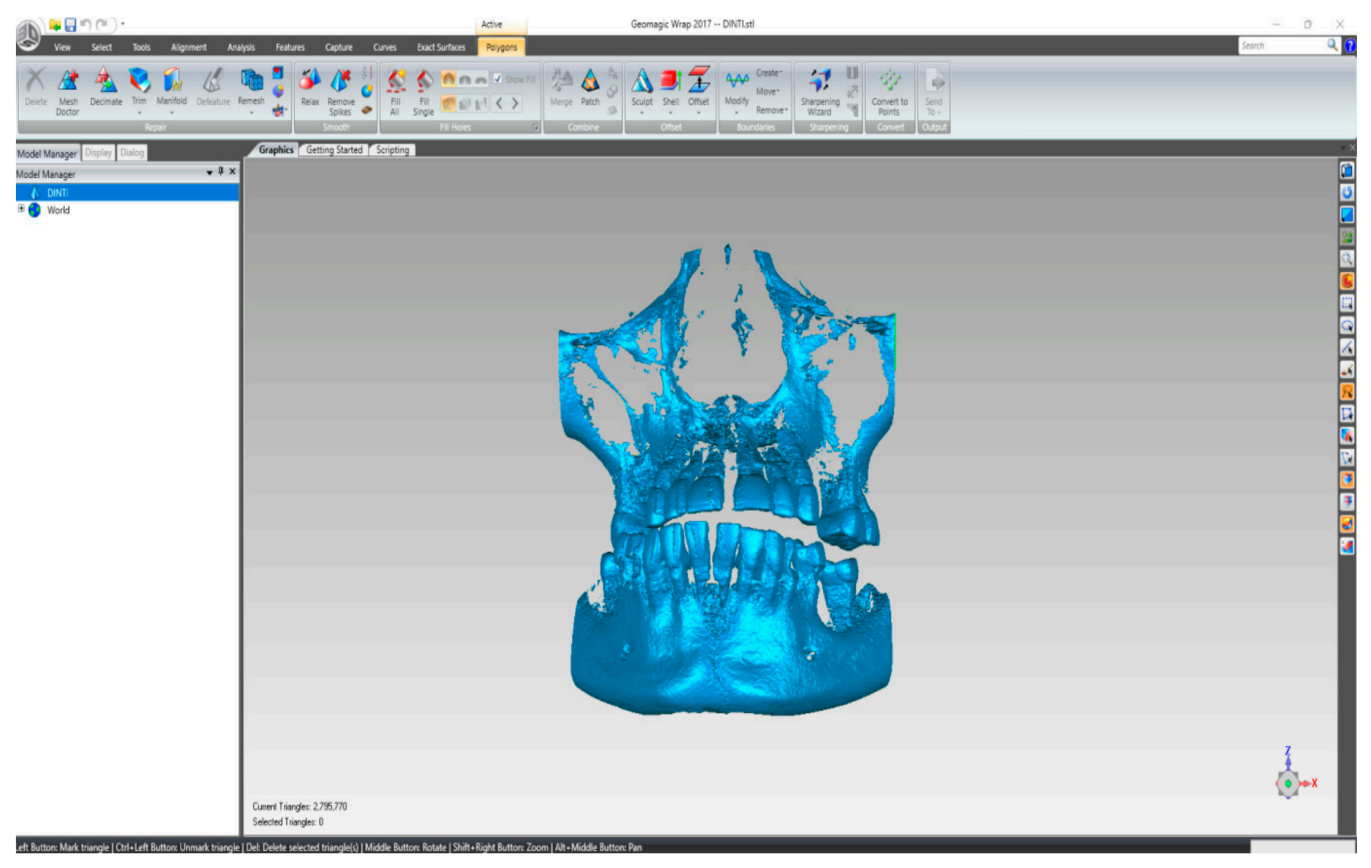
Primary Model of the dentition in Geomagic.

Initially, the model contained 2,795,770 triangular element surfaces in Geomagic, as shown in Figure 12.

**Figure 13:**
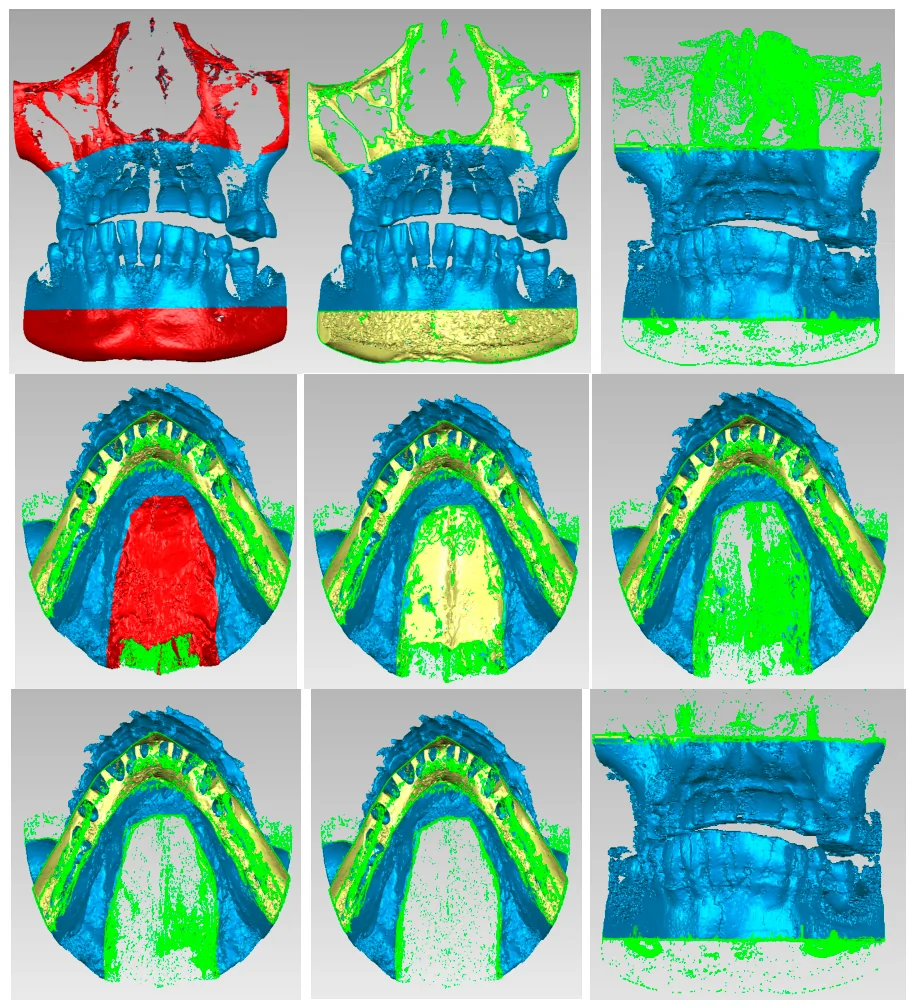
Removal steps in Geomagic.

In the first step, the areas corresponding to the bone components were removed, as shown in Figure 13.

**Figure 14:**
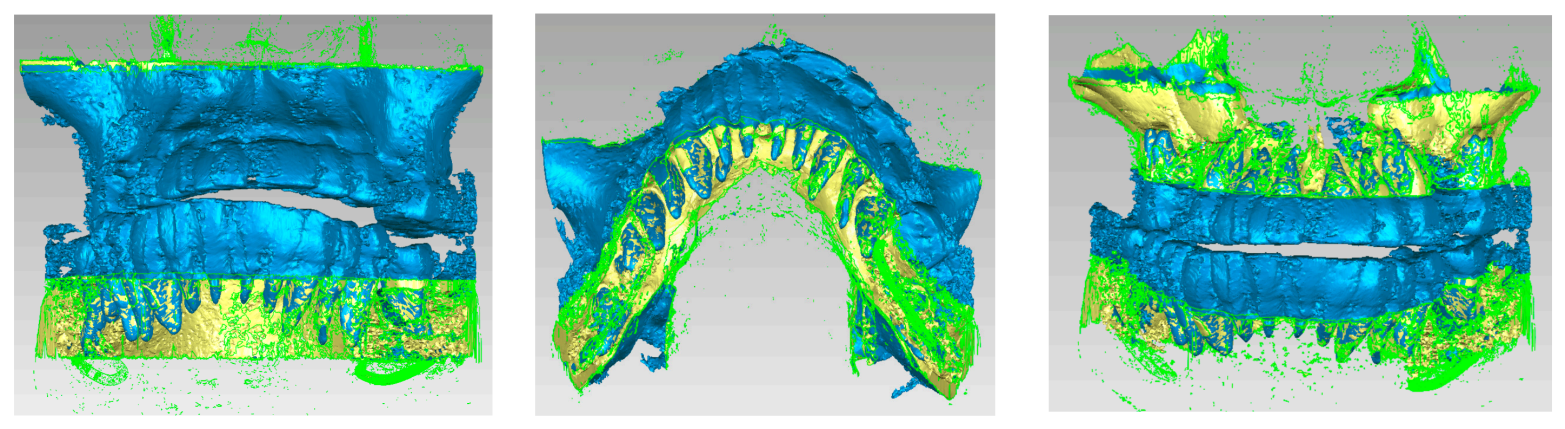

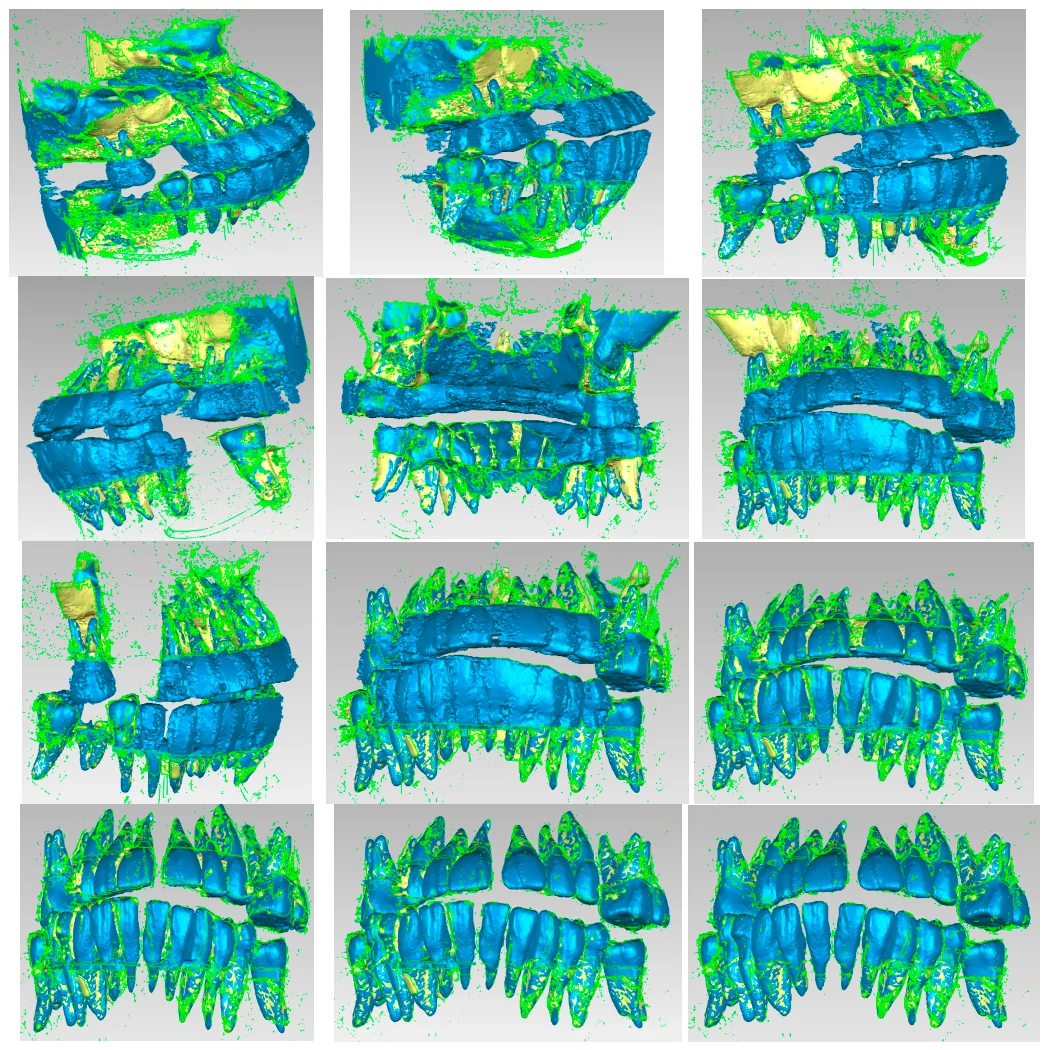
Removal of bone components in Geomagic.

The steps for removing bone components continue, and Figure 14 shows some of them.

**Figure 15:**
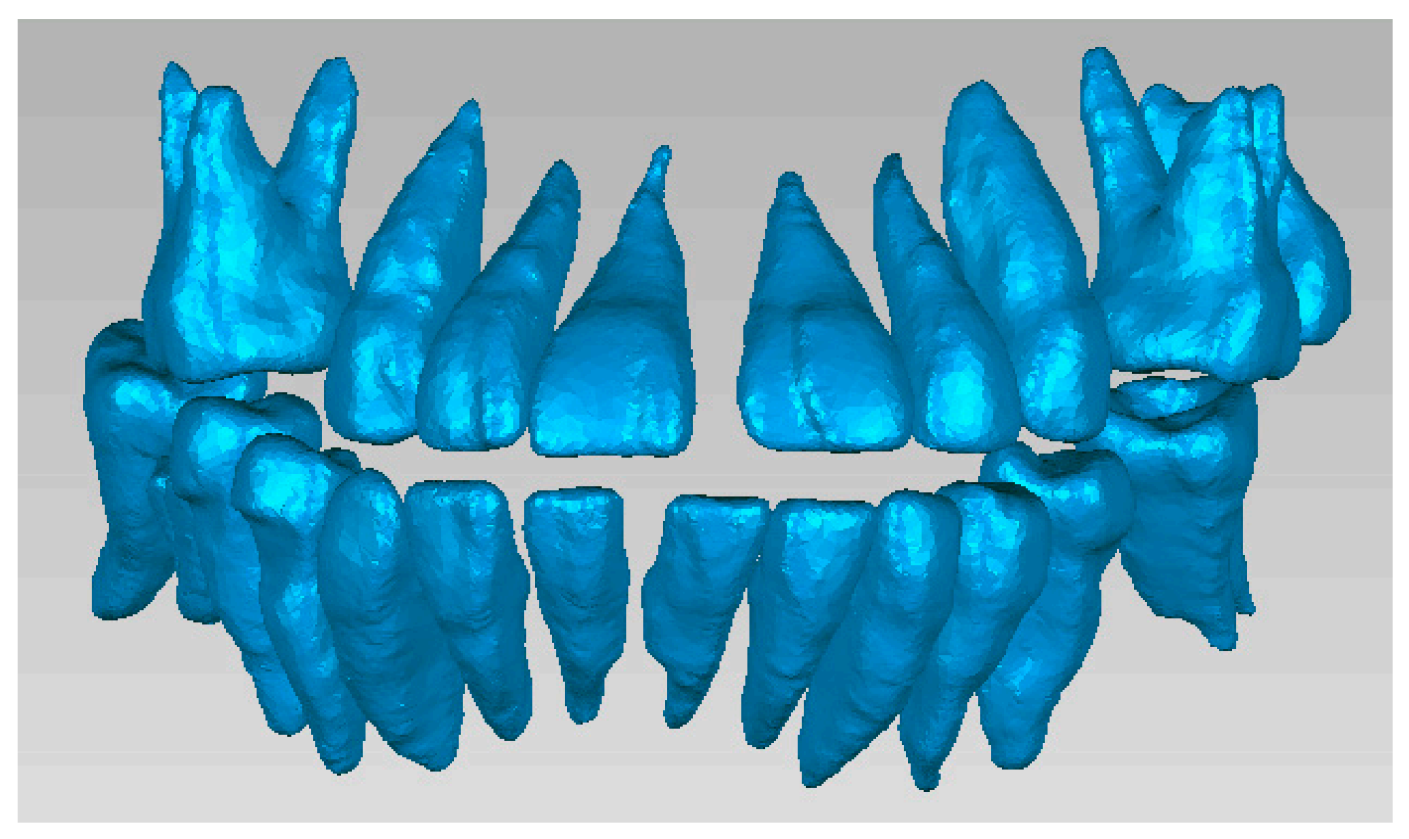
Final Model of the dentition in GeoMagic.

After applying several techniques for finishing, filling gaps, reducing the number of triangular faces, and eliminating non-conforming faces, the model shown in Figure 15 was obtained, which had 100,110 element faces.

**Figure 16:**
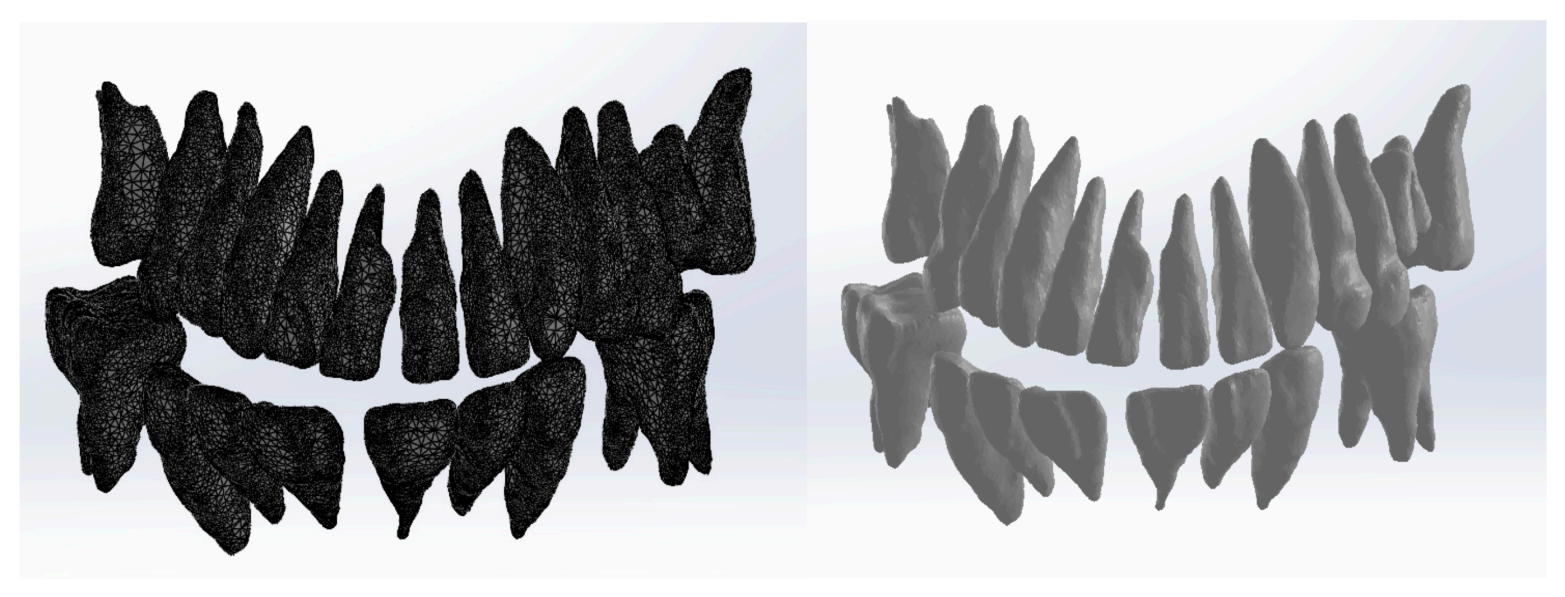
Dental Model in SolidWorks.

Finally, the dental model was imported into SolidWorks, where it was automatically converted into virtual solids, as shown in Figure 16.

#### 2.6.3. Three-Dimensional Models of Dental Ligaments

**Figure 17:**
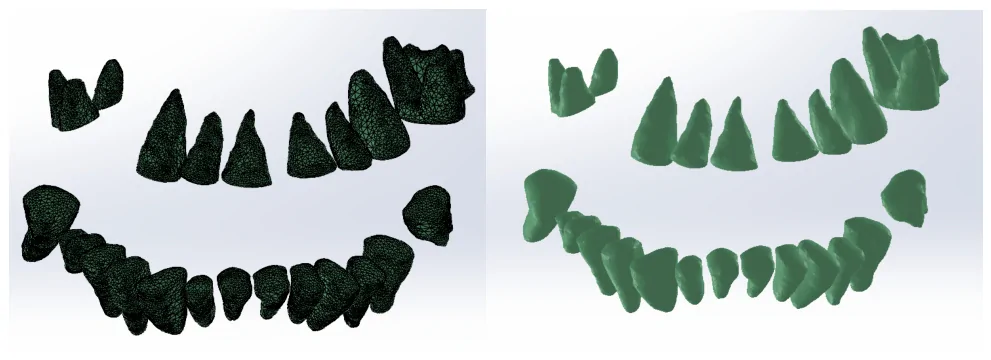
The Initial Model of the Ligaments Imported in SolidWorks.

To generate the models of the dental ligaments, we started with the dentition model and used Offset surfaces applied to the entire model in Geomagic. Subsequently, the surfaces corresponding to the dental crown areas were removed, then filled using Fill techniques to obtain perfectly closed surfaces; finally, the models were imported into SolidWorks, where they were converted into virtual solids, as shown in Figure 17. The cavities corresponding to the contact surfaces with the dentition were subsequently created using CAD techniques.

#### 2.6.4. Three-Dimensional Model of the Dento-Maxillary Apparatus of the Patient Under Study

**Figure 18:**
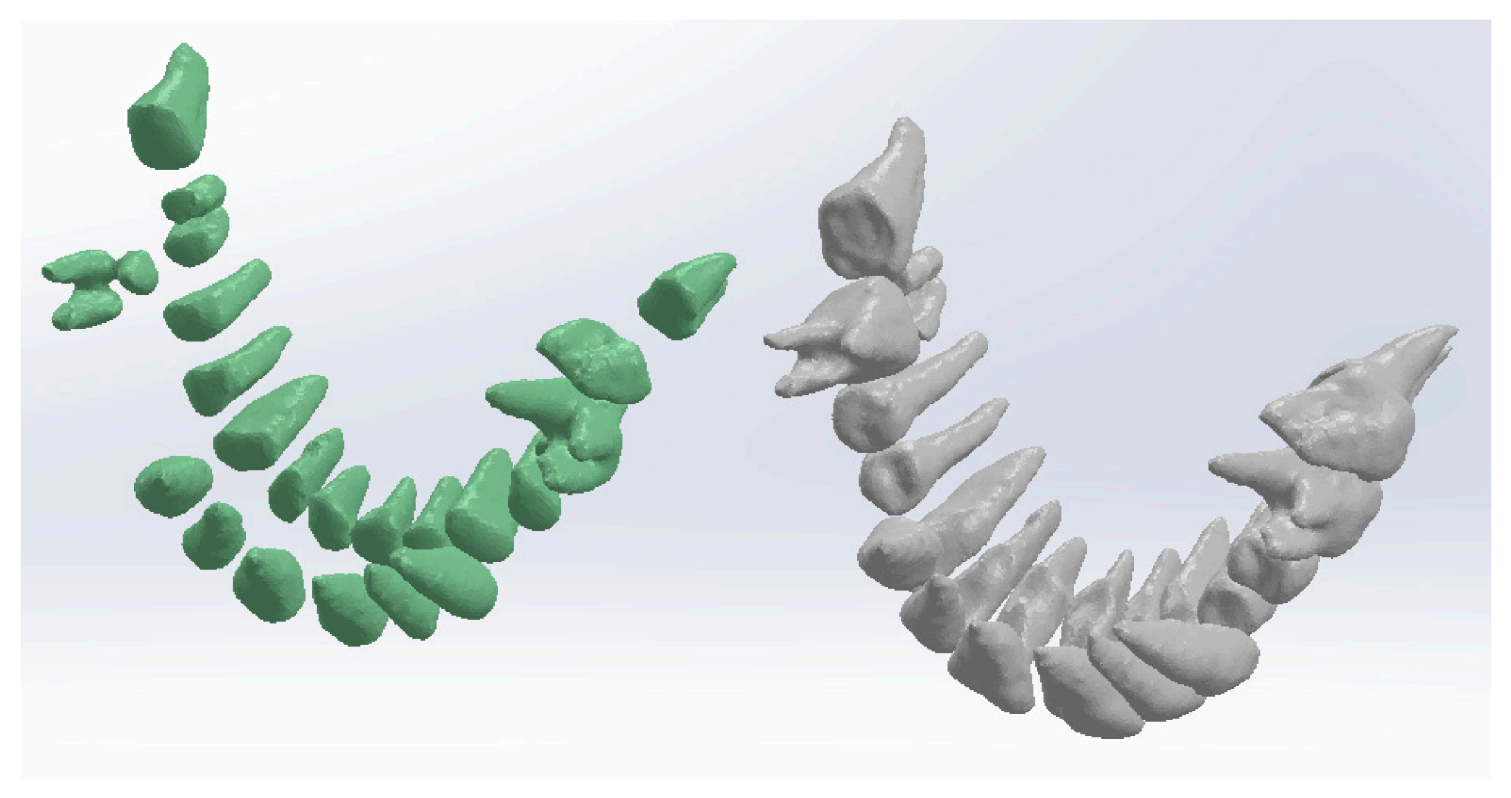
Dental and ligament models in the Assembly module.

Next, the models of the dentition and the dental ligaments were loaded into the Assembly module of SolidWorks, as shown in Figure 18.

**Figure 19:**
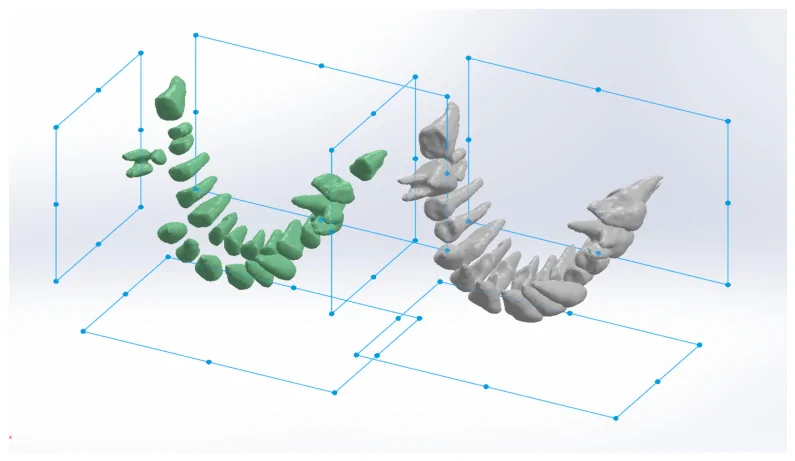
Three-dimensional models in the six reference planes.

The models are derived from the same set of CBCT images and, for this reason, have identical coordinate systems. Figure 19 shows the models and the six reference planes, three for each model.

**Figure 20:**
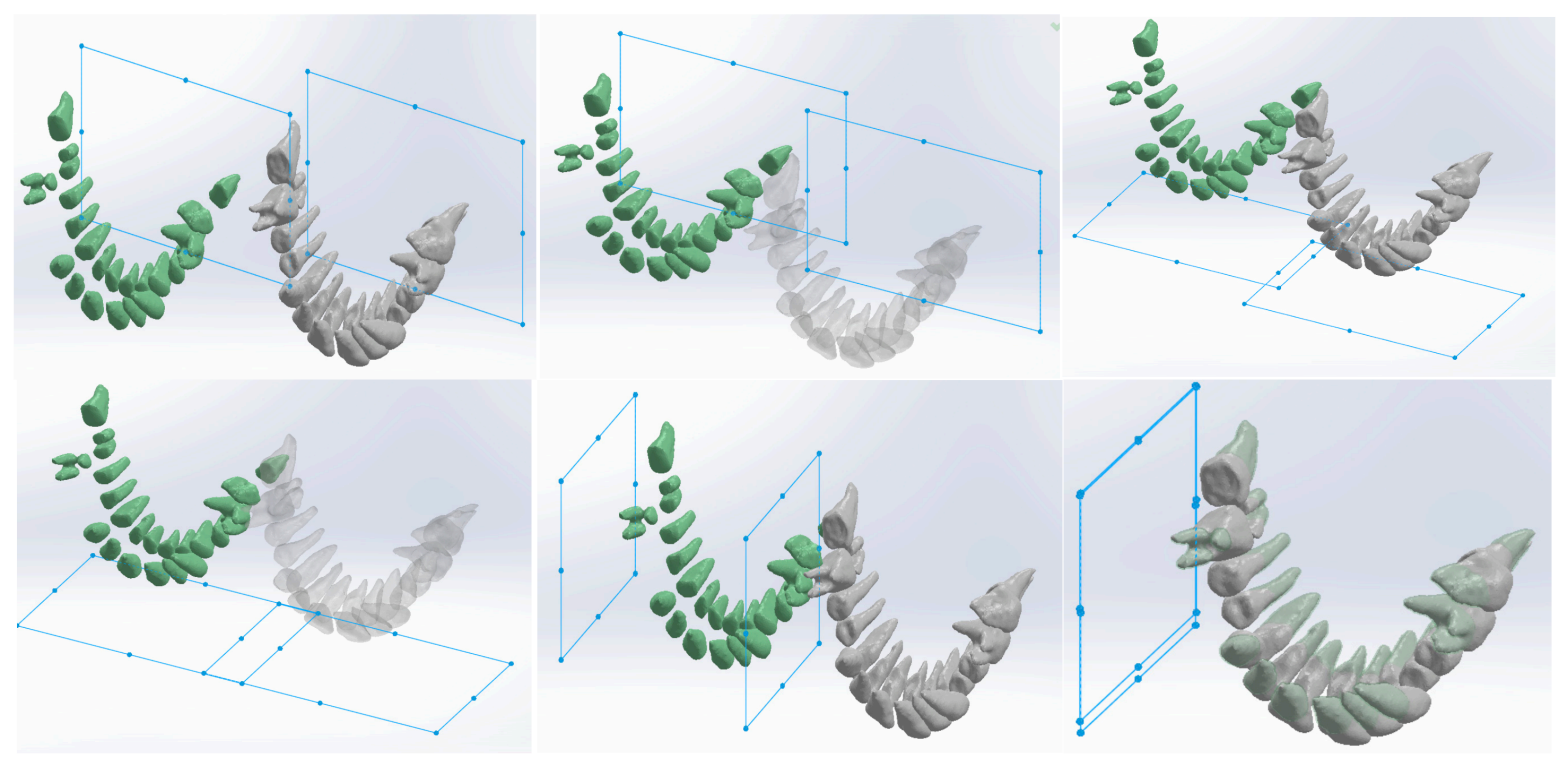
Alignment of reference planes.

To ensure that the models are in the correct position, the corresponding reference planes were aligned. Figure 20 shows the three alignment operations based on the coincidence of the planes.

**Figure 21:**
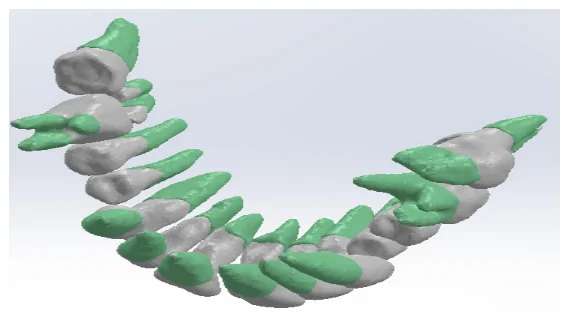
The result of aligning with the reference planes.

The result of the plane alignment operation is shown in Figure 21.

**Figure 22:**
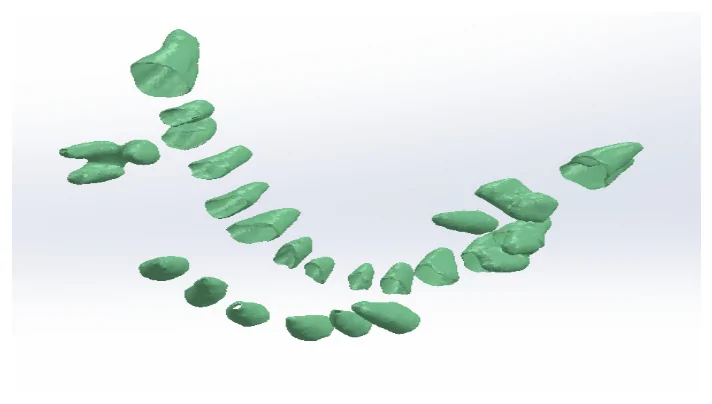
The result of applying the volume subtraction.

In the context of the assembly, a volume subtraction operation was performed between the ligament and the teeth, resulting in the dental ligament cavities, as shown in Figure 22.

**Figure 23:**
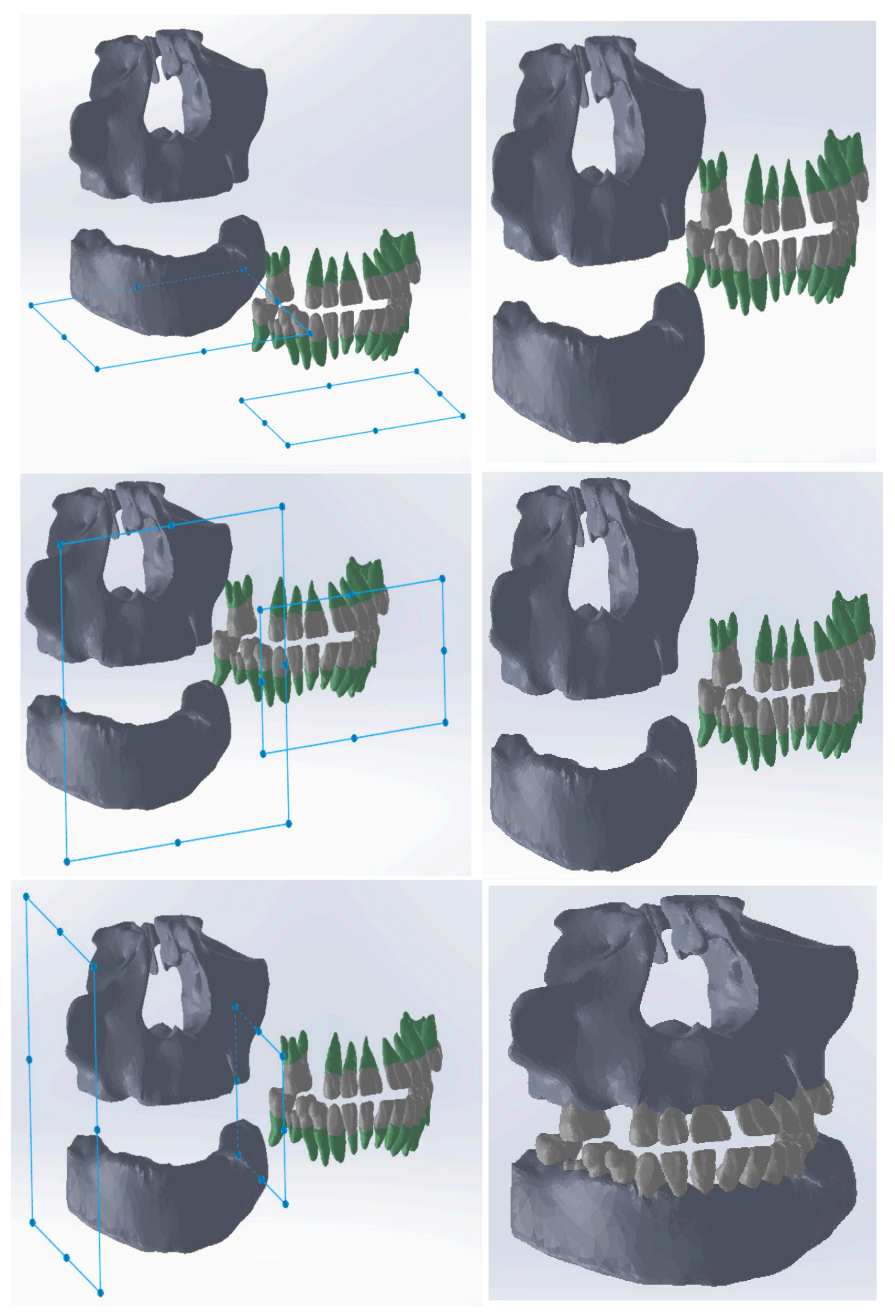
Alignment of the reference planes for the bone components.

Next, in the SolidWorks Assembly module, the model of the two bone components was loaded. Similar operations were performed to align the reference planes for the models, as shown in Figure 23.

**Figure 24:**
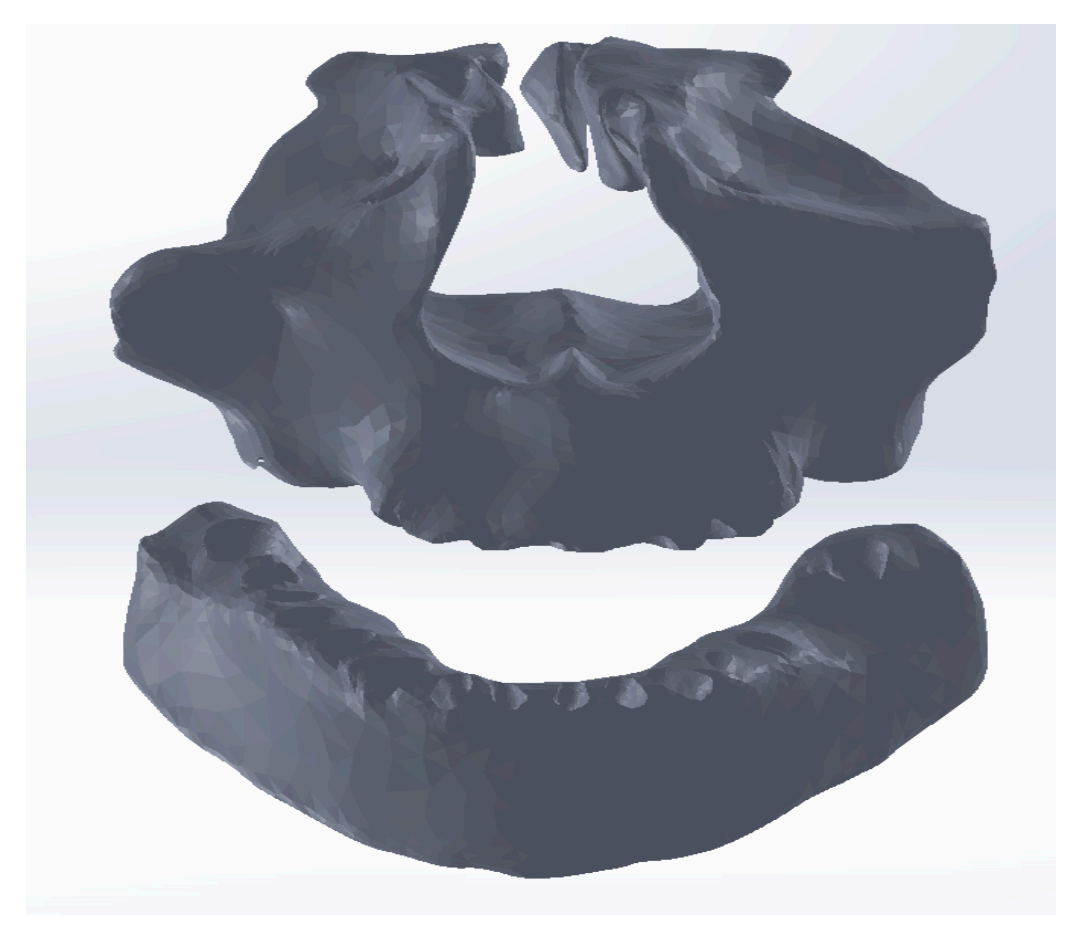
Generating dental alveoli in the two bone components.

Next, two volume subtractions were performed between the bone components and the dentition and dental ligaments, resulting in the dental alveoli within the bone components, as shown in Figure 24.

#### 2.6.5. Three-Dimensional Models of the Bracket-Type Components and the Orthodontic Adhesive

**Figure 25:**
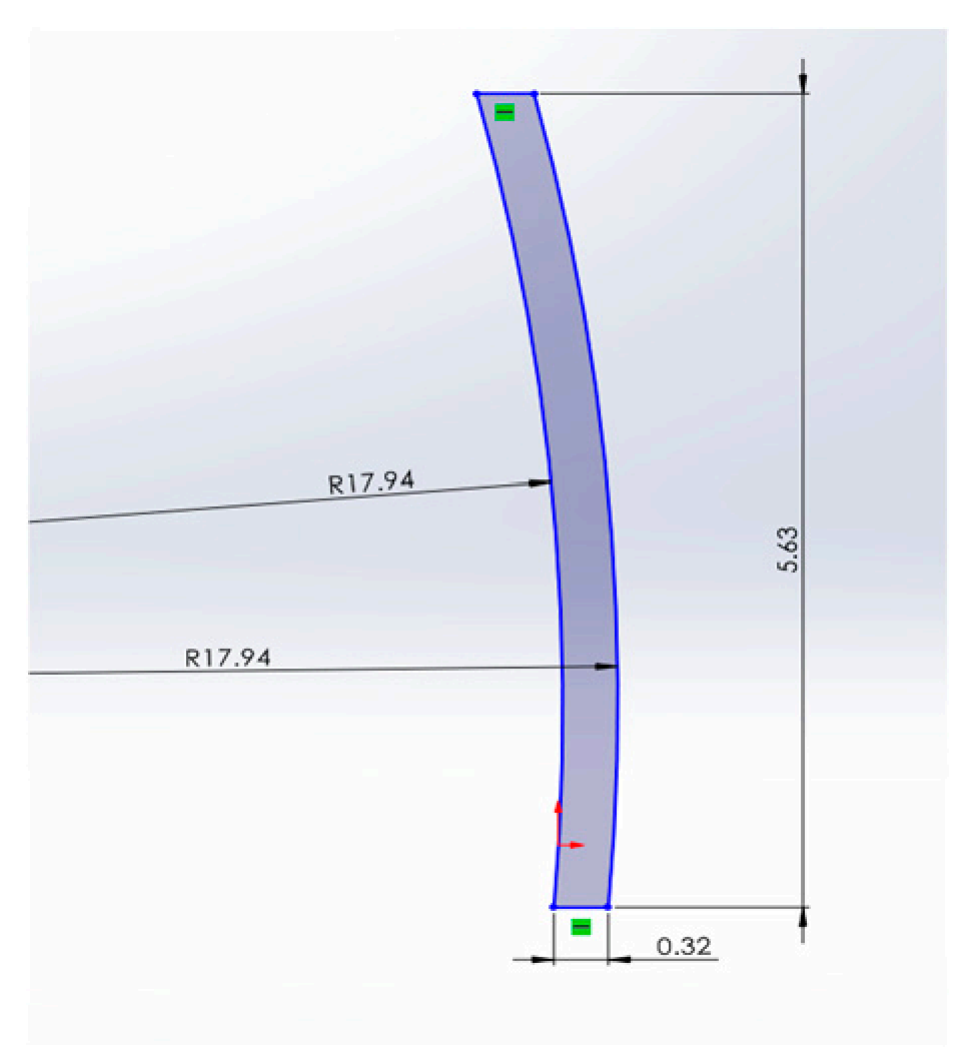
Initial reference planes.

Next, the bracket-type components were modeled using CAD and direct engineering techniques. The following section details the steps involved in generating a model of a bracket for the mandibular incisors. The sketch shown in Figure 25 was drawn on one of the initial reference planes.

**Figure 26:**
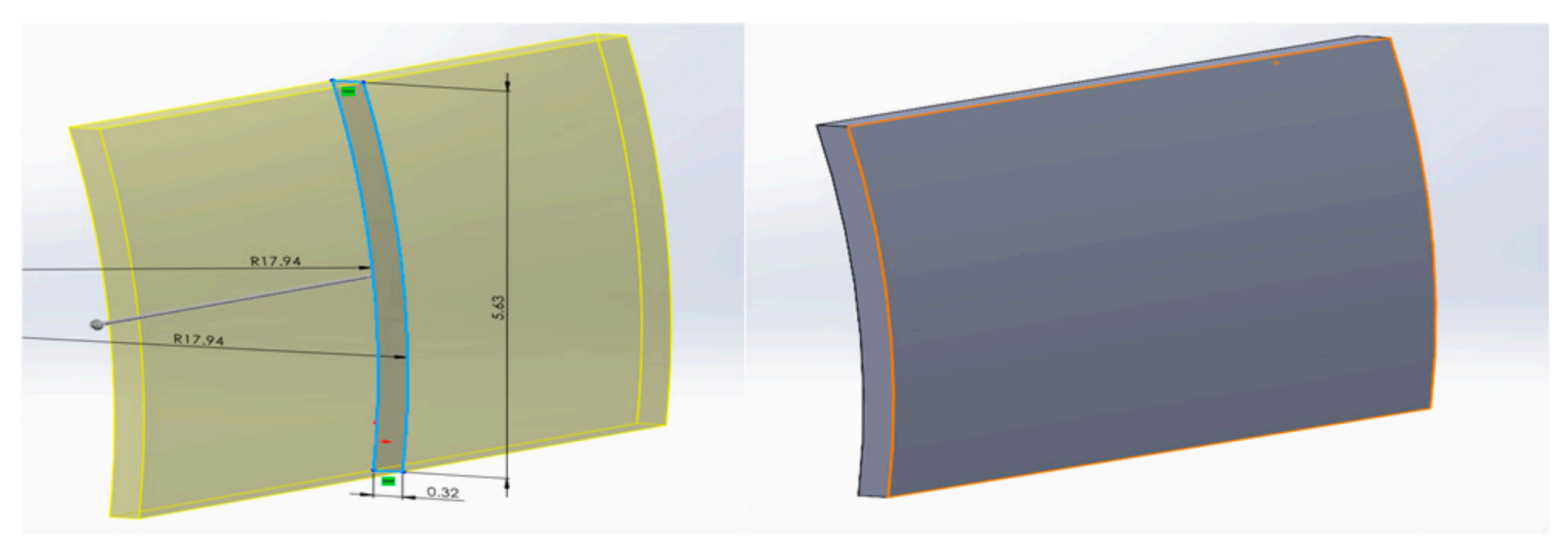
Base form of the model.

Since this is a symmetrical component, we chose to model an extruded solid with a midplane, as shown in Figure 26.

**Figure 27:**
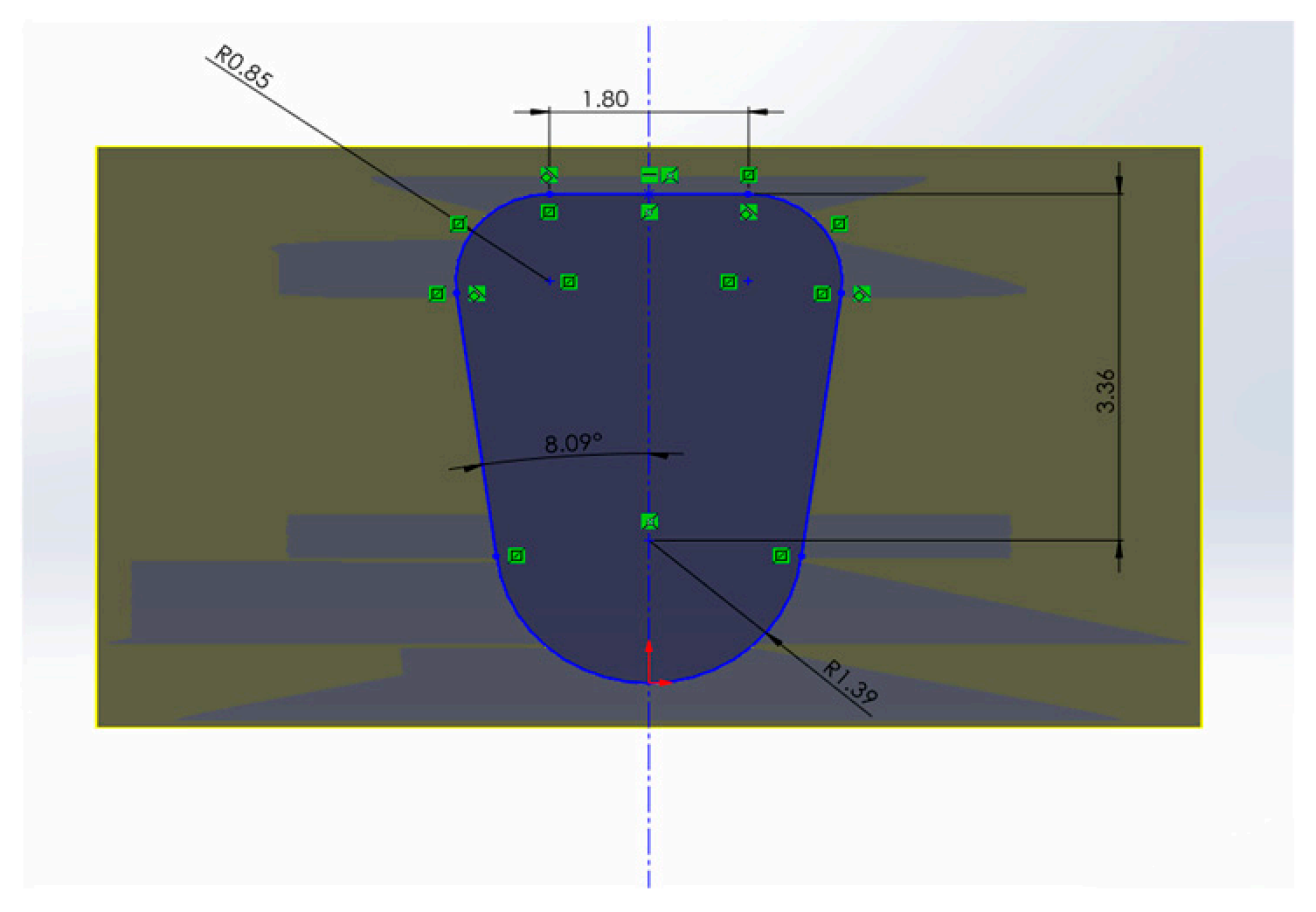
Sketch drawn in a reference plane perpendicular to the original.

The sketch shown in Figure 27 was drawn in a reference plane perpendicular to the original one.

**Figure 28:**
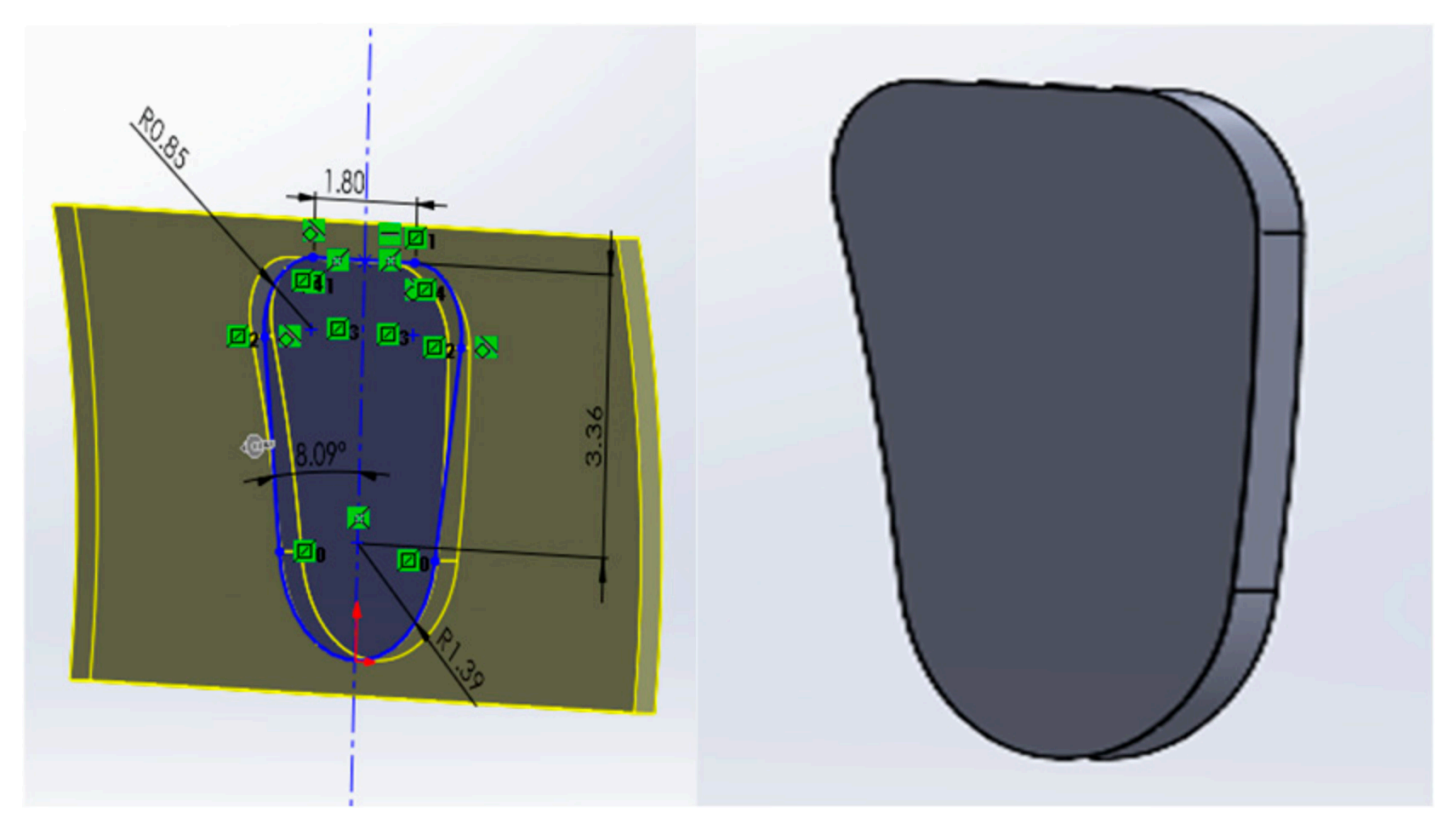
Cut-off in the base form.

Using this sketch, the basic shape was cut out from the outside (flip side to cut-enabled), as shown in Figure 28.

**Figure 29:**
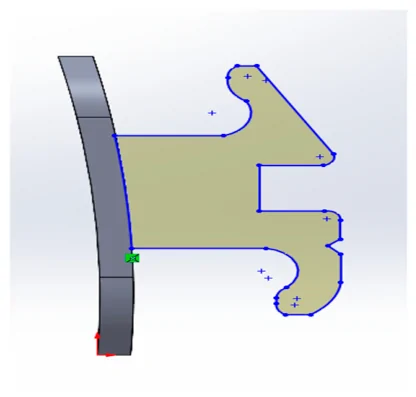
Sketch of the initial plane.

In the initial planes, the sketch from Figure 29 was drawn.

**Figure 30:**
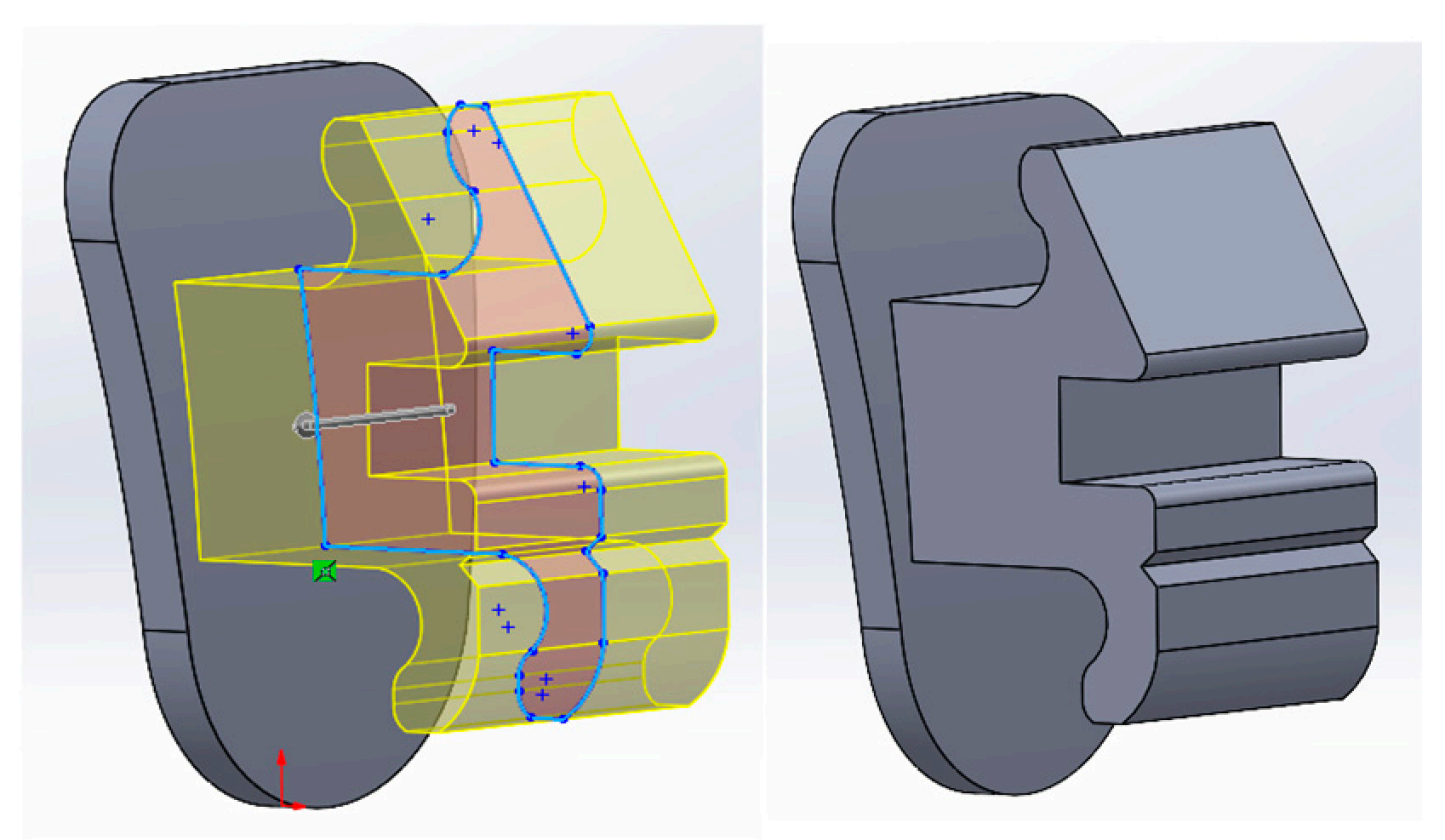
Extruded shape.

This sketch was used to add an extrusive plane using Mid Plane, as shown in Figure 30.

**Figure 31:**
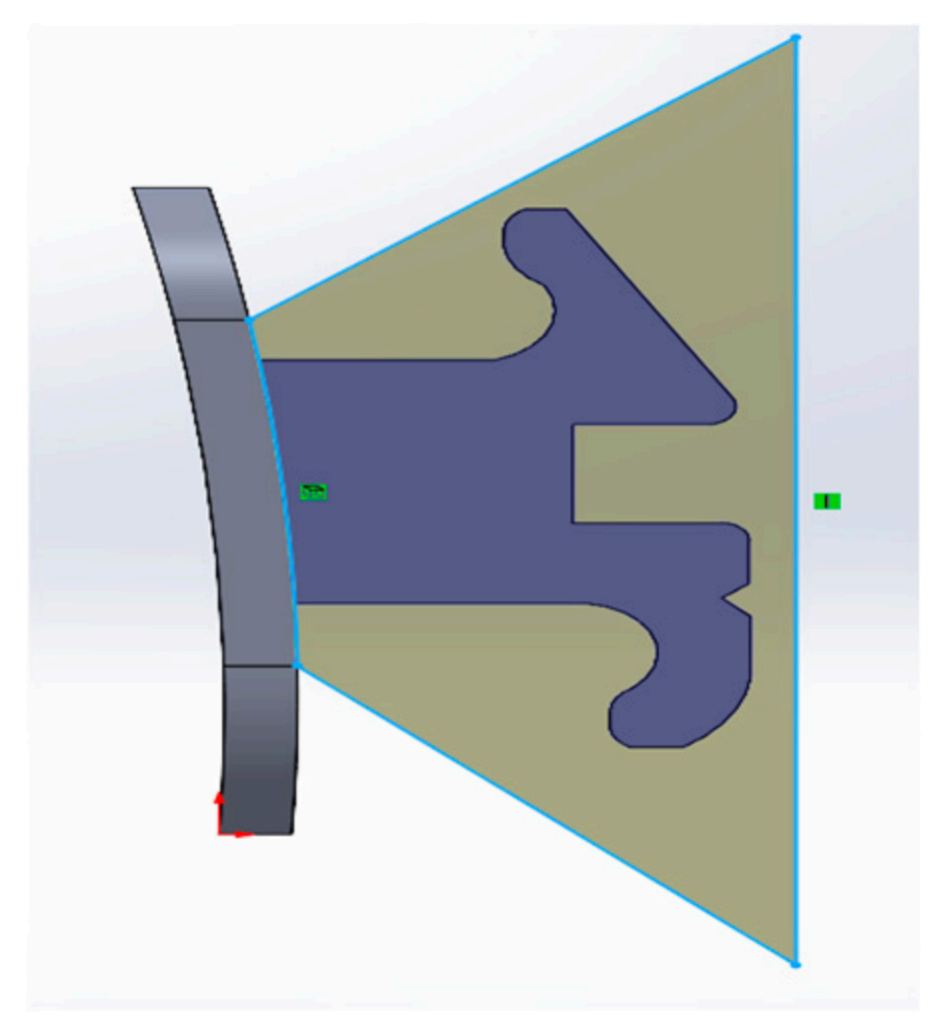
Sketch drawn in the same plane.

In the same plane, the sketch from Figure 31 was drawn.

**Figure 32:**
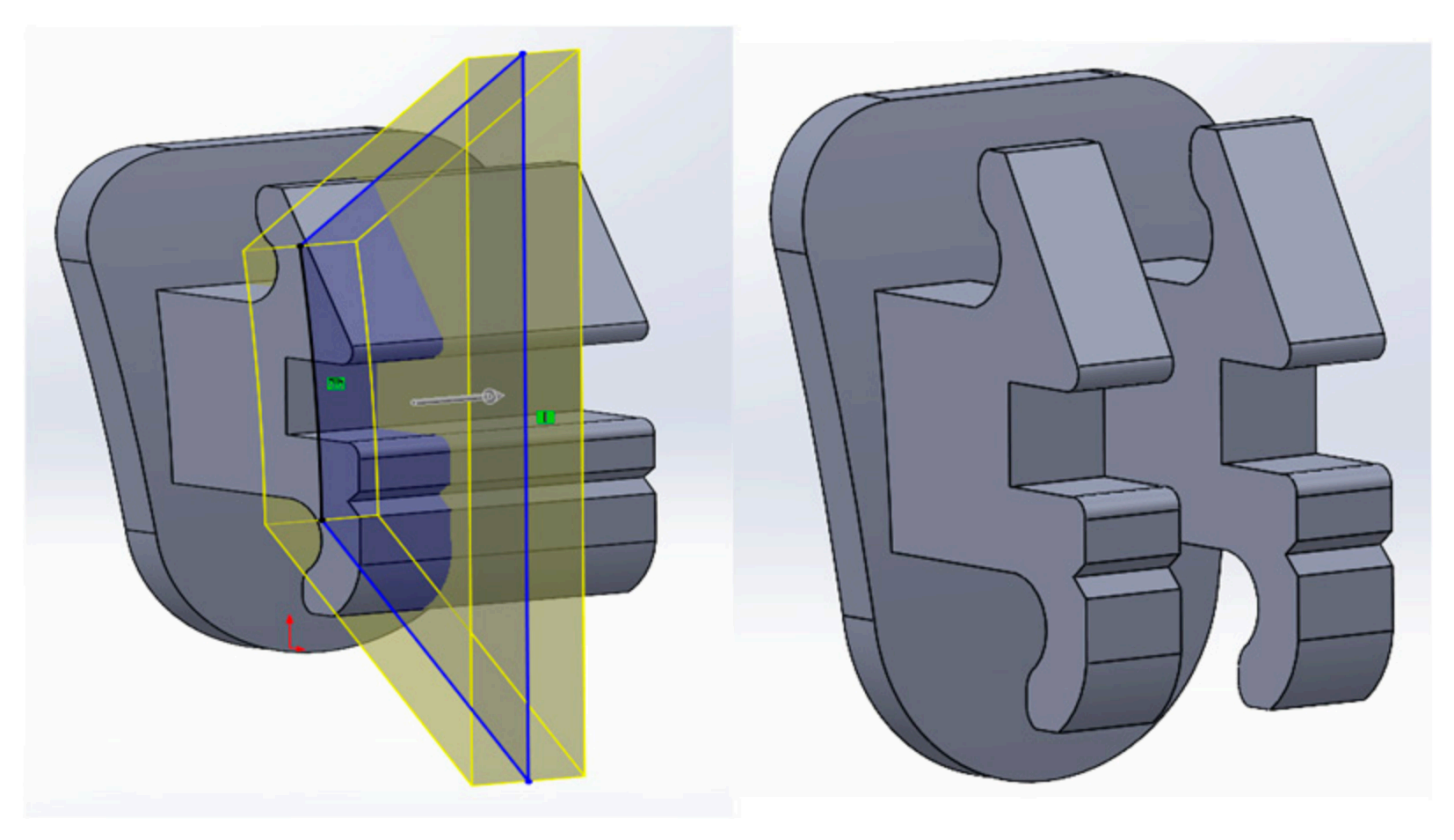
Median cut.

Using the sketch, a cut was created using the Mid Plane option, as shown in Figure 32.

**Figure 33:**
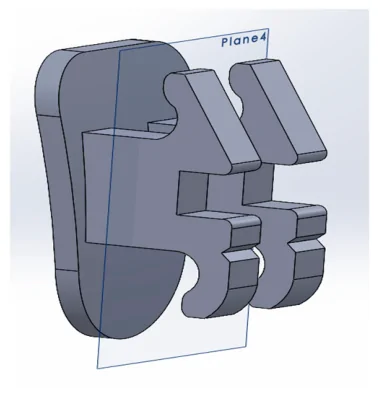
Defining a reference plane.

Next, a reference plane parallel to the initial one was defined, as shown in Figure 33.

**Figure 34:**
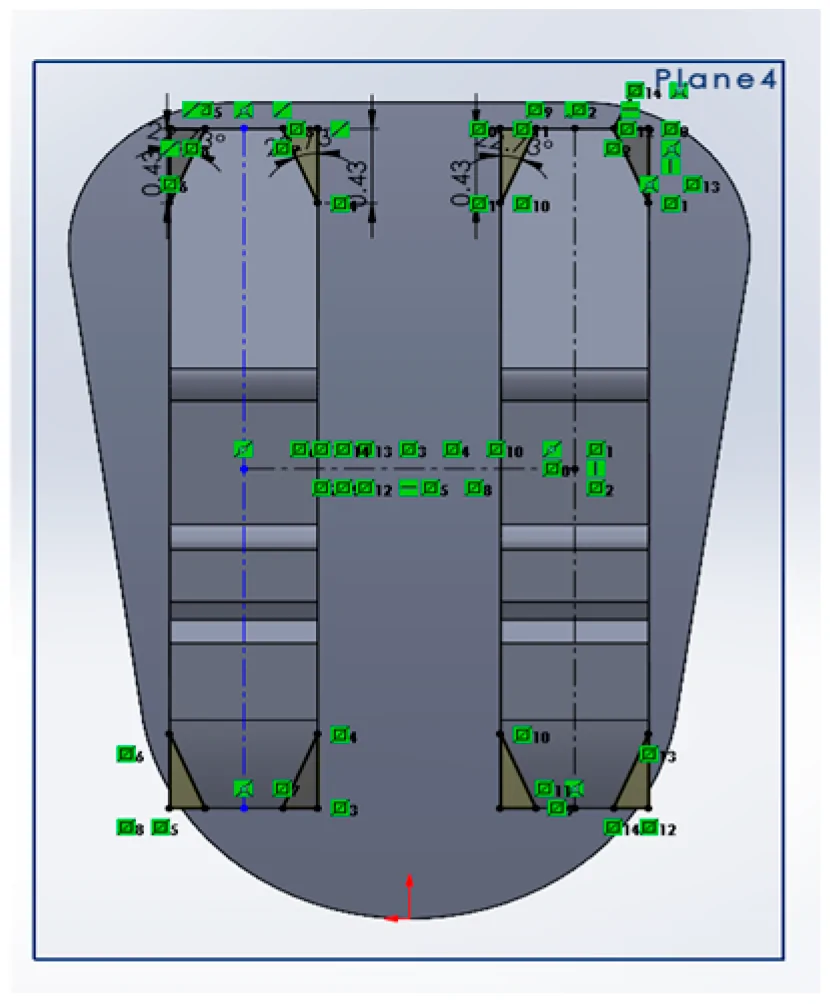
Sketches in the defined plane.

In this plane, sketches from Figure 34 were drawn.

**Figure 35:**
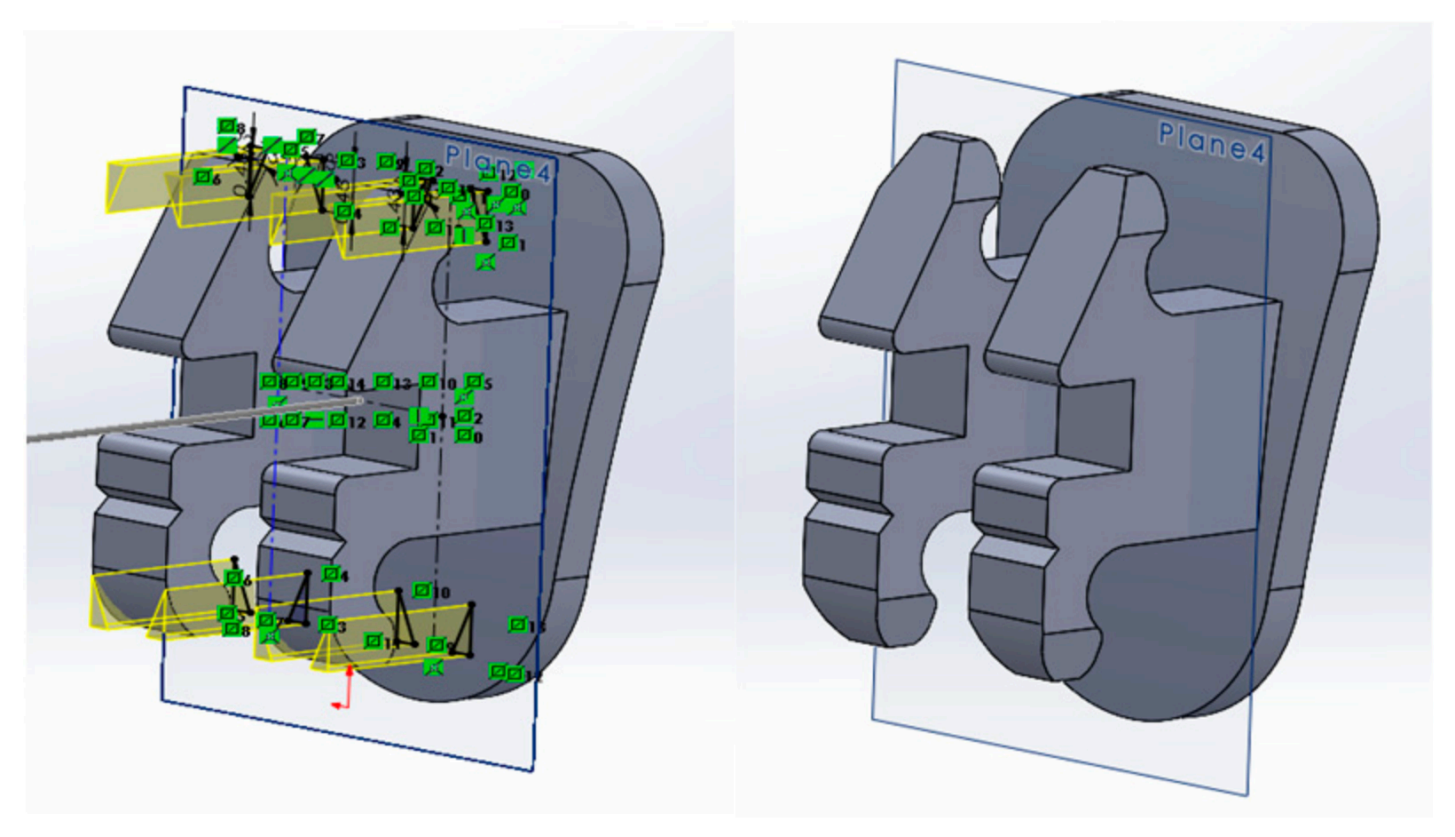
Cutouts generated using the Cut-Extrude command.

These sketches were used to create the cut features shown in Figure 35.

**Figure 36:**
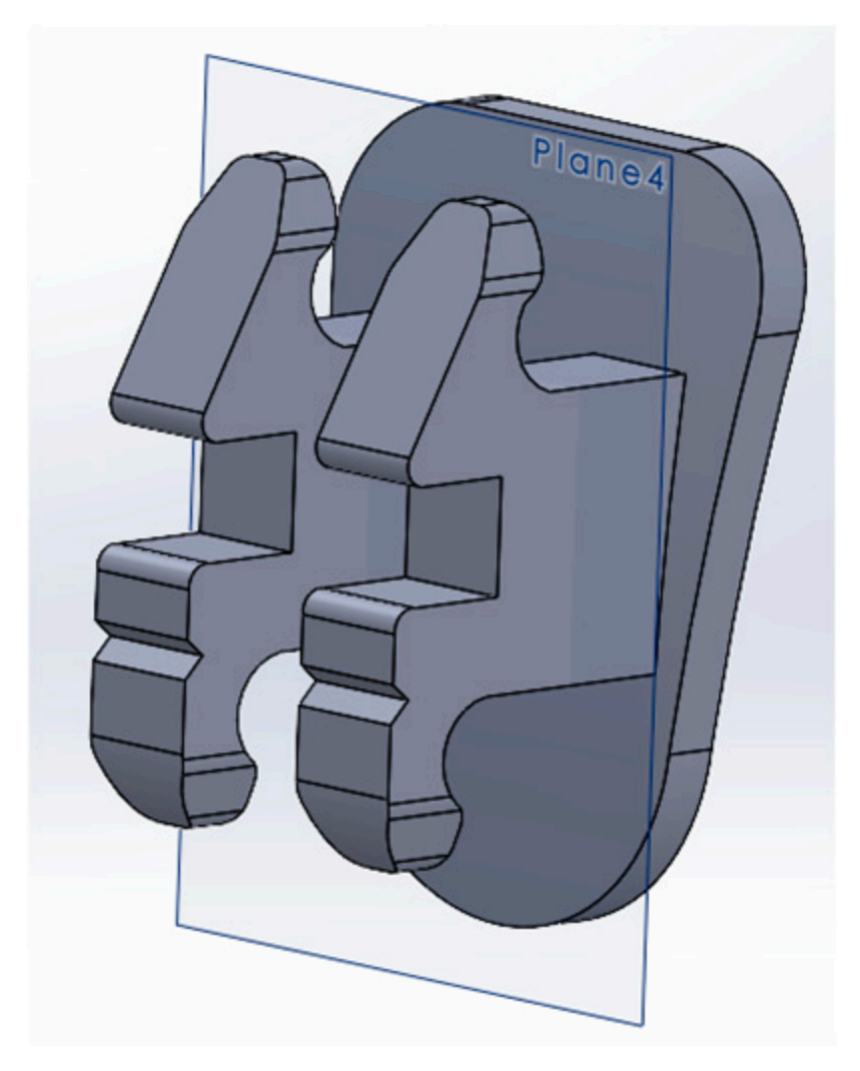
Fillet shapes.

Next, additional fillet shapes were generated, as shown in Figure 36.

**Figure 37:**
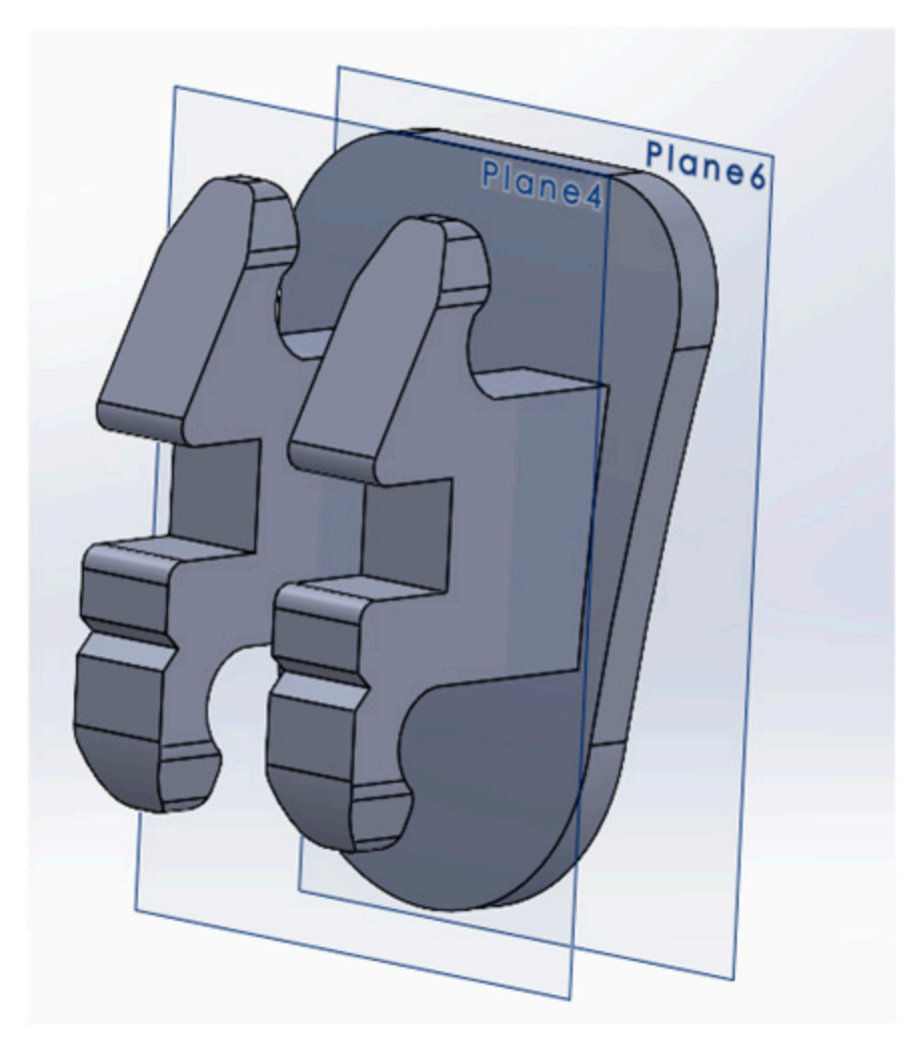
Defining a reference plane.

Next, a new reference plane was defined at a distance from the base of the bracket-type component, as shown in Figure 37.

**Figure 38:**
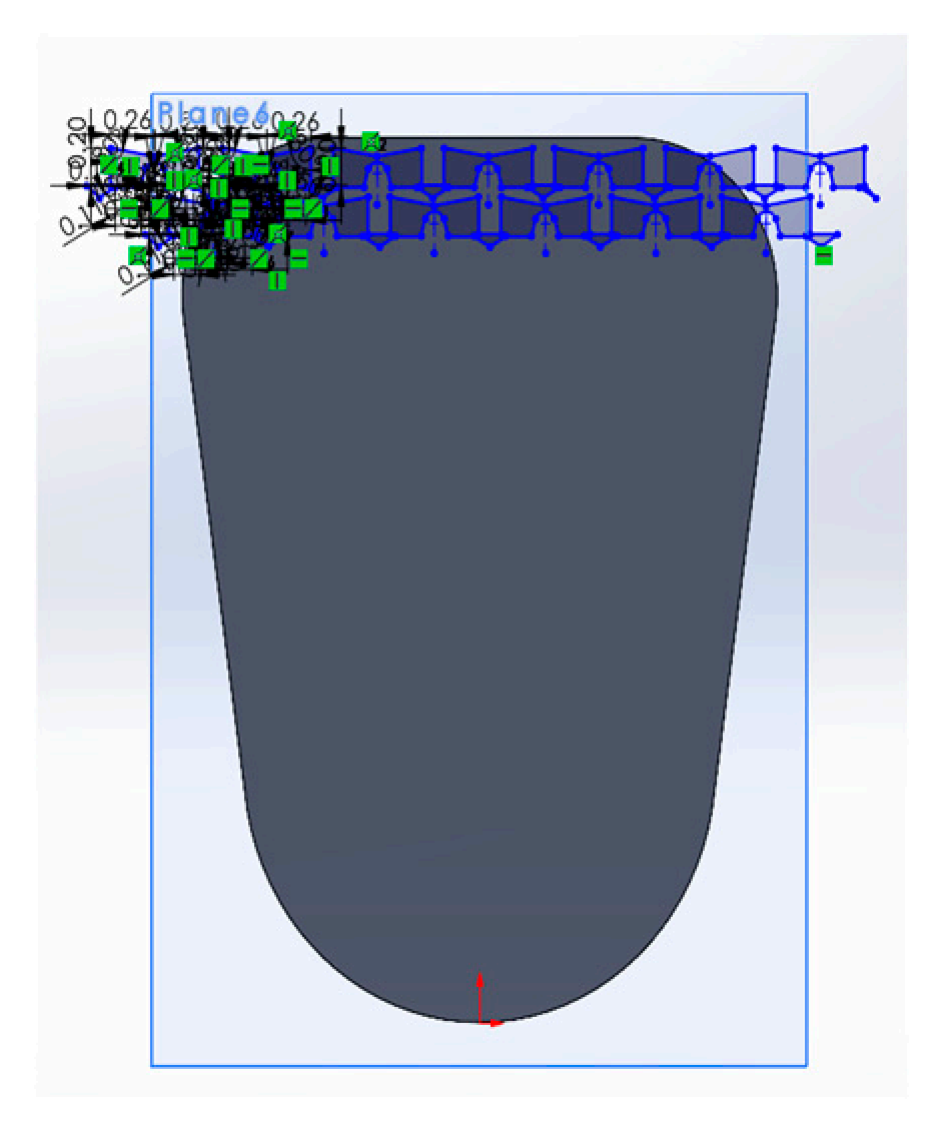
Sketch in the newly defined plane.

Next, the sketch shown in Figure 38 was defined in this plane.

**Figure 39:**
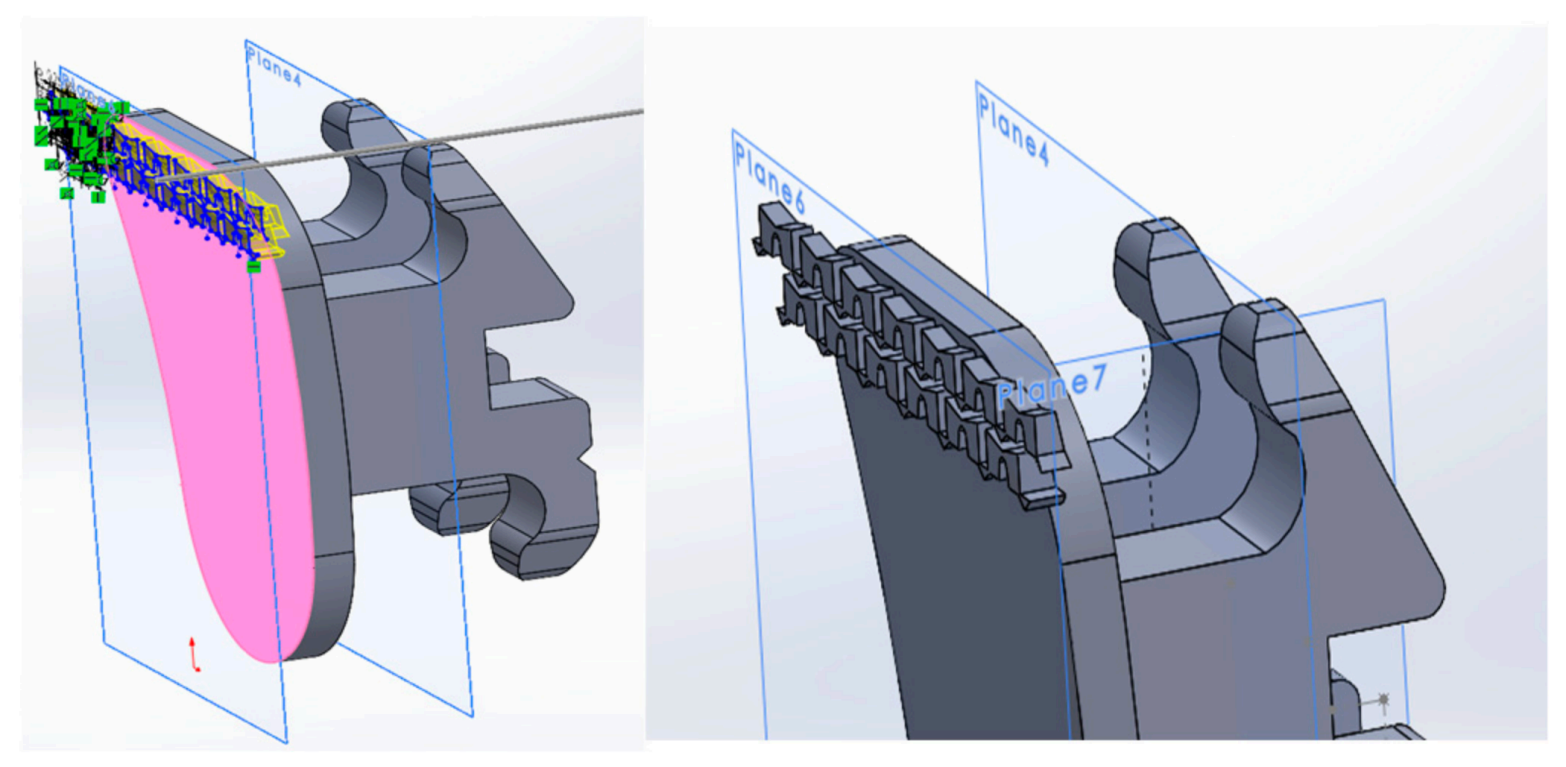
Extruded shape.

This sketch was used to create a new extruded shape, as shown in Figure 39.

**Figure 40:**
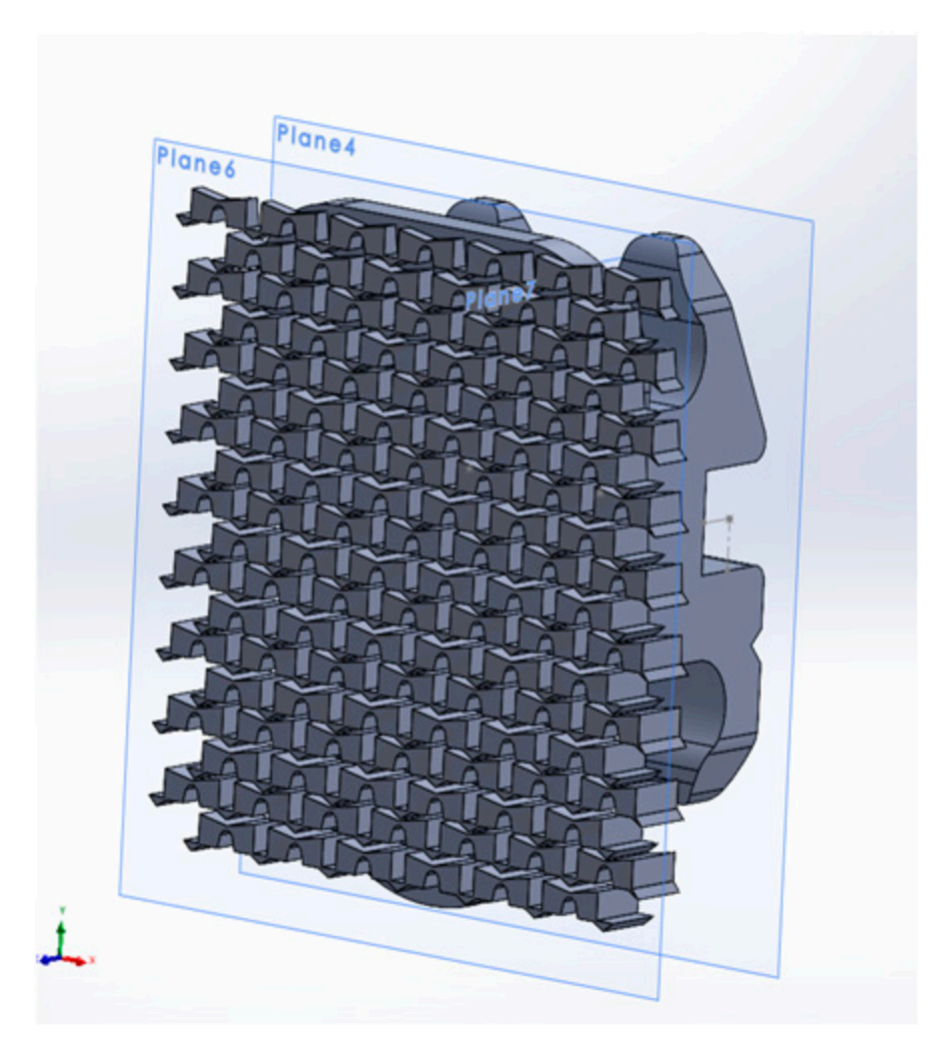
Linear Pattern Multiplication.

This extruded shape was duplicated using a Linear Pattern command, and the resulting model is shown in Figure 40.

**Figure 41:**
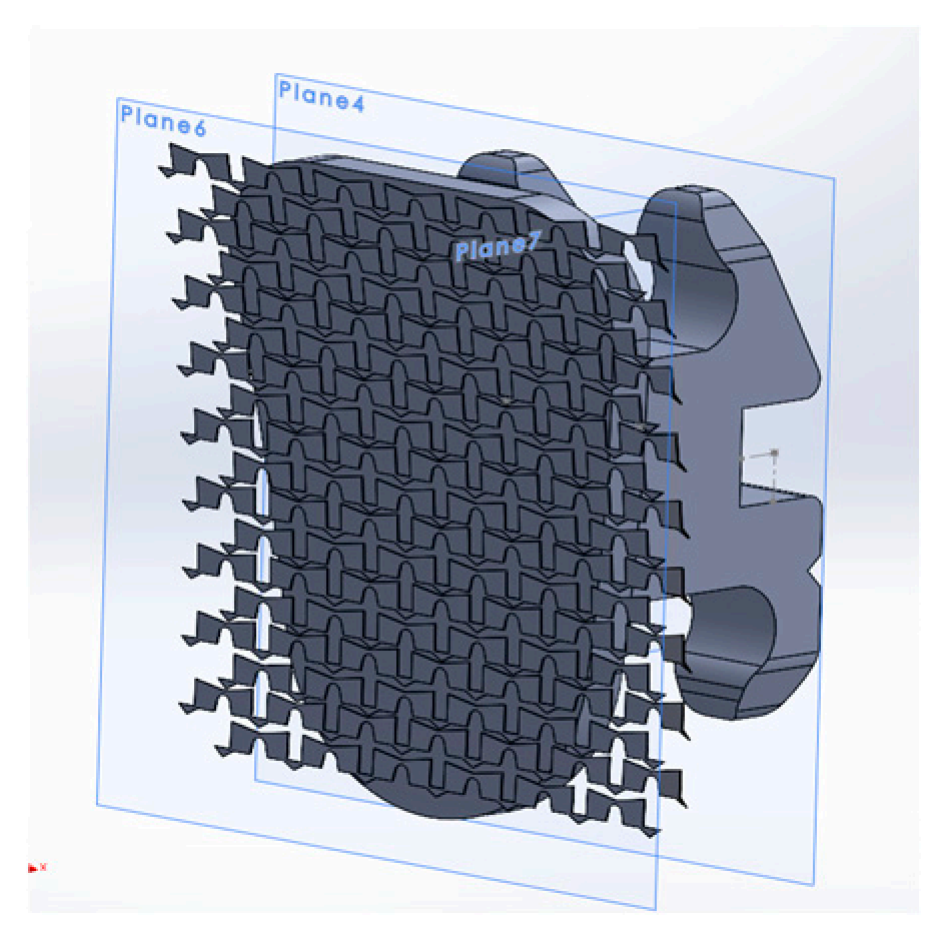
Cutting out the pattern-type shape.

This shape was cut out using a surface located at a distance from the base of the bracket-type component, and the result is shown in Figure 41.

**Figure 42:**
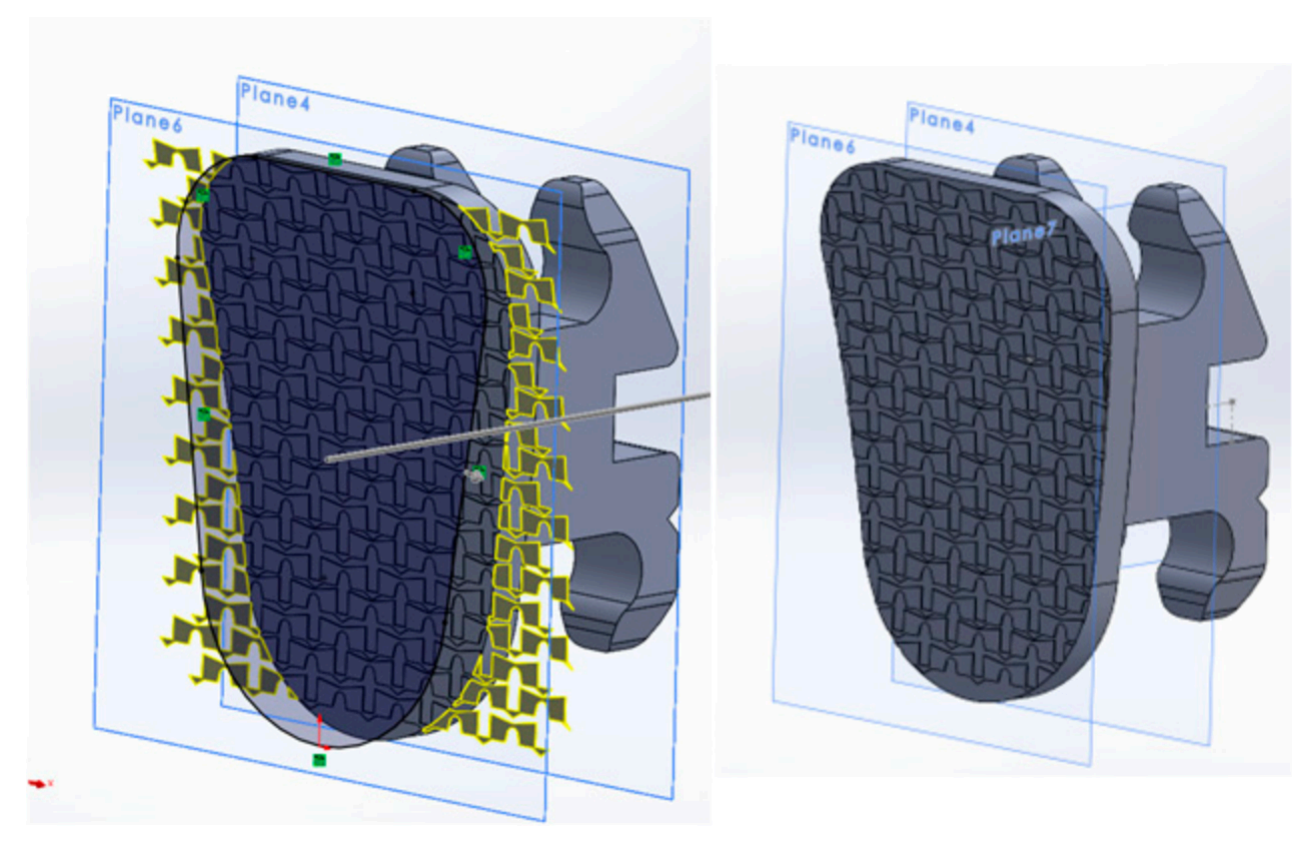
Outline cutting.

Using an outline taken from the sole of the pattern, the pattern was cut out from the outside, as shown in Figure 42.

**Figure 43:**
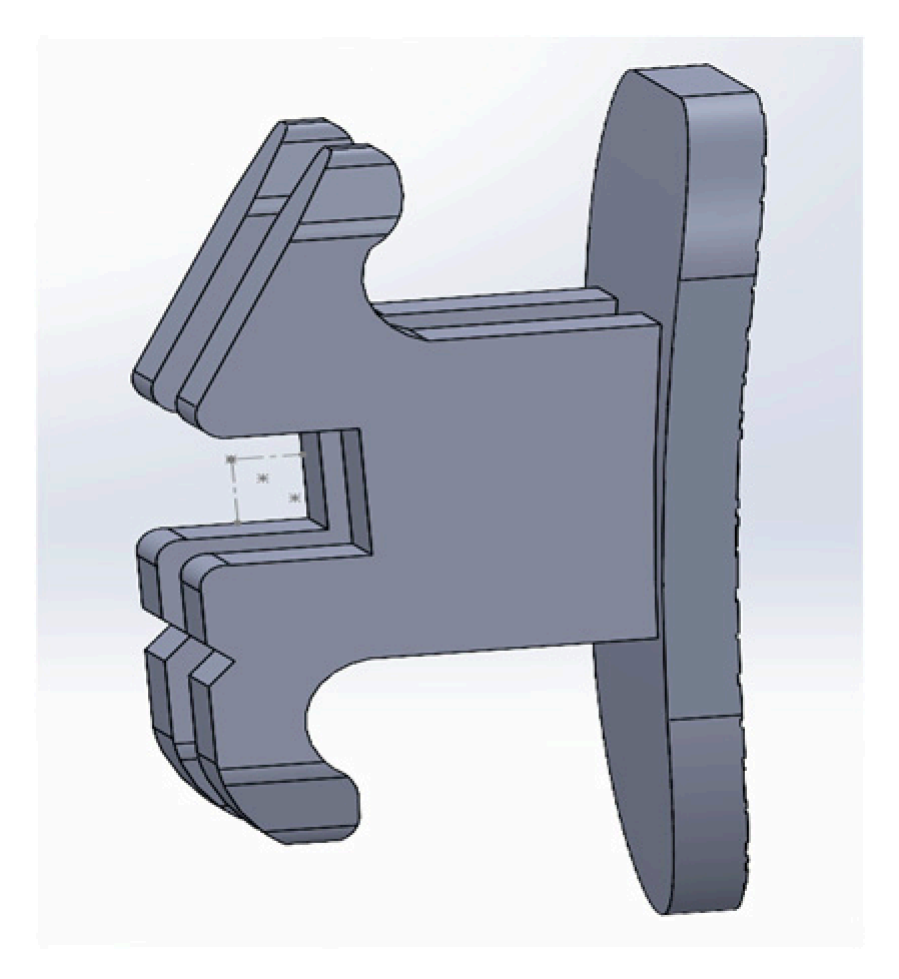
Defining the three points along the orthodontic archwire path.

To generate the orthodontic wire model, three points were drawn on the bracket component model (Figure 43).

**Figure 44:**
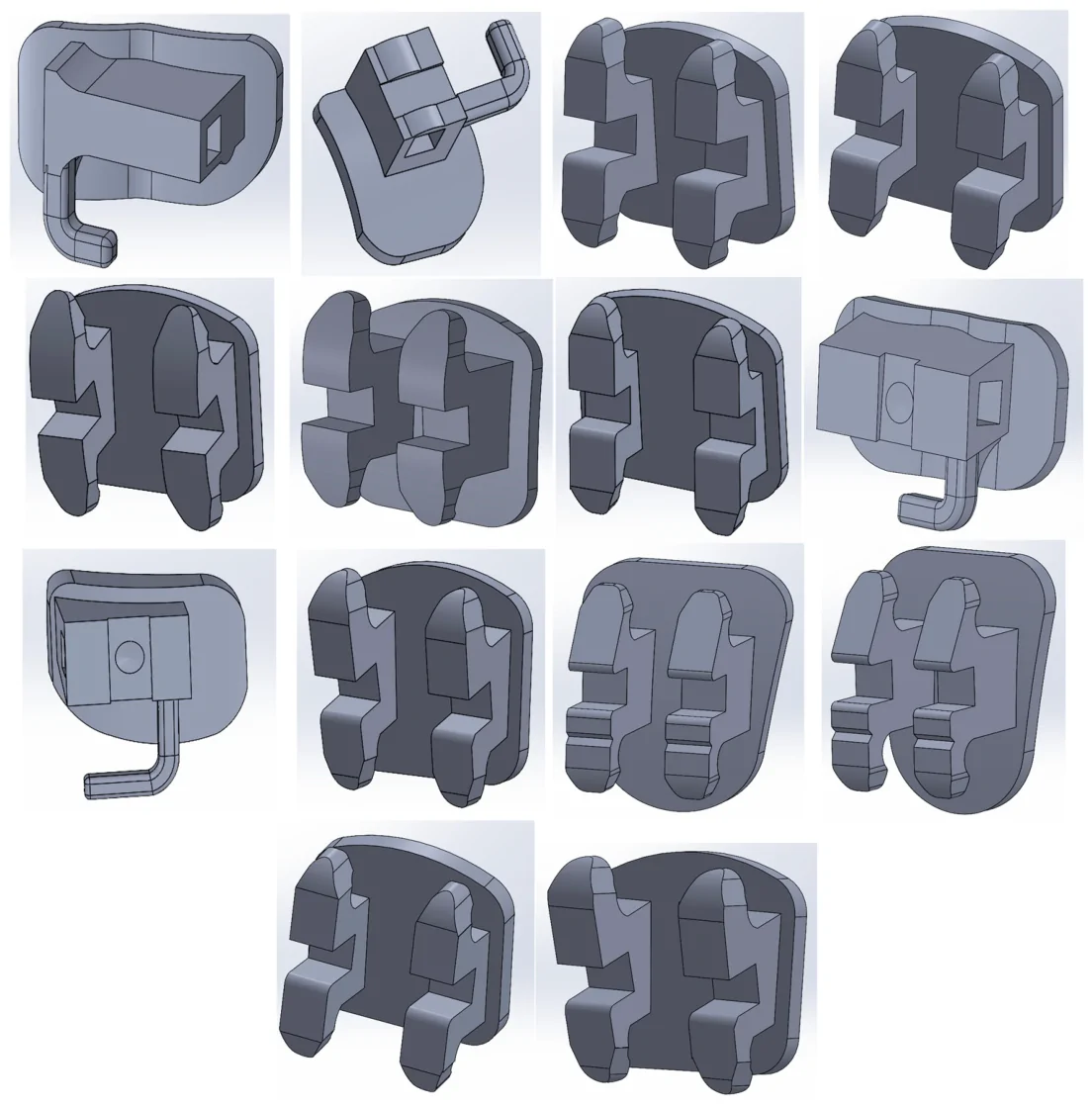
Models of the bracket-type components used in the study.

Models of all bracket-type components were created using similar techniques (Figure 44). Mirroring techniques were also used for the symmetrical components.

**Figure 45:**
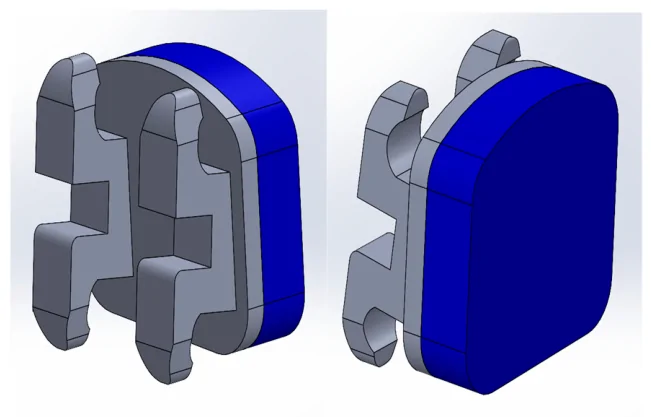
The initial adhesive model.

To model the orthodontic adhesive, the contour of the bracket-type component’s base was copied and then extruded, as shown in Figure 45.

**Figure 46:**
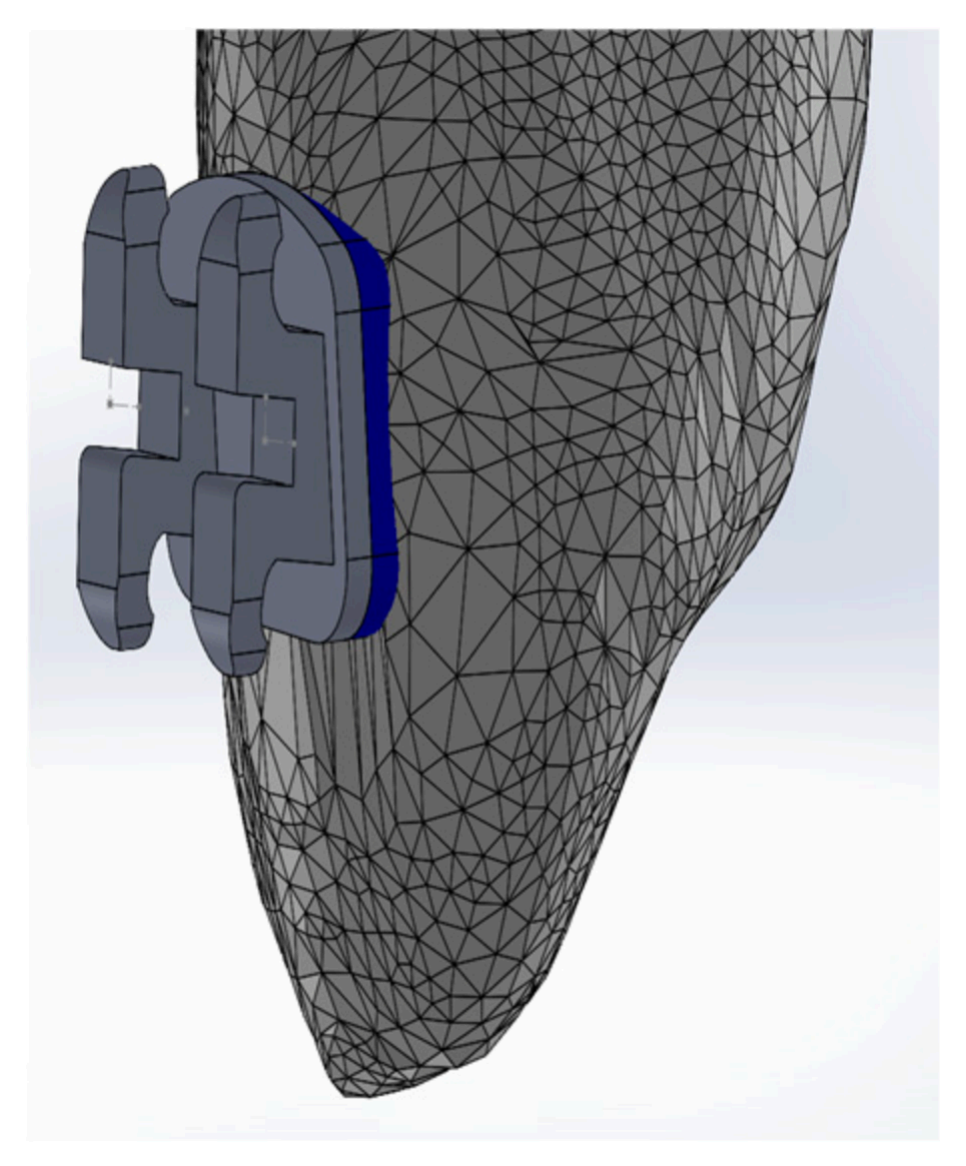
Placement of the adhesive and bracket-type component on a canine tooth.

The bracket-type components were positioned on the corresponding teeth; Figure 46 shows, for example, their placement on a canine.

**Figure 47:**
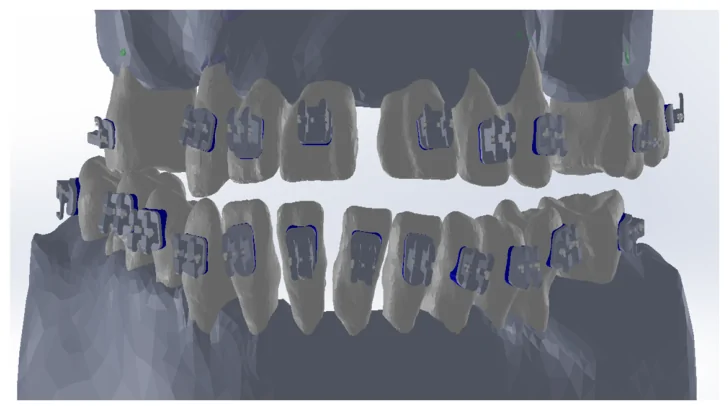
Positioning of bracket-type components.

All bracket-type components were positioned in a similar manner with the adhesive, resulting in the model shown in Figure 47.

By applying a volume reduction between the adhesive models and each individual tooth, the final adhesive models were obtained.

#### 2.6.6. Three-Dimensional Models of Orthodontic Wires

**Figure 48:**
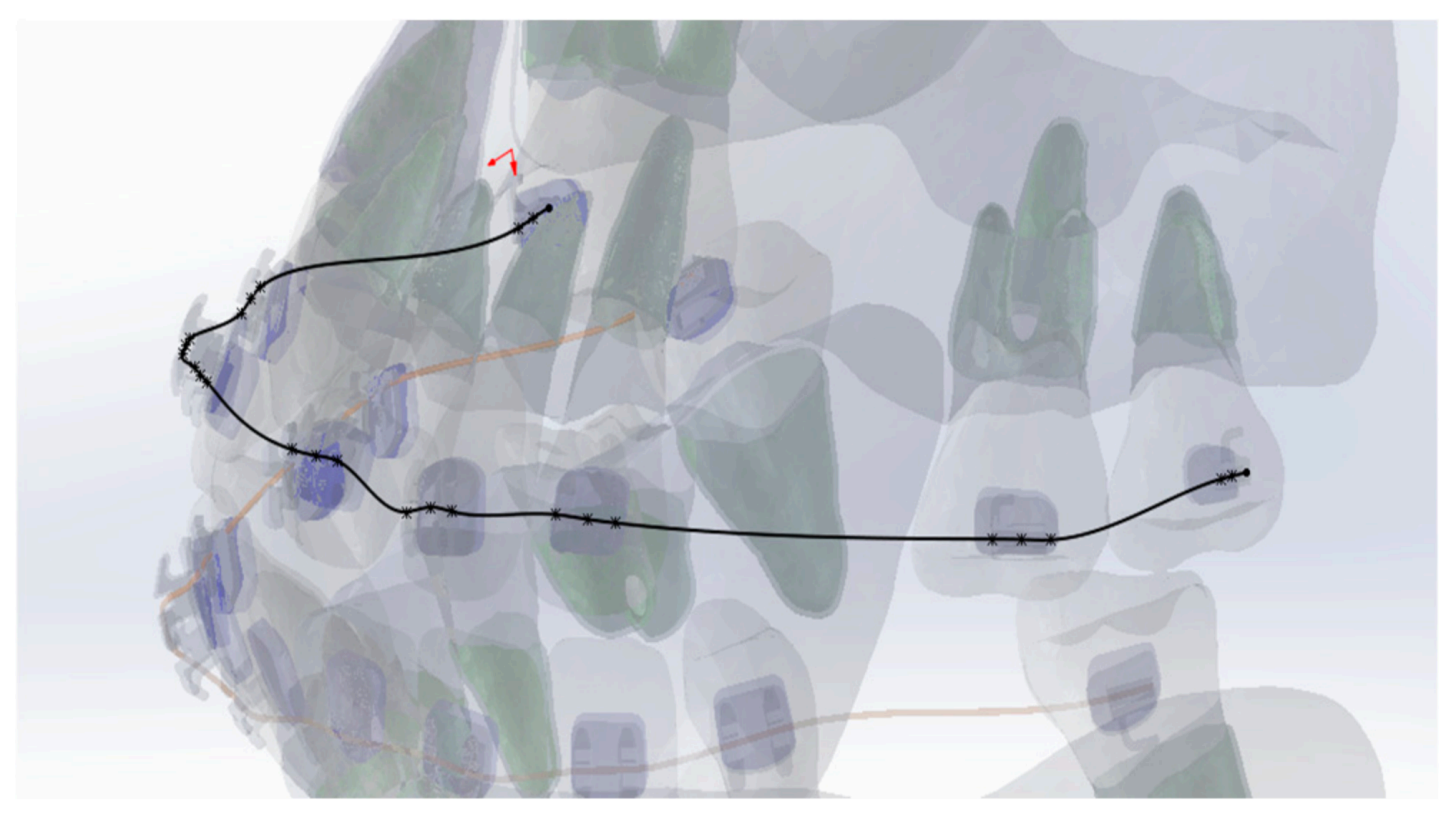
Spline Curve for the jaw.

Next, using the points corresponding to the bracket-type components, a spline curve was drawn for the maxilla, as shown in Figure 48.

**Figure 49:**
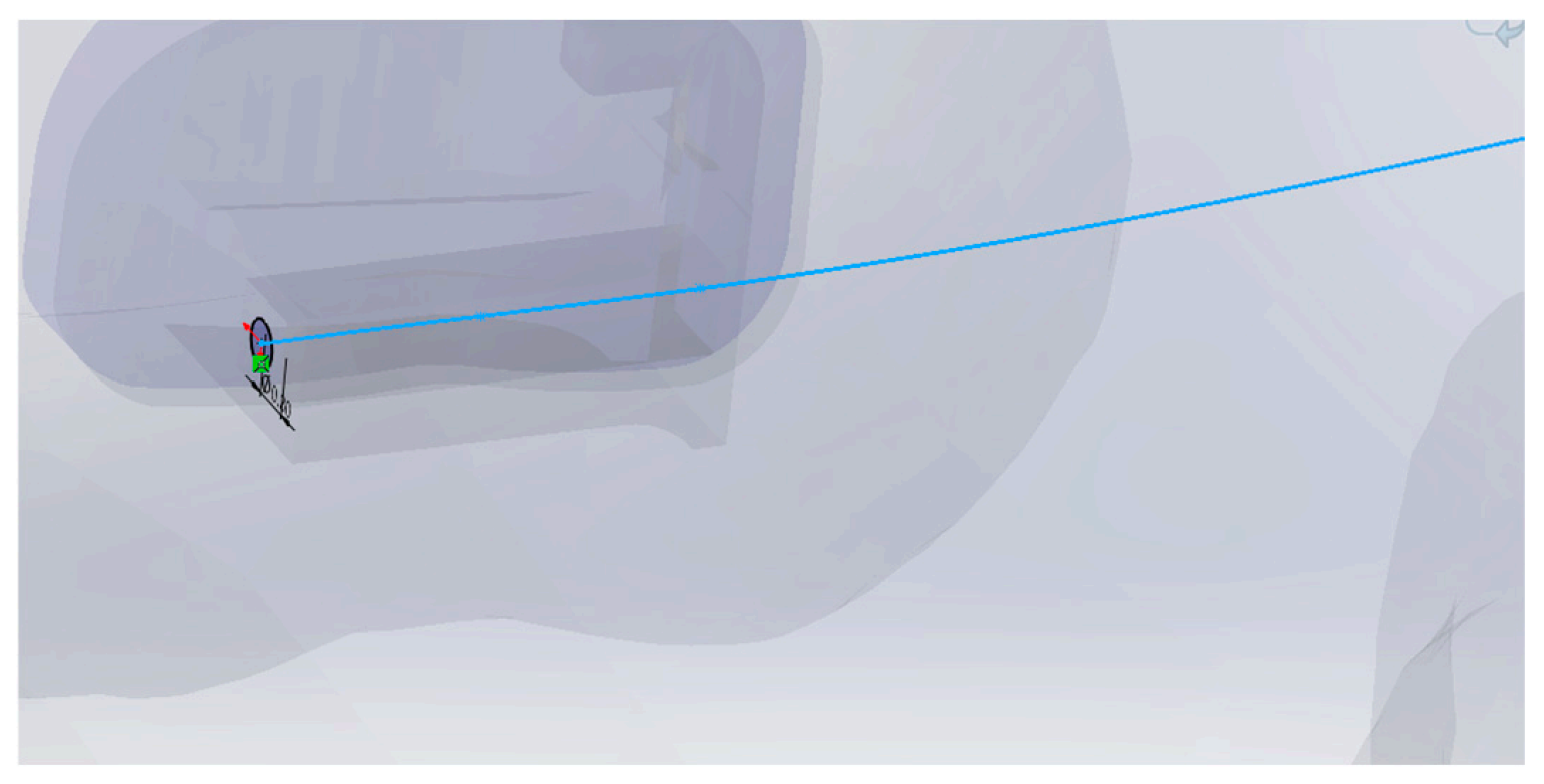
A circle drawn in a plane perpendicular to the Spline Curve.

A circle with a diameter of 0.012 inches (approx. 0.3 mm) was drawn in a plane perpendicular to this curve, as shown in Figure 49.

**Figure 50:**
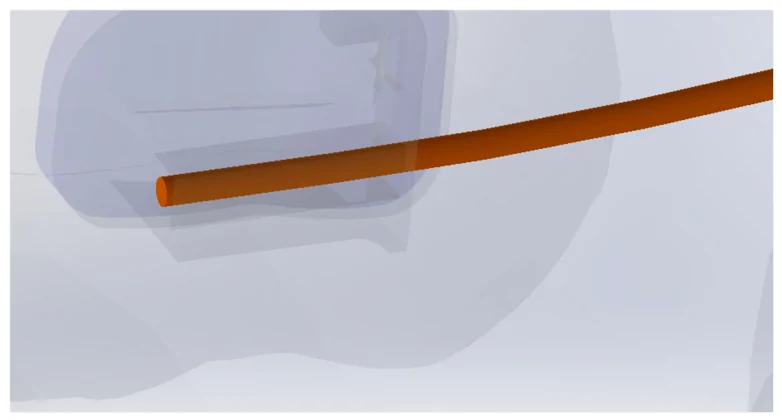
The virtual solid modelling of the orthodontic maxillary wire.

Next, using a Sweep feature, the virtual solid modeling the orthodontic wire for the maxilla was defined (Figure 50).

**Figure 51:**
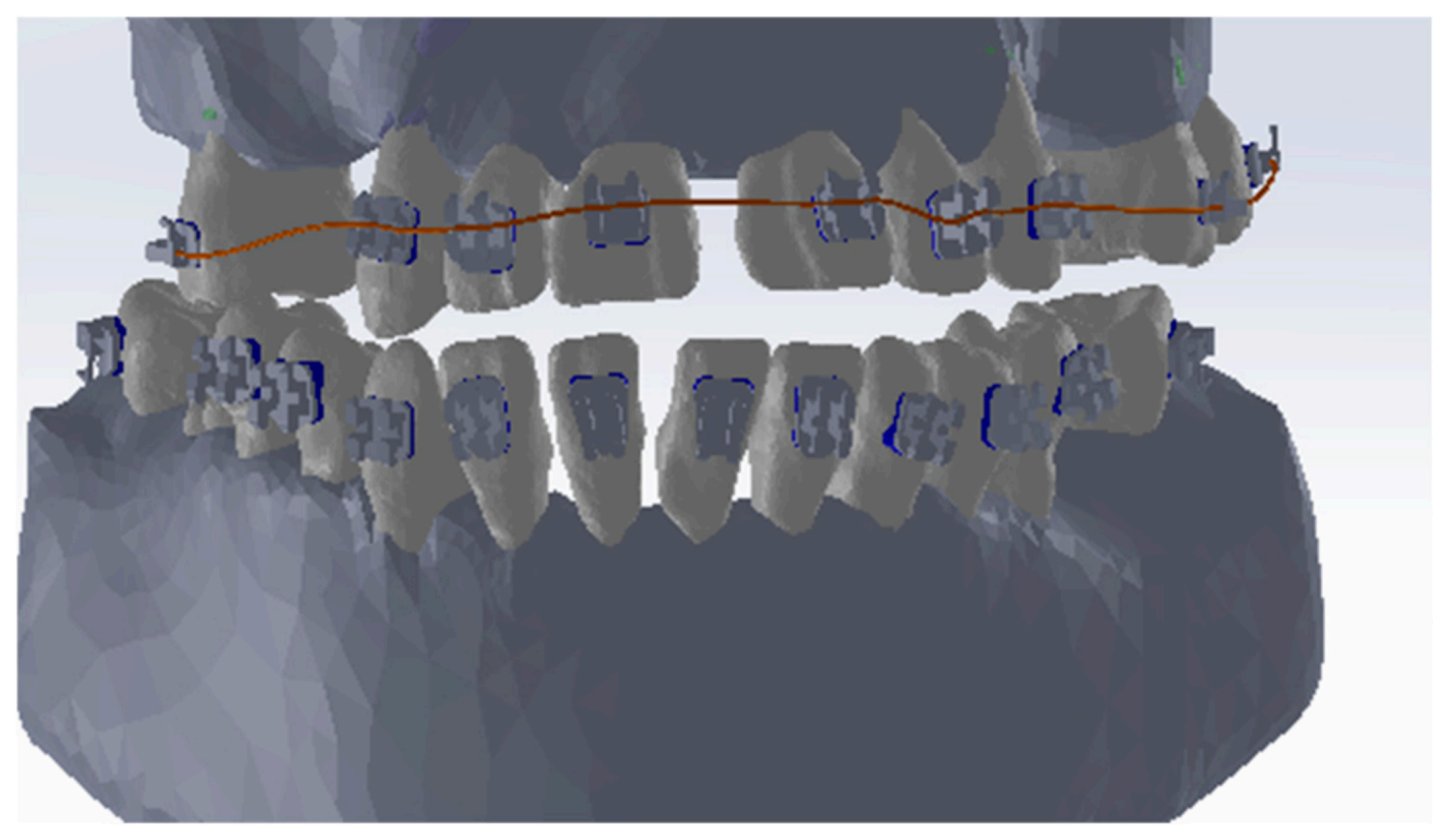
Model of the upper orthodontic wire.

Figure 51 shows the model of the orthodontic wire for the maxilla.

**Figure 52:**
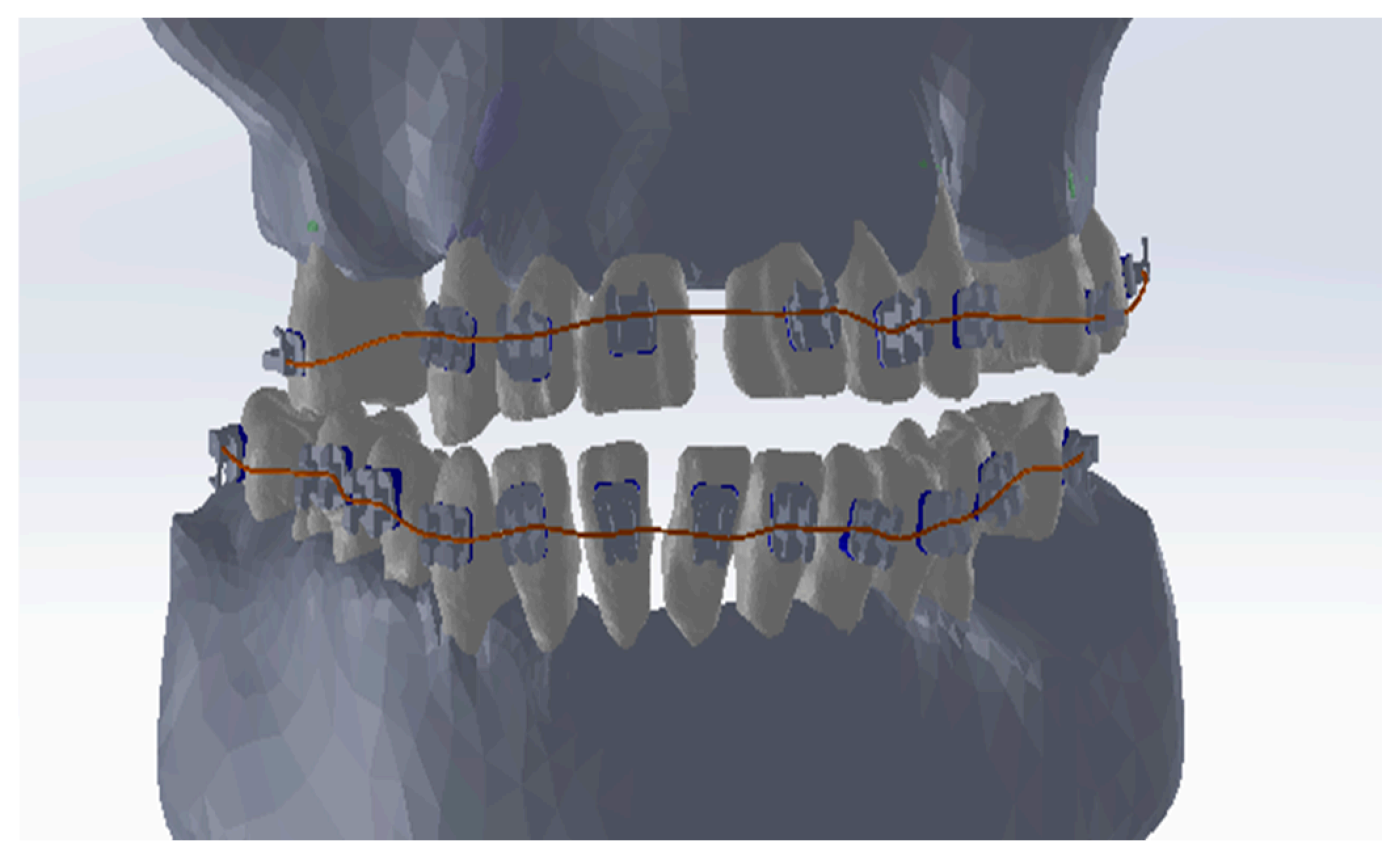
Model of the orthodontic archwires.

Similarly, the orthodontic wire for the mandible was also modelled (Figure 52).

#### 2.6.7. Role of the Three-Dimensional Reconstruction in the Mechanical Analysis

The three-dimensional reconstruction was used to establish a patient-specific geometric representation of the dento-maxillary system and to determine the spatial configuration of the orthodontic appliance. The reconstructed bone, teeth, periodontal ligament, adhesive, and bracket components were used primarily to define the anatomical environment and the spatial positions of the bracket-related reference points.

It should be emphasized that, in the present study, these anatomical components were not mechanically coupled through constitutive material models to the subsequent analytical force calculation. Their influence on the calculated results was indirect and occurred through the geometric chain by which the patient-specific anatomy determined tooth position, bracket location, and the resulting orthodontic archwire geometry.

The final analytical calculation was therefore based on geometric parameters extracted from the reconstructed archwire together with the elastic properties and cross-sectional geometry of the nickel–titanium wire.

### 2.7. Application of a Local Equivalent Beam Model for Estimating Orthodontic Archwire Forces Introductory Theoretical Concepts

**Figure 53:**
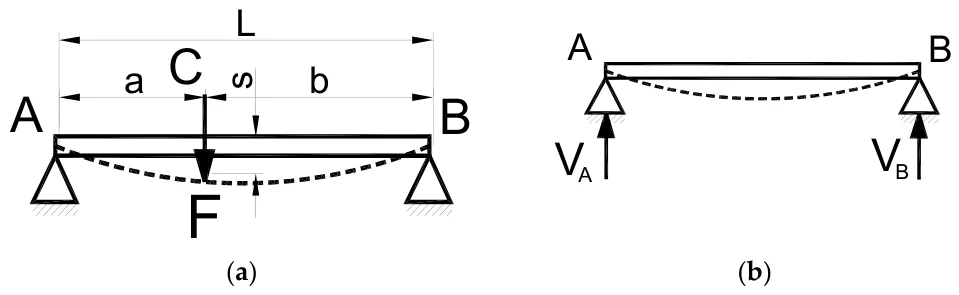
The elastic force F that produces the deflection s ((**a**) model with action forces; (**b**) equivalent model with reaction forces).

From the Strength of Materials and the Theory of Elasticity, it is known that a bar with a constant cross-section, supported at both ends and subjected to a force F, undergoes an elastic deformation s. This deformation is called deflection (Figure 53a). Figure 53b shows the equivalent model based on reactions.

An orthodontic archwire can be considered a bar with a constant cross-section. Furthermore, points A, B, and C can correspond to bracket-type components. We can thus map the model in Figure 52 onto the bracket-type elements on the orthodontic archwire. Now, the problem is to determine the elastic forces F and the reactions V_A_ and V_B_.

It is also known that there is a functional relationship between the force F and the deflection s. This is

(1) F=k · s

where

F—elastic force;

k—elastic constant;

s—elastic deformation (deflection).

From the Principles of Strength of Materials and the Theory of Elasticity, the elastic force F can be calculated as follows:

(2) $$F=\frac{9\;\sqrt{3}\cdot L\cdot E\cdot I\cdot s}{a\cdot {\left(L^{2}-a^{2}\right)}^{3/2}}$$

where s is the deflection of the bar;

F is the elastic force;

L is the length of the bar;

E is Young’s modulus (the modulus of elasticity of the orthodontic archwire);

I is the axial moment of inertia, and, in the case of a bar with a circular cross-section, it is where d is the diameter of the bar (in this case, the diameter of the orthodontic archwire).

(3) $$I=\frac{\pi \cdot d^{4}}{64}$$

From Equations (1) and (2), the elastic constant k can be determined as follows:

(4) $$k=\frac{9\;\sqrt{3}\cdot L\cdot E\cdot I}{a\cdot {\left(L^{2}-a^{2}\right)}^{3/2}}$$

If the elastic force F is known, the reactions V_A_ and V_B_ can be estimated as follows:

(5) $$V_{A}\;=\frac{F\cdot b}{L}$$

(6) $$V_{B}\;=\frac{F\cdot a}{L}\;și\;V\;=V_{A}\;+\;V_{B}$$

Table 1 shows how mathematical models are applied to the jaw.

Similarly, the mathematical models were adapted for the mandibular dentition, as shown in Table 2.

where

M_1dr_—right primary molar;

M_2dr_—right secondary molar;

P_1dr_—right primary premolar;

P_2dr_—right secondary premolar;

C_dr_—right canine;

I_2dr_—right secondary incisor;

I_1dr_—right primary incisor;

M1st—left primary molar;

M2st—left second molar;

P1st—left first premolar;

P2st—left second premolar;

C_st_—left canine;

I2st—left second incisor;

I1st—left first incisor.

**Figure 54:**
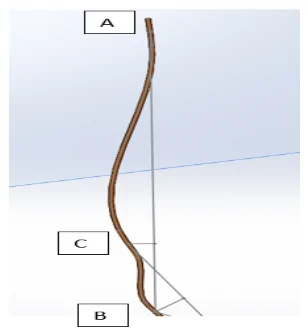
Drawing the lines AB and the perpendiculars from C.

To determine the lengths L, a, b, and s, points of type A and B were connected by line segments; then, from points of type C, perpendiculars were drawn to the lines of type AB to highlight the arrows of type **s**, as illustrated for the orthodontic wire on the maxilla in Figure 54. Point A corresponds to M_1dr_, B corresponds to C_dr_, and point C corresponds to I_2dr_.

**Figure 55:**
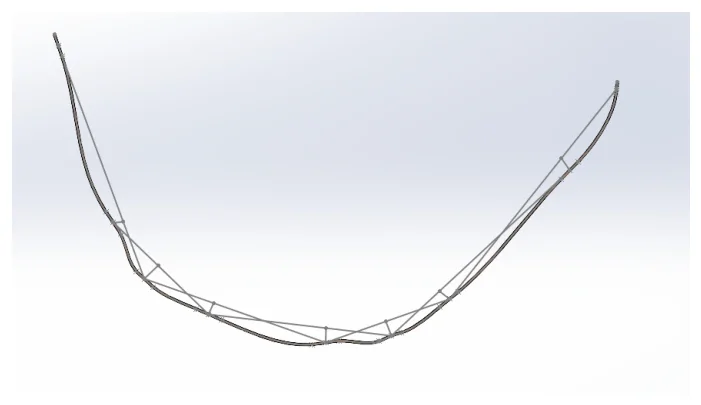
Drawing the lines AB and the perpendicular from C for the upper orthodontic wire.

For the maxillary orthodontic wire, the lines are shown in Figure 55.

**Figure 56:**
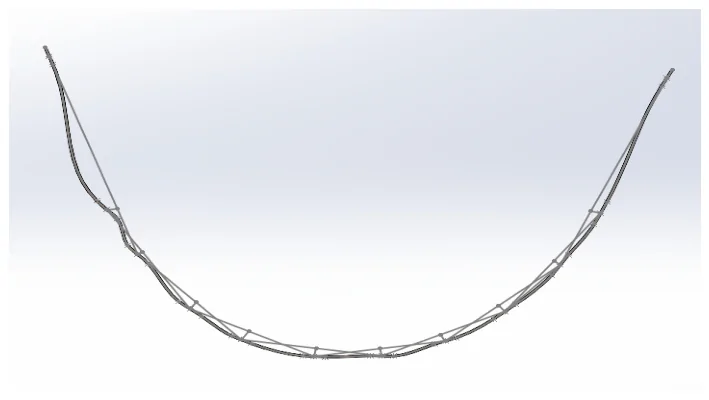
Drawing the lines AB and the perpendicular from C for the lower orthodontic wire.

For the mandibular orthodontic wire, these lines are shown in Figure 56.

**Figure 57:**
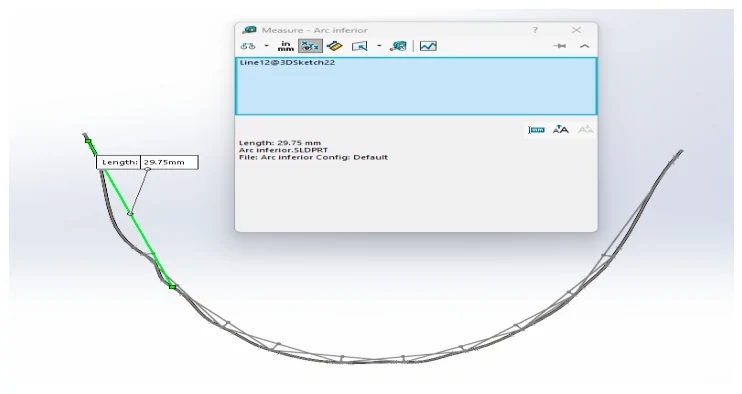
Measuring the distance AB.

Figure 57 illustrates how to measure the L-type length, that is, segment AB (M_1dr_ − I_2dr_).

**Figure 58:**
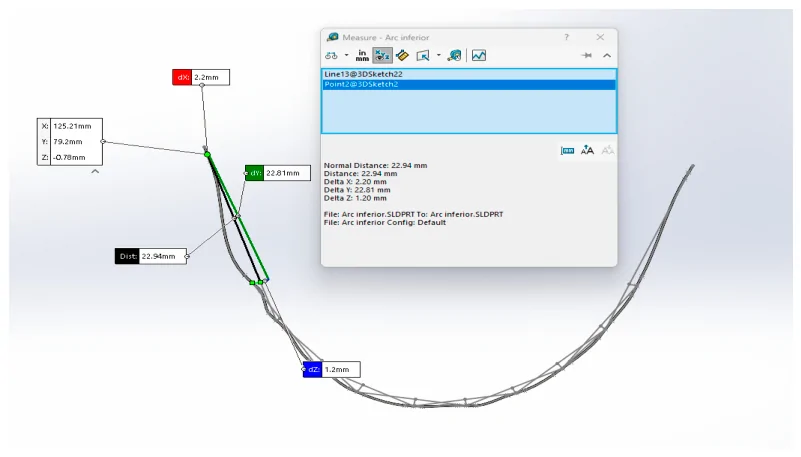
Measurement of distance type a.

Figure 58 illustrates how to measure a length of type a, that is, the distance from point A (M_1dr_) to the foot of the perpendicular drawn from point C (C_dr_).

**Figure 59:**
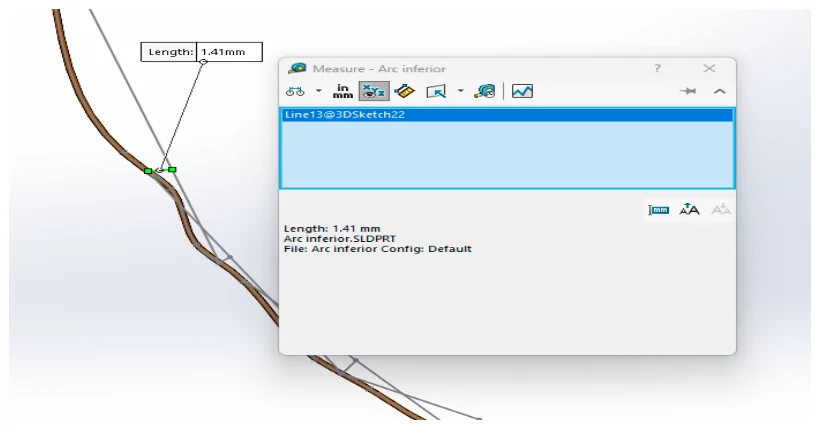
Measurement of the s-type distance.

To determine distances of type s, the length of the perpendicular drawn from point C (C_dr_) to line AB (M_1dr_ − I_2dr_) was measured, as shown in Figure 59.

b-type distances are calculated using the formula

b = L − a

#### 2.7.1. Mechanical Idealization

The mechanical analysis performed in the present study was based on a local equivalent beam approximation derived from the reconstructed patient-specific geometry of the orthodontic archwire. The purpose of this formulation was not to reproduce the complete three-dimensional mechanical behavior of the orthodontic appliance, but to estimate geometry-dependent local elastic force components using a simplified and analytically tractable model.

For each local configuration, three bracket-related reference points were considered. Points A and B defined two neighboring reference locations, while point C represented an intermediate bracket-related point. The straight segment connecting A and B was used as a local reference chord.

The corresponding portion of the orthodontic wire was represented by an equivalent prismatic beam with a constant circular cross-section. The analytical model assumed linear-elastic material behavior, a constant Young’s modulus, small local deflections, and negligible shear deformation. Accordingly, the classical Euler–Bernoulli beam formulation was adopted.

It should be emphasized that this idealization does not reproduce all mechanical interactions occurring in a clinical bracket–archwire system. In particular, bracket rotational stiffness, torsion, out-of-plane bending, friction, nonlinear contact, and the complete three-dimensional transmission of moments were not explicitly included.

#### 2.7.2. Definition of the Local Geometric Parameters

For each local analytical configuration, the distance between points A and B was defined as L = AB.

The position of point C was characterized by its projection onto the local chord AB. The distance between point A and the projection of C onto AB was defined as (a), whereas the remaining distance was

b = L − a(7)

The perpendicular distance between point C and the reference chord AB was defined as (s).

The parameter (s) was used as an equivalent local geometric activation parameter in the analytical model. It should not be interpreted as a directly measured in vivo deformation of the orthodontic wire. Instead, it represents the local geometric offset of the reconstructed wire configuration relative to the idealized reference chord AB.

#### 2.7.3. Equivalent Elastic Force

The local three-point configuration was analyzed using the classical Euler–Bernoulli solution for a simply supported prismatic beam subjected to a concentrated transverse force (F) applied at point C.

For this loading configuration, the transverse deflection evaluated at the load application point C is

(8) $$s=\frac{F\cdot a\cdot {\left(L^{2}-a^{2}\right)}^{3/2}\;}{9\;\sqrt{3}\cdot L\cdot E\cdot I}$$

where

- (s) is the transverse deflection evaluated at point C;
- (F) is the equivalent concentrated transverse force;
- (L) is the distance between the idealized supports A and B;
- and (b) are the distances from C to A and B, respectively;
- (E) is Young’s modulus of the orthodontic wire;
- is the second moment of area of the circular cross-section.

and where (E) is Young’s modulus of the orthodontic wire and (I) is the second moment of area of its cross-section.

Therefore, the equivalent elastic force associated with the local geometric activation is

(9) $$F=\frac{9\;\sqrt{3}\cdot L\cdot E\cdot I\cdot s}{a\cdot {\left(L^{2}-a^{2}\right)}^{3/2}}$$

For the circular cross-section of the orthodontic wire,

(10) $$I=\frac{\pi \cdot d^{4}}{64}$$

where (d) is the wire diameter.

The calculated force should be interpreted as an equivalent local elastic force associated with the measured geometric activation under the assumptions of the simplified beam model.

#### 2.7.4. Local Reaction Components

For each local equivalent beam configuration, the reactions at the two reference points were obtained from static equilibrium:

(11) $$V_{A}\;=\;\frac{F\cdot b}{L}$$

and

(12) $$V_{B\;=}\frac{F\cdot a}{L}$$

These quantities represent local transverse reaction components within the corresponding analytical configuration.

When a bracket-related point participated in two neighboring local configurations, the contributions obtained from each configuration were considered according to the analytical assembly procedure adopted in the present model. The resulting reported quantity represents an equivalent local transverse reaction component.

The reported reaction values do not represent a complete three-dimensional vector resultant and do not include moments transmitted through the bracket–archwire interaction.

#### 2.7.5. Range of Validity

The present analytical approach is intended as a reduced-order mechanical approximation for evaluating the influence of patient-specific orthodontic archwire geometry on local elastic force estimates.

The model is based on the following assumptions:

- Constant circular wire cross-section;
- Homogeneous material properties;
- Linear-elastic behavior;
- Small local deflections;
- Local Euler–Bernoulli beam behavior;
- Idealized support conditions;
- No explicit bracket rotational stiffness;
- No torsional response;
- No explicit out-of-plane bending;
- No friction or nonlinear bracket–archwire contact;
- No coupled constitutive response of the periodontal ligament, teeth, bone, or adhesive.

Consequently, the calculated quantities should not be interpreted as the complete clinical force–moment system acting on the orthodontic appliance. A continuous three-dimensional beam model or a finite element analysis incorporating actual bracket geometry, contact conditions, and moment transfer would be required for a more comprehensive biomechanical representation.

**Figure 60:**
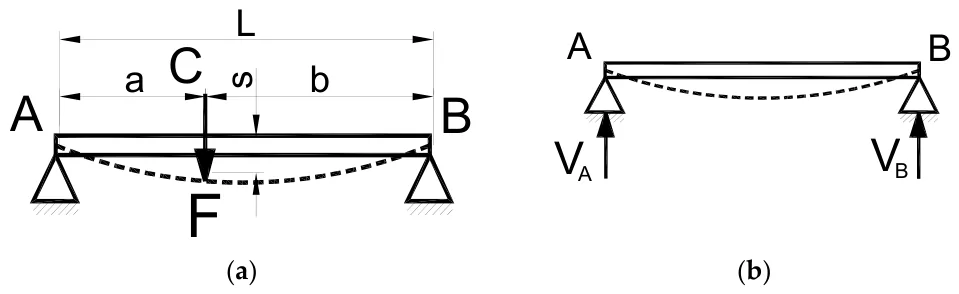
The elastic force F that produces the deflection s ((**a**) model with action forces; (**b**) equivalent model with reaction forces).

From the Strength of Materials and the Theory of Elasticity, it is known that a bar with a constant cross-section, supported at both ends and subjected to a force F, undergoes an elastic deformation s. This deformation is called deflection (Figure 60a). Figure 60b shows the equivalent model based on reactions.

Table 3 shows how mathematical models are applied to the jaw.

Similarly, the mathematical models were adapted for the mandibular dentition, as shown in Table 4.

where

M_1dr_—right primary molar;

M_2dr_—right secondary molar;

P_1dr_—right primary premolar;

P_2dr_—right secondary premolar;

C_dr_—right canine;

I_2dr_—right secondary incisor;

I_1dr_—right primary incisor;

M1st—left primary molar;

M2st—left second molar;

P1st—left first premolar;

P2st—left second premolar;

C_st_—left canine;

I2st—left second incisor;

I1st—left first incisor.

**Figure 61:**
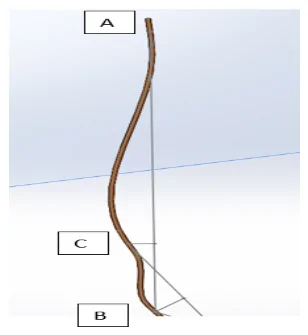
Drawing the lines AB and the perpendiculars from C.

To determine the lengths L, a, b, and s, points of type A and B were connected by line segments; then, from points of type C, perpendiculars were drawn to the lines of type AB to highlight the arrows of type s, as illustrated for the orthodontic wire on the maxilla in Figure 61. Point A corresponds to M_1dr_, B corresponds to C_dr_, and point C corresponds to I_2dr_.

**Figure 62:**
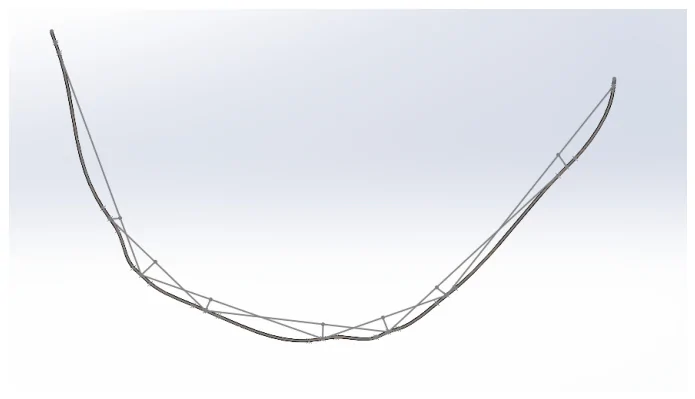
Drawing the lines AB and the perpendicular from C for the upper orthodontic wire.

For the maxillary orthodontic wire, the lines are shown in Figure 62.

**Figure 63:**
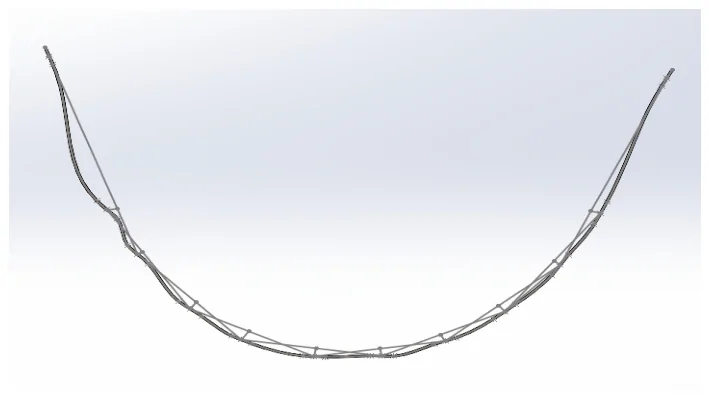
Drawing the lines AB and the perpendicular from C for the lower orthodontic wire.

For the mandibular orthodontic wire, these lines are shown in Figure 63.

**Figure 64:**
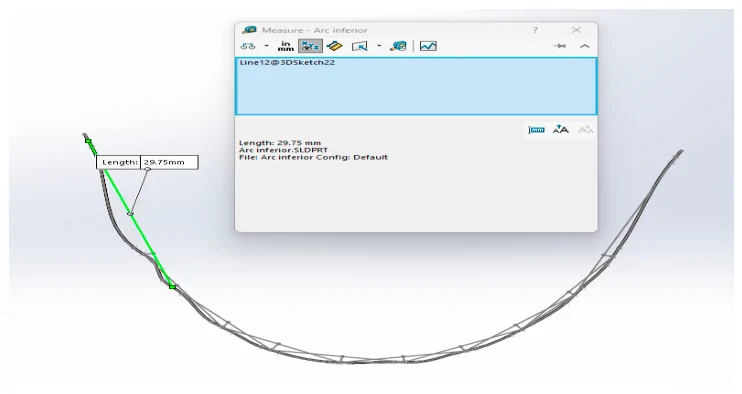
Measuring the distance AB.

Figure 64 illustrates how to measure the L-type length, that is, segment AB (M_1dr_ − I_2dr_).

**Figure 65:**
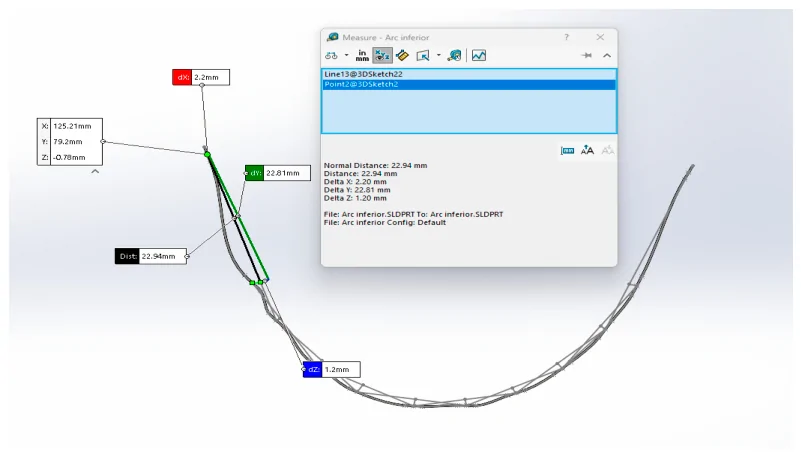
Measurement of distance type a.

Figure 65 illustrates how to measure a length of type a, that is, the distance from point A (M_1dr_) to the foot of the perpendicular drawn from point C (C_dr_).

**Figure 66:**
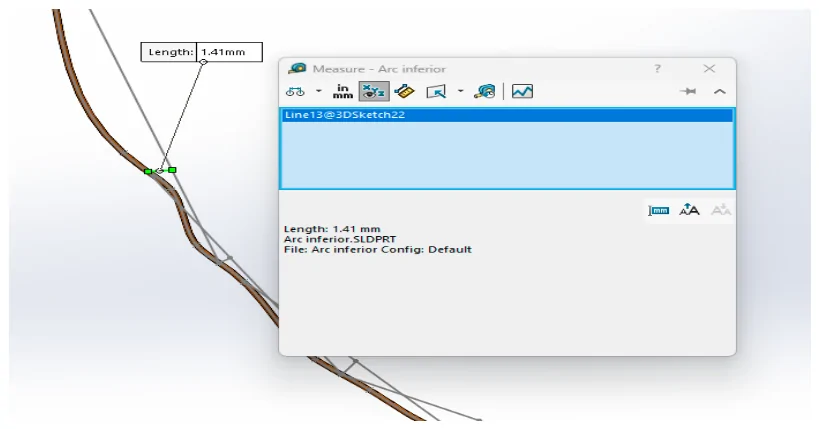
Measurement of the s-type distance.

To determine distances of type s, the length of the perpendicular drawn from point C (C_dr_) to line AB (M_1dr_ − I_2dr_) was measured, as shown in Figure 66.

To determine the geometric parameters of each local equivalent beam configuration, the bracket-related reference points A and B were connected by a straight line defining the local reference chord. The perpendicular projection of point C onto this chord was then determined. The distance AB defined the local span L, the distance between A and the projection of C defined a, and the perpendicular offset between C and AB defined the equivalent local geometric activation parameter s. The remaining distance was calculated as

b = L − a

### 2.8. Three-Dimensional Finite Element Verification of the Analytical Model

**Figure 67:**
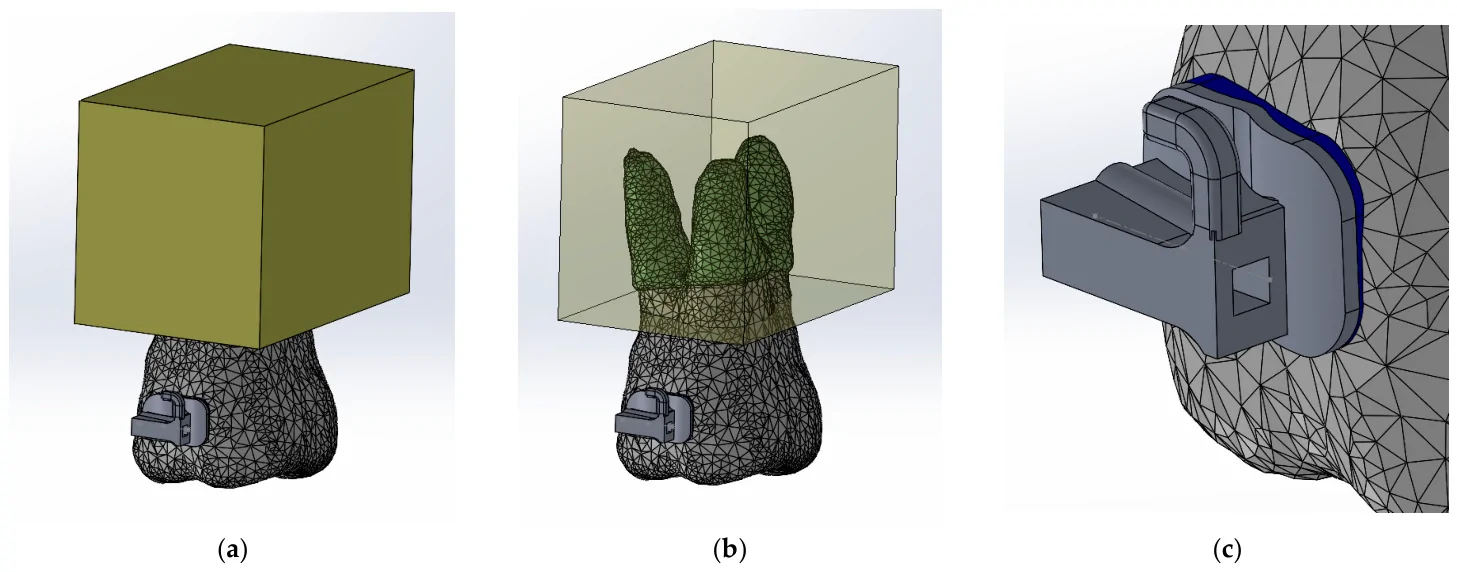
A model of a molar ((**a**) complete model, (**b**) model with a transparent bone fragment, (**c**) placement of the blue-colored adhesive).

**Figure 68:**
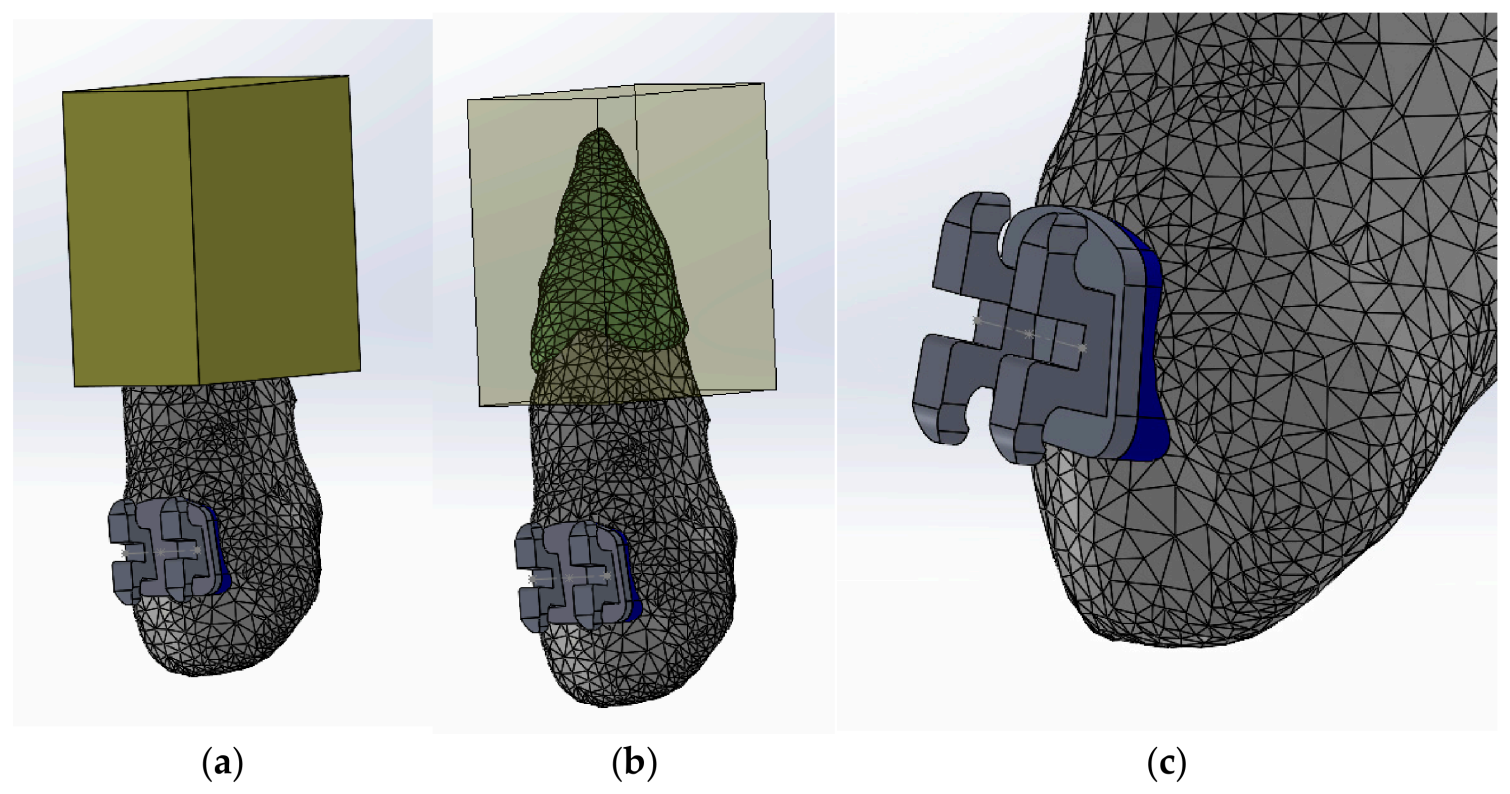
A model of a canine tooth ((**a**) complete model, (**b**) model with a transparent bone fragment, (**c**) location of the blue adhesive).

**Figure 69:**
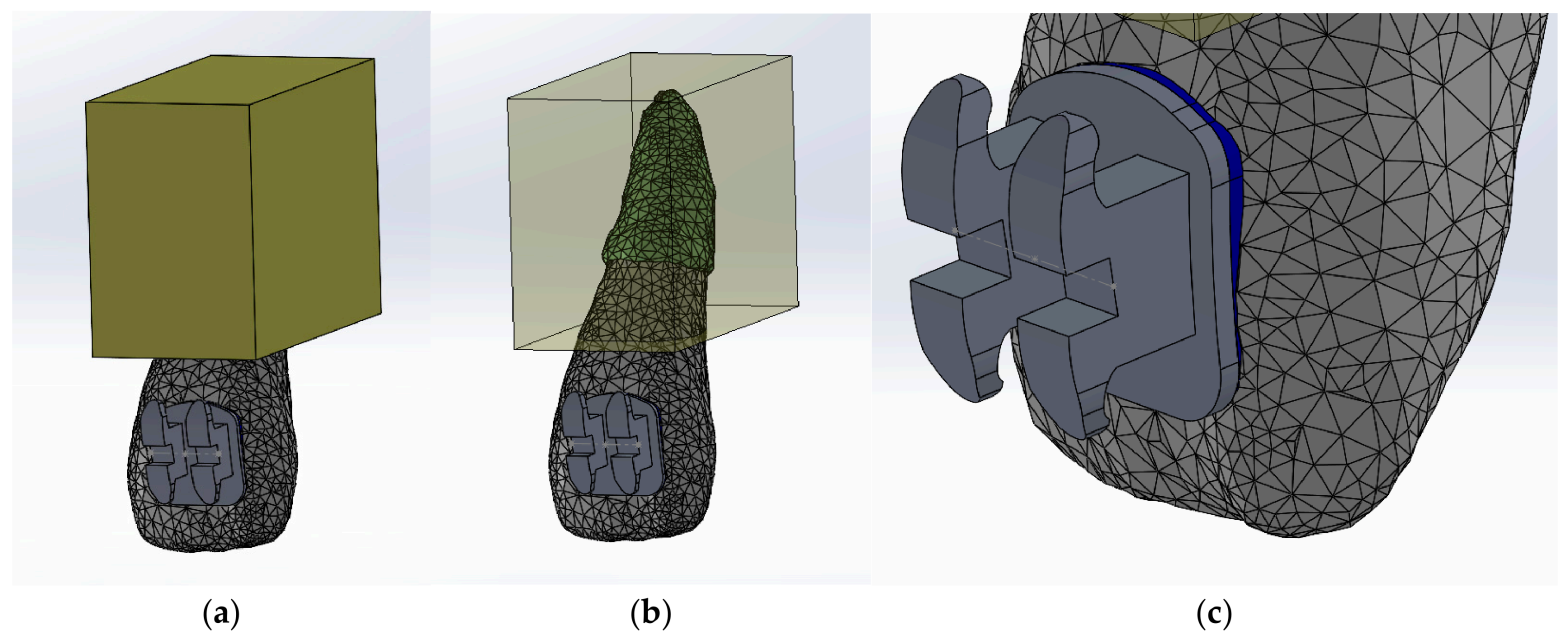
A model of an incisor ((**a**) complete model, (**b**) model with a transparent bone fragment, (**c**) placement of the blue-colored adhesive).

To verify the analytical results, three assemblies were created in SolidWorks, each consisting of a tooth model and a ligament inserted into a bone fragment; the adhesive model and the bracket-type component were placed on the tooth surface. The bracket-type elements were connected by an orthodontic archwire. Figure 67 shows the assembly corresponding to a molar, Figure 68 shows the assembly corresponding to a canine, and Figure 69 shows the assembly corresponding to an incisor.

**Figure 70:**
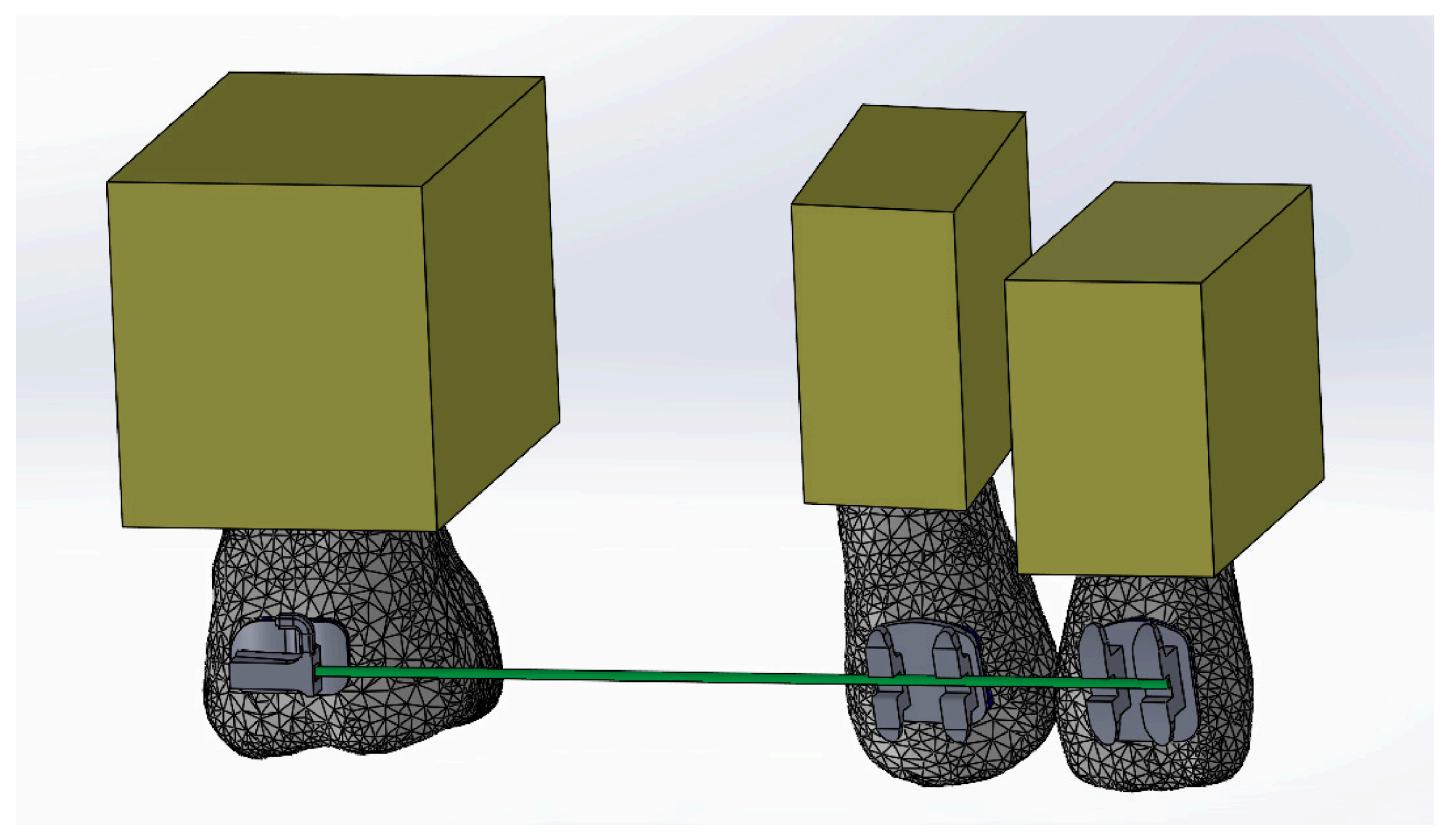
The Three-Tooth Model.

These assemblies were imported into a larger assembly and positioned at the distances measured on the quasi-real model derived from the patient’s CBCT (L, a, b). Within this assembly, a model of an orthodontic archwire was created to connect the bracket-type components (shown in green in Figure 70).

**Figure 71:**
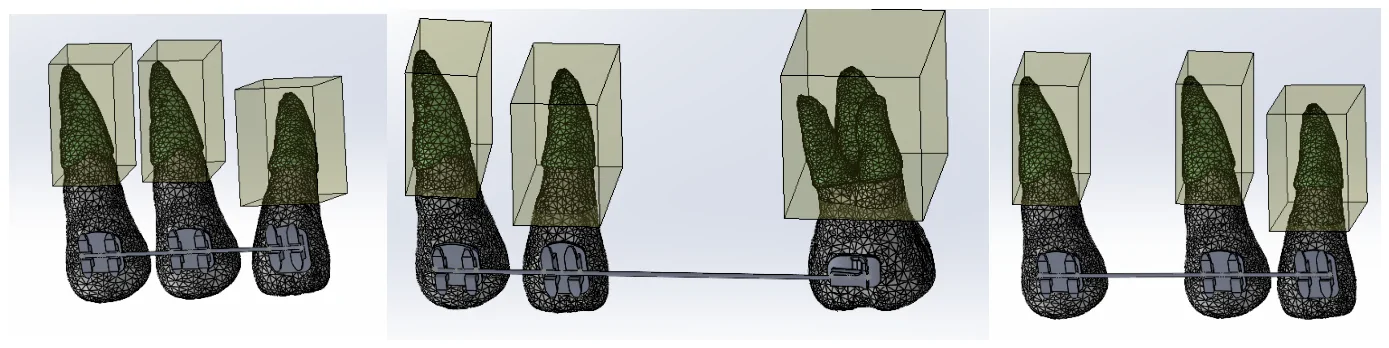
Three virtual assemblies corresponding to the upper arch.

**Figure 72:**
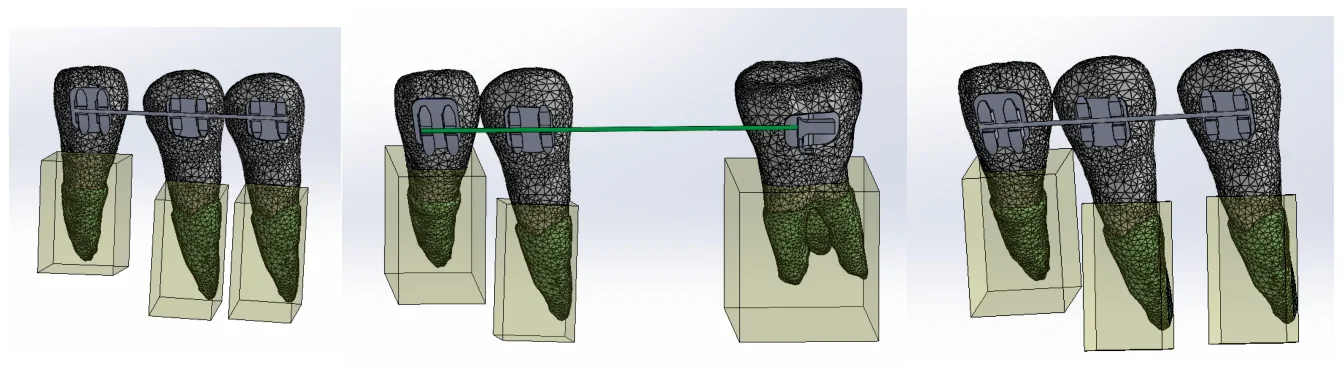
Three virtual assemblies corresponding to the lower arch.

Assemblies were created for all scenarios involving the upper dental arch, as well as for the lower dental arch. Figure 71 shows three assemblies corresponding to the maxillary dental arch, and Figure 72 presents three virtual assemblies corresponding to the mandibular dental arch.

**Figure 73:**
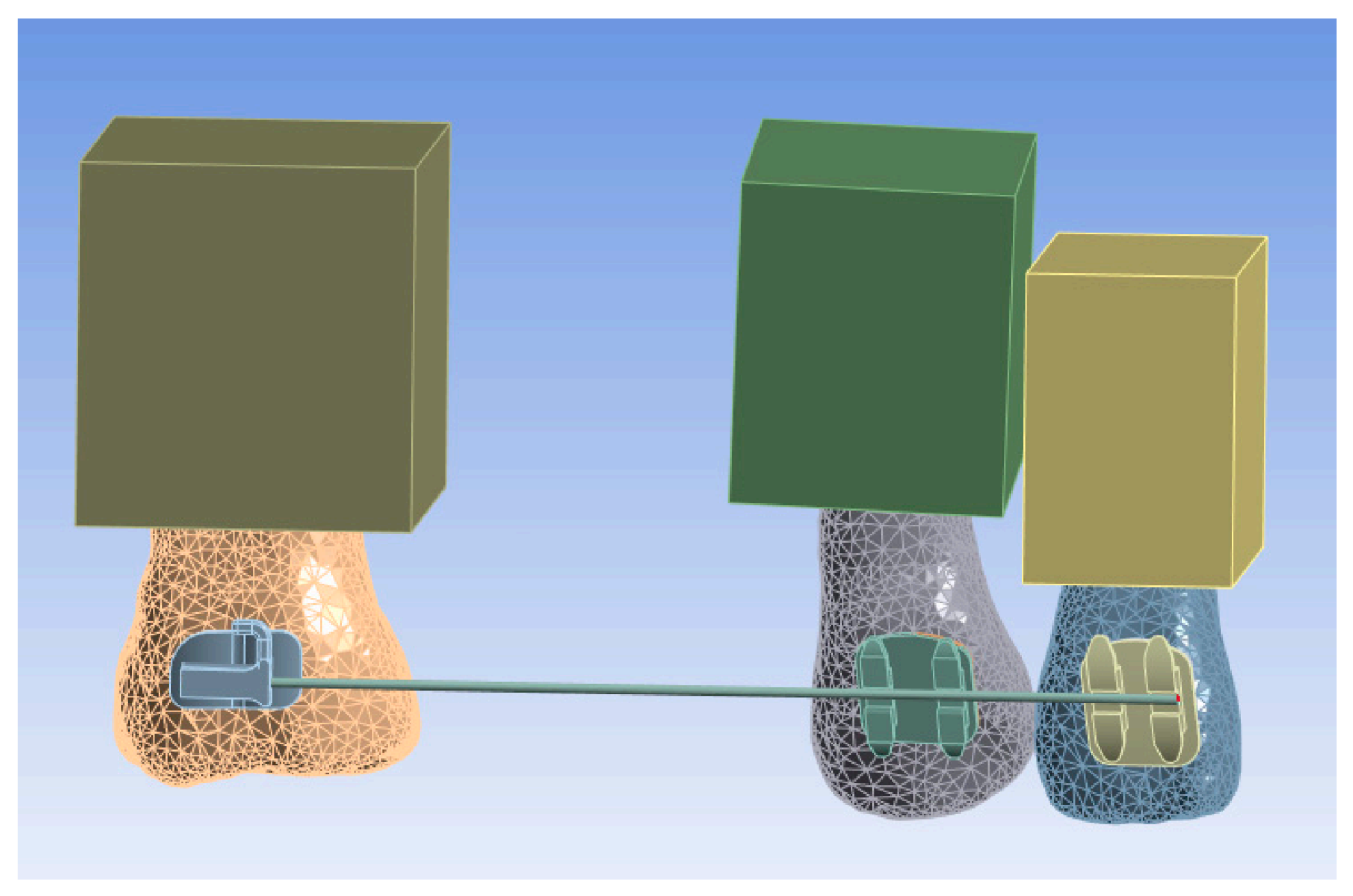
The model imported into Ansys.

The corresponding model for the molar, canine, and incisor on the maxillary arch was imported into Ansys Workbench (Figure 73).

In the first phase, material properties were assigned to the components in the assembly, as shown in Table 5. Furthermore, the interaction between the orthodontic archwire and the bracket was assumed to be frictional, with a coefficient of friction of 0.01.

**Figure 74:**
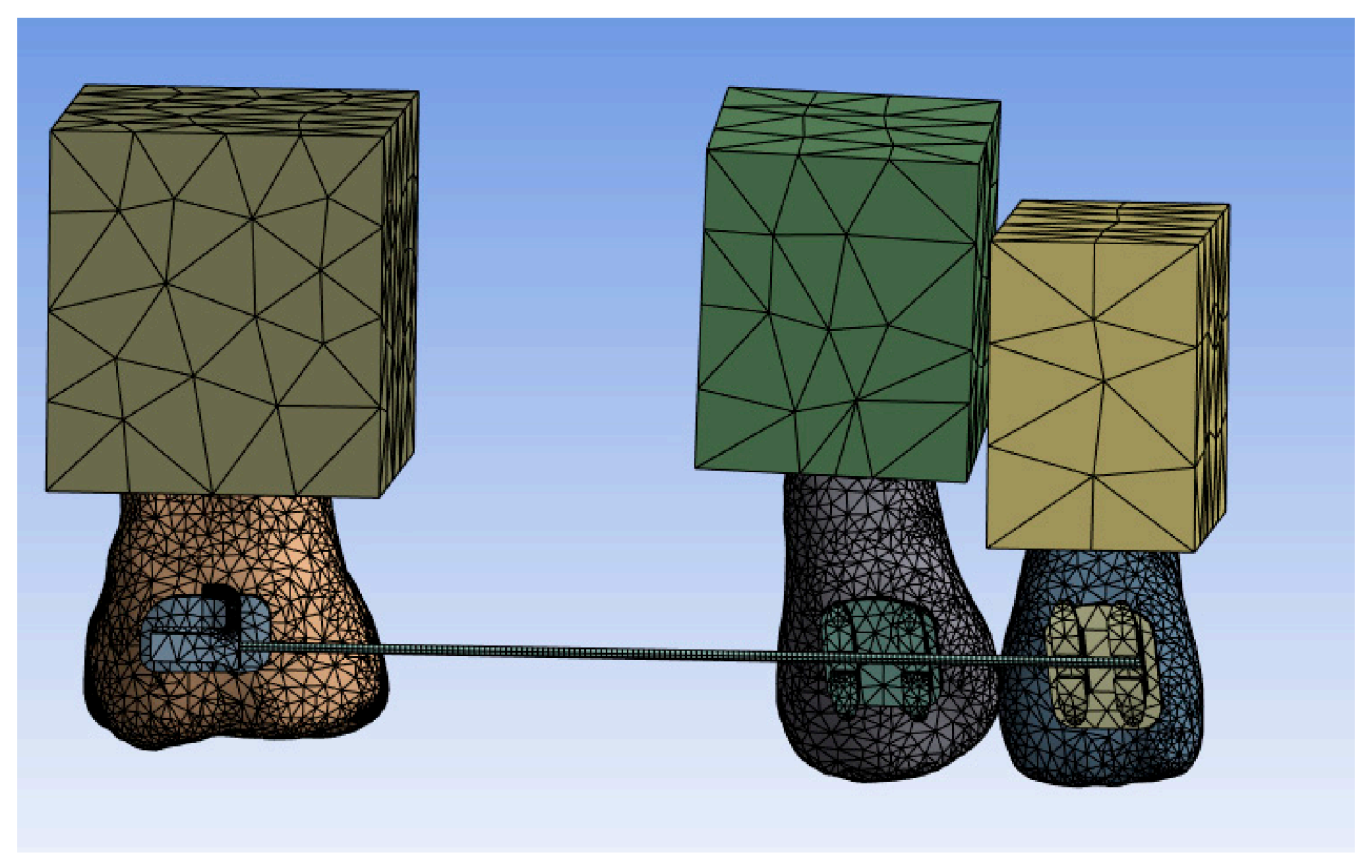
Finite element structure.

In the next stage, the assembly was divided into finite elements, resulting in a structure comprising 157,403 nodes and 75,108 elements, as shown in Figure 74.

**Figure 75:**
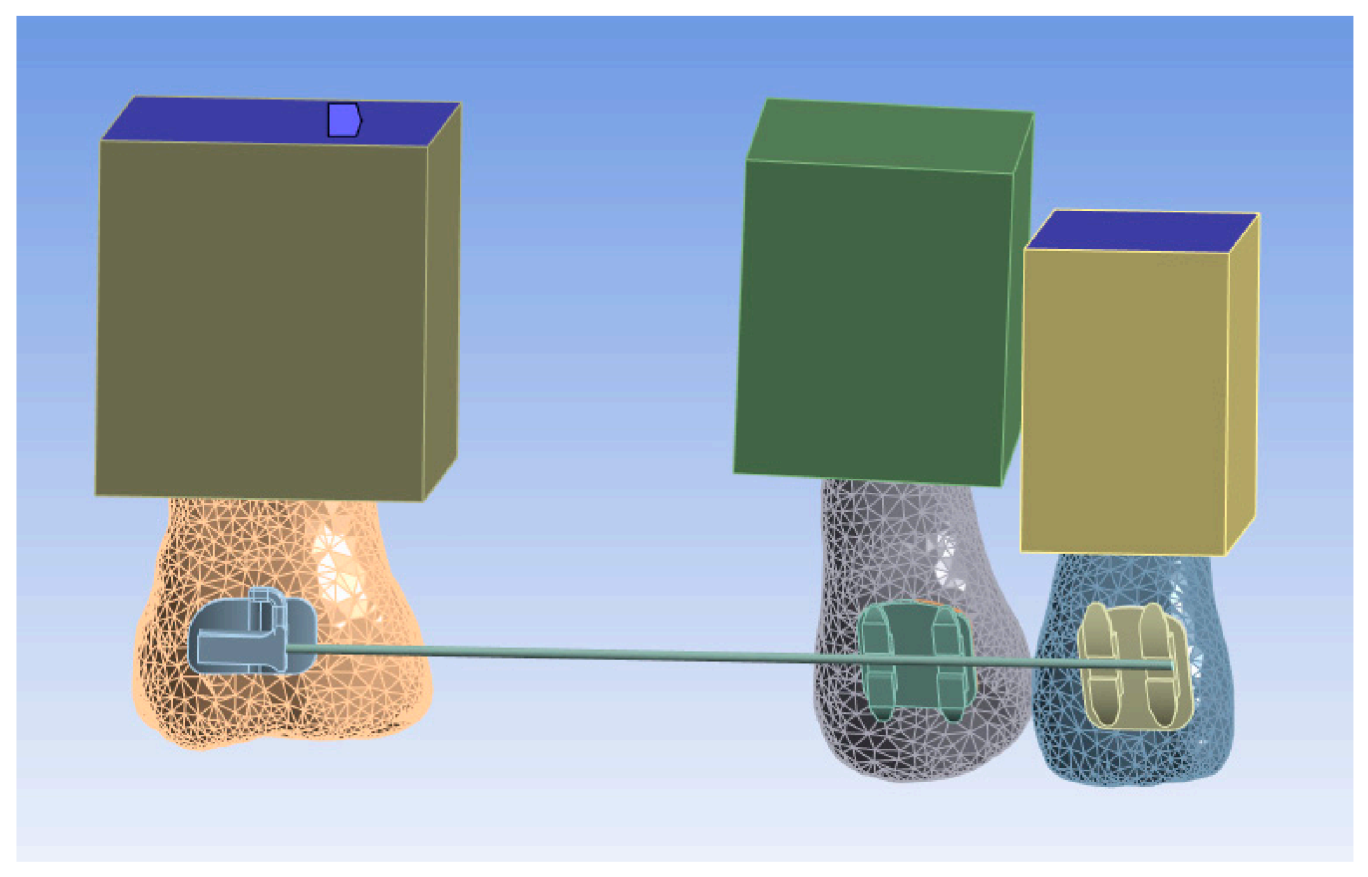
Fixed surfaces.

The upper surfaces of the bony components of the marginal teeth (molar and incisor) were considered to be fixed, as can be seen in Figure 75, where they are coloured in shades of blue.

**Figure 76:**
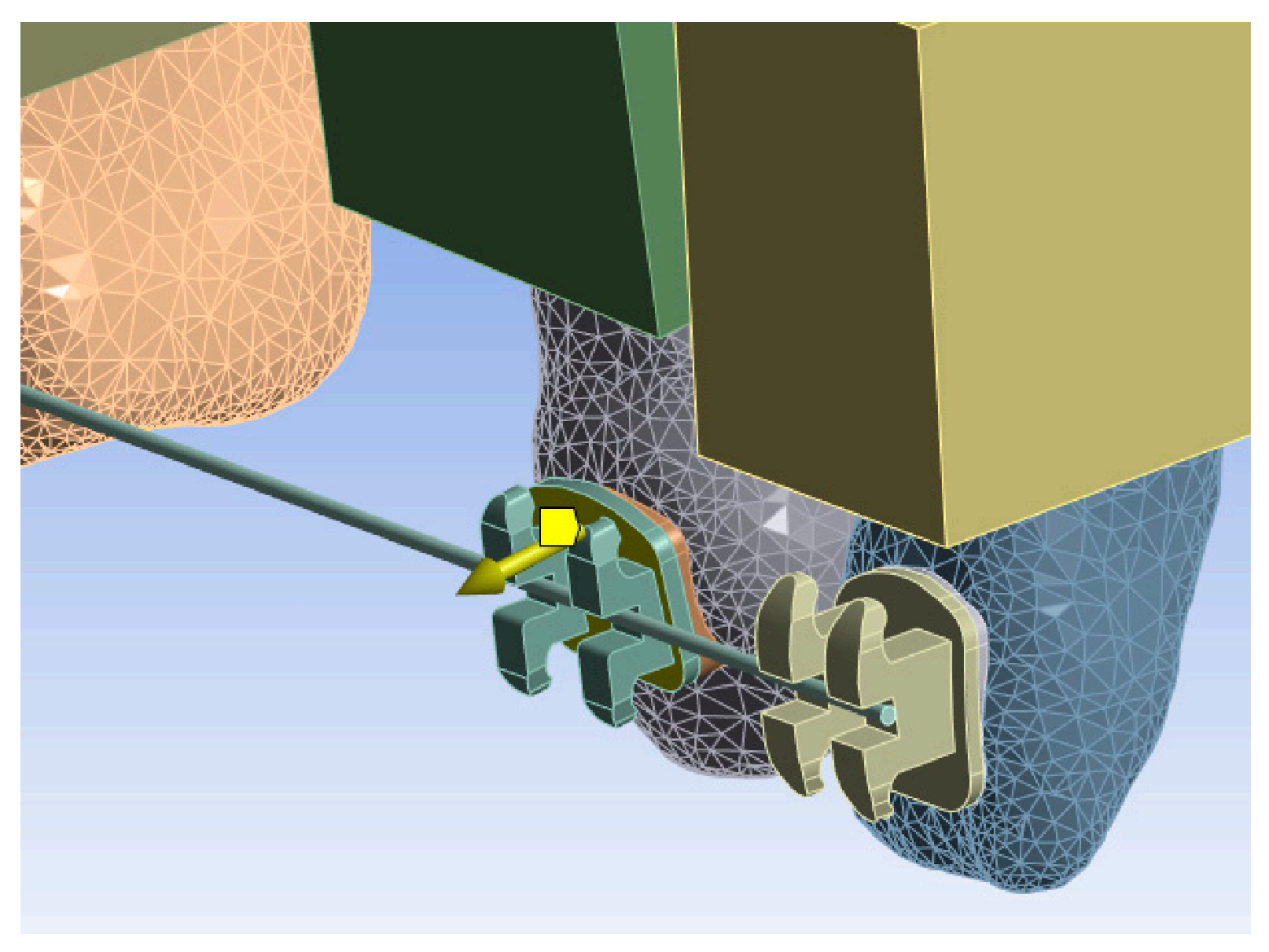
Displacement constraint.

A displacement constraint with a value of 1.41 mm was applied to the surface of the median bracket for this scenario; this is equal to the elastic deformation s, measured on the model derived from the CBCT, as can be seen in Figure 76.

With the ‘Nodal Forces’ simulation parameter set to ‘On’, the reactions in the bracket-type elements were determined at the interface between the metal and the orthodontic adhesive.

## 3. Results

### 3.1. Determination of the Geometric Parameters of the Local Equivalent Beam Configurations

The patient-specific three-dimensional reconstruction was used to determine the geometric parameters required by the local equivalent beam model. For each local configuration, the span L, the load-position parameter a, the complementary distance b, and the equivalent local geometric activation parameter s were extracted from the reconstructed orthodontic archwire geometry.

For the maxilla, the distances measured on the orthodontic wire curve are shown in Table 6, and for the mandible, they are shown in Table 7.

### 3.2. Determination of the Elastic and Reaction Forces Acting on Bracket-Type Components

Using the geometric parameters extracted from the patient-specific reconstruction, together with the diameter and elastic modulus assigned to the nickel–titanium wire, the equivalent local elastic forces and transverse reaction components were calculated according to the analytical relationships described in Section 2.7.

The diameter of the spring is 0.012 inches, or 0.3048 mm, so

d = 0.0003048 m

The spring is made of a nickel–titanium alloy, known as nitinol. For this material, the modulus of elasticity is

E = 34,500,000,000 Pa

By applying the mathematical formulas that were incorporated into Microsoft Excel, the specific forces of the maxilla presented in Table 8 and those of the mandible in Table 9 were determined.

**Figure 77:**
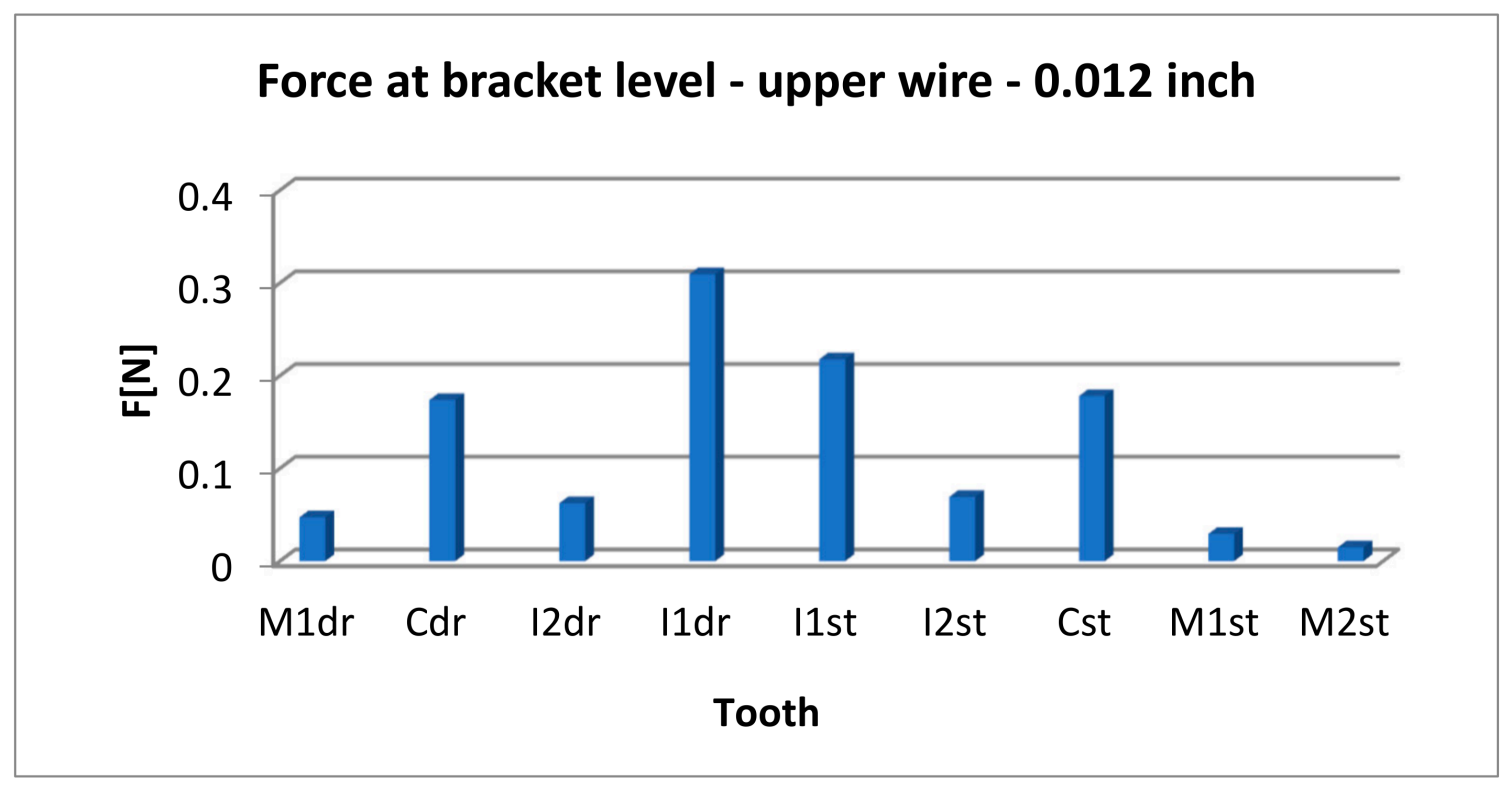
Forces acting on the upper wire.

**Figure 78:**
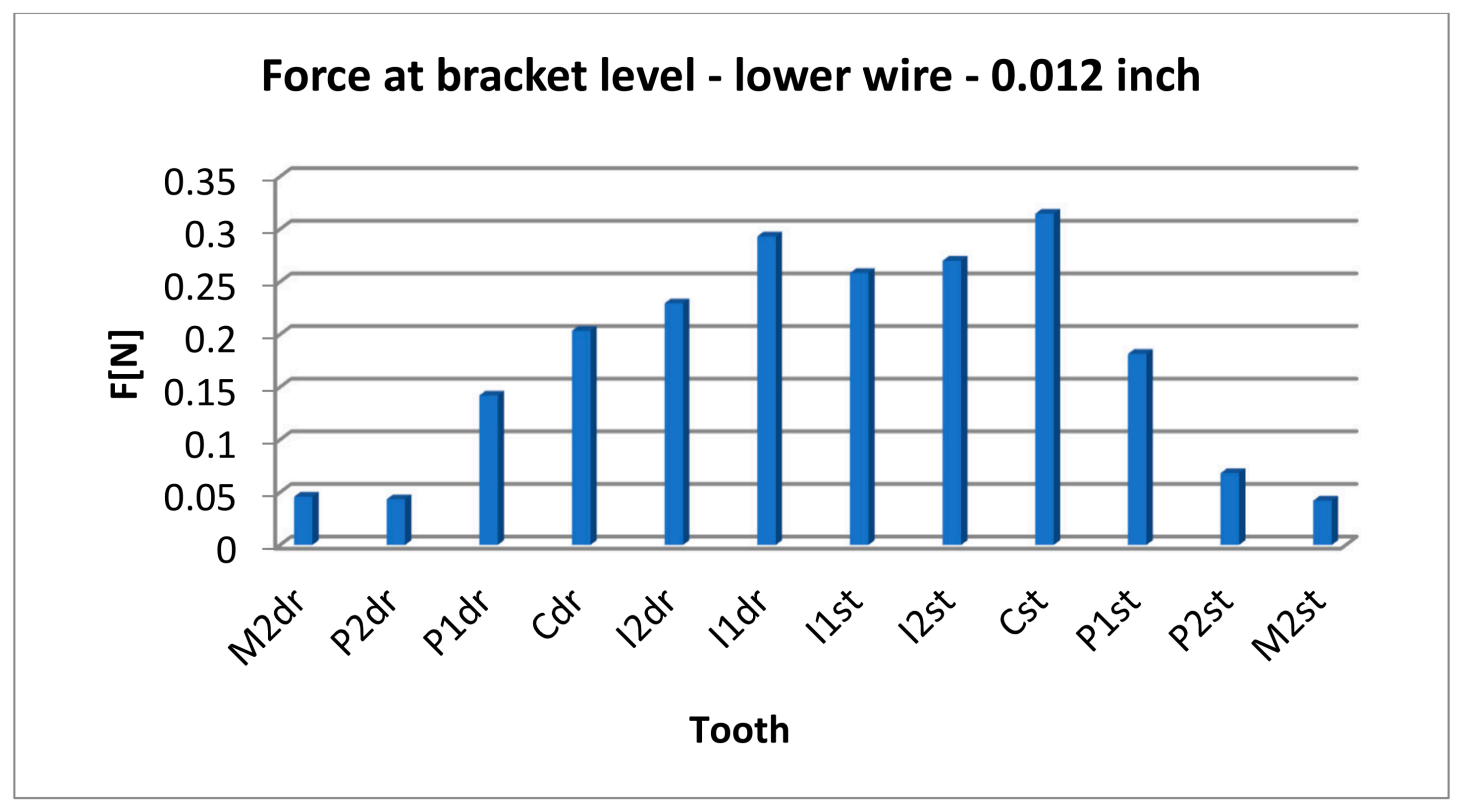
Forces acting on the lower wire.

These values are summarized in the graphs in Figure 77 and Figure 78.

### 3.3. Numerical Verification of the Analytical Force Model

The results of the simulations were organized into tables of values, and the following formula was used to calculate the errors:

(13) $$Error\;\left[\%\right]=\frac{\left|V_{FEM-\;}V_{analytical}\right|\;\times \;100}{V_{analytical}}$$

V_FEM_—the reaction obtained through FEA simulation;
V_analytical_—the reaction obtained analytically.

Table 10 and Table 11 show the reaction values obtained from FEM simulations.

## 4. Discussion

This study aimed to evaluate the system of forces acting within an orthodontic appliance through an integrated engineering and medical approach, based on three-dimensional modeling, virtual measurements, and the application of classical methods from the Theory of Elasticity and Strength of Materials. The results obtained are consistent with the general direction described in the specialized literature, according to which orthodontic biomechanics must be analyzed by correlating the geometry of the appliance, the material properties, and the biological response of the dento-periodontal structures [26,27].

An essential element of the study is the construction of the three-dimensional geometric model, derived from CBCT images of a real patient.

In this regard, the results are consistent with the studies by Kapila et al., as well as those by Scarfe and Farman, which show that CBCT offers major advantages in orthodontics by providing precise three-dimensional information about dento-skeletal relationships [28,29]. However, unlike these studies, which focus primarily on diagnosis and planning, the present study extends the use of CBCT to a personalized mechanical analysis, in which imaging data are transformed into a numerical model capable of estimating force distribution.

The integration of bone, dental, and periodontal ligament models into a common coordinate system ensured the spatial consistency of the assembly and enabled the precise positioning of orthodontic components modeled using direct engineering. This step is critical from an engineering standpoint, as any misalignment would lead to erroneous values for the deformations and, consequently, for the calculated forces. Compared to the idealized models used in classical analysis of orthodontic systems, the present approach has the advantage of preserving the anatomical specificity of the case, which increases the clinical relevance of the results [30,31].

Modeling orthodontic wires using spline-type trajectories, defined based on the actual position of the brackets, represents a significant advantage of this study. This choice is consistent with the ideas formulated by Burstone, who demonstrated the importance of geometric control of the wire and the positioning of force application points in achieving predictable biomechanical systems [32]. The main difference from classical approaches lies in the fact that here the wire curves are not merely theoretically assumed but reconstructed from the actual position of the brackets, which reduces reliance on idealized assumptions and makes the analysis more closely aligned with the clinical situation.

From a mechanical standpoint, orthodontic wires have been considered elastic elements with a constant cross-section, and the material’s behavior has been approximated as linearly elastic. This assumption is useful for comparisons and for basic mechanical interpretation; however, the literature shows that modern Ni-Ti wires exhibit superelastic behavior and a significant dependence on activation and stress history [33]. Miura et al. [34] demonstrated, as early as in classical studies, that the Ni-Ti alloy possesses clinically relevant superelastic properties, and Kusy showed that the properties of orthodontic wires vary significantly depending on composition, diameter, and conditions of use [35].

An additional limitation concerns the mechanical idealization used to estimate the force components. Although the orthodontic appliance was reconstructed in three dimensions, the final analytical calculation was performed using local equivalent Euler–Bernoulli beam configurations. Therefore, the present approach does not represent the orthodontic archwire as a continuous three-dimensional structure and does not explicitly include torsion, out-of-plane bending, bracket rotational stiffness, nonlinear bracket–archwire contact, friction, or moment transfer. In addition, the reconstructed bone, teeth, periodontal ligament, and adhesive were used to establish the patient-specific geometry and the spatial position of the orthodontic components, but their constitutive behavior was not mechanically coupled to the analytical calculation. Consequently, the calculated values should be interpreted as equivalent local elastic force estimates associated with the reconstructed geometry rather than as a complete clinical force–moment system. Future work should compare the present analytical predictions with continuous three-dimensional beam models, finite element simulations, and experimentally measured orthodontic forces.

Therefore, the current model should be interpreted as a valid engineering approximation for comparative analysis, not as a complete description of the material’s actual behavior. Analysis of the results reveals a non-uniform distribution of forces along the orthodontic wires, in both the maxilla and the mandible. This result is consistent with the principles described by Burstone [32], according to which even small geometric changes can significantly alter the distribution of moments and forces. Furthermore, studies in orthodontic biomechanics emphasize that the efficiency of tooth movement depends not only on the magnitude of the force but also on its direction, duration, and point of application [36,37]. From this perspective, the non-uniform distribution observed in the present model confirms the sensitivity of the orthodontic system to the geometric configuration of the assembly.

It is important to note that the developed model does not include effects such as spring–bracket friction, nonlinear contact, the influence of the viscoelastic properties of periodontal ligaments, or biological adaptation over time. In the literature, these elements are recognized as important factors that can significantly alter the system’s actual response [38,39]. However, their simplification in the present study is justified by the need to obtain a clear, controllable, and interpretable model capable of highlighting the direct influence of geometry on the forces developed.

From a clinical perspective, the results are consistent with the classic observations of Reitan and Storey, who demonstrated that effective tooth movement is achieved through moderate, well-controlled forces, and that overloading can have undesirable biological effects [40]. In this regard, the present study provides a useful framework for understanding how appliance geometry influences mechanical loading and, consequently, for optimizing therapeutic design.

As we previously mentioned, the Ni-Ti archwire is assumed to be linearly elastic, although its actual behavior is more complex, so our model does not incorporate all material, contact, and biological effects. Thus, the importance of considering material-specific properties and their interaction with the surrounding biological environment has also been highlighted in recent biomedical engineering research. Based on investigations of polymer-coated magnesium-based biomaterials under simulated physiological conditions, a clear structure–property–performance relationship was demonstrated, showing that differences in material characteristics and interactions with the biological medium can substantially influence functional behavior and durability. Although the materials and clinical application investigated by Sun et al. differ from those considered in the present orthodontic model, their findings reinforce the broader principle that the performance of biomedical systems cannot be interpreted solely from their geometry, but should also account for material-specific and environmental factors. In the present study, the Ni-Ti orthodontic archwire was modeled using a defined elastic modulus and a simplified linear-elastic response. Consequently, incorporating more complex constitutive material behavior and clinically relevant environmental interactions into future models could provide an even more realistic representation of the force system developed within orthodontic appliances [41].

As we mentioned, based on the connection between the mechanical optimization achieved through orthodontics and the subsequent biological and biomechanical requirements of implant therapy, the clinical relevance of restoring an adequate implant-prosthetic space should also be considered in relation to the subsequent biological and biomechanical requirements of implant-supported rehabilitation. Recent advances in dental biomaterials emphasize that successful implant therapy depends not only on appropriate implant positioning but also on the interaction between implant material characteristics and the surrounding biological tissues. A study demonstrated that surface modification of titanium implants using Zn/Ca co-doped manganese phosphate coatings can improve material properties, including bonding strength and corrosion resistance, while simultaneously promoting osteogenic activity. Although the present study focuses on the orthodontic phase preceding implant placement rather than on implant surface modification, these findings emphasize the interdisciplinary nature of implant-prosthetic rehabilitation. From this perspective, accurate orthodontic control of tooth position, root orientation, and the dimensions of the future implant-prosthetic space provides the anatomical and biomechanical conditions required for subsequent implant placement, while advances in implant biomaterials may further contribute to favorable biological integration and long-term treatment stability [42].

Another aspect that should be considered in the further development of biomechanical models is the tribological behavior of dental materials and interacting components. Thus, in recent research, a friction-induced polytetrafluoroethylene (PTFE) coating for dental restorative composite resin was developed, and it demonstrated a substantial reduction in friction and wear, with the coated material maintaining a low coefficient of friction under prolonged testing. Although their investigation focused on restorative materials rather than orthodontic components, the findings emphasize the importance of surface characteristics and frictional interactions in determining the mechanical performance and durability of dental biomaterials. This observation is particularly relevant to the present study because archwire–bracket friction and nonlinear contact were not incorporated into the proposed mechanical model. Future extensions of the present methodology could therefore include experimentally determined friction coefficients, surface-dependent contact conditions, and more complex archwire–bracket interactions to provide a more comprehensive representation of the force system developed under clinical conditions [43].

Finally, the importance of integrating mechanical design with material-specific and biological considerations is also supported by recent developments in biomedical engineering. Researchers developed gelatin/heparin-coated bio-inspired polyurethane composite fibers for small-caliber artificial blood vessel grafts, combining tailored mechanical properties with surface functionalization intended to promote favorable biological responses. Although their application differs substantially from the orthodontic system investigated in the present study, their findings illustrate the broader principle that the performance of biomedical devices depends on the interaction between structural design, material characteristics, and the surrounding biological environment. From this perspective, future developments of the present orthodontic model could extend beyond geometric and elastic-force analysis by incorporating more realistic material behavior and biological boundary conditions, thereby contributing to increasingly comprehensive and patient-specific biomechanical simulations [44].

Through the complexity of the geometric model and the integration of classical engineering methods into a three-dimensional CAD environment, the study demonstrates the feasibility of a rigorous interdisciplinary approach capable of providing a solid foundation for further numerical or experimental developments. Compared to existing studies, the main contribution lies in the shift from a general description of orthodontic biomechanics to a customized analysis based on the patient’s actual geometry and direct virtual measurements.

The differences observed between the analytical and FEM results can also be attributed to the three-dimensional orientation of the brackets and archwire segments. In the analytical formulation, the local wire segment is represented by an idealized beam geometry, whereas the FEM model preserves the spatial orientation of the patient-specific dental structures. Consequently, the FEM solution accounts for force components arising from the actual three-dimensional configuration, while the analytical model primarily represents the transverse response of the selected wire segment.

Although good agreement was obtained for most configurations, larger discrepancies were observed for selected mandibular configurations. These differences indicate that the analytical beam approximation is sensitive to the local spatial geometry, bracket orientation, and contact conditions. Such cases represent the limits of applicability of the simplified analytical formulation and emphasize the importance of three-dimensional numerical analysis for configurations involving more pronounced geometric deviations from the idealized beam assumptions.

The frictional interaction between the NiTi archwire and the bracket was included in the FEM model using a coefficient of friction of 0.01. This interaction is not explicitly represented in the analytical beam formulation and may contribute to differences between the two approaches, particularly in configurations where the wire changes direction over successive brackets.

The periodontal ligament was represented using a homogeneous isotropic linear elastic constitutive model. This assumption was adopted to maintain numerical stability and computational feasibility; however, it does not reproduce the nonlinear, time-dependent, and potentially anisotropic behavior of the periodontal ligament. Consequently, the predicted reaction forces should be interpreted as mechanical estimates corresponding to the adopted constitutive assumptions rather than as exact representations of the in vivo periodontal response.

The NiTi archwire was represented using an equivalent elastic modulus. This approach does not reproduce the complete stress-induced phase transformation and superelastic plateau characteristic of NiTi alloys. Therefore, the FEM results should be interpreted within the elastic-equivalent material assumption adopted in the present study.

## 5. Conclusions

The present study developed a patient-specific three-dimensional geometric reconstruction of a dento-maxillary system and an associated orthodontic appliance based on CBCT data and reverse- and direct-engineering procedures.

The reconstructed geometry was used to determine the spatial positions of the bracket-related points and to extract the local geometric parameters required for the analytical evaluation of the orthodontic archwire. The subsequent mechanical calculation was based on a local equivalent Euler–Bernoulli beam formulation.

Within the assumptions of this simplified model, the results demonstrated a non-uniform distribution of equivalent local elastic force estimates and transverse reaction components along the maxillary and mandibular orthodontic archwires. The calculated values were strongly influenced by the local geometry of the archwire and by the relative positions of the bracket-related reference points.

The reconstructed anatomical components contributed to the analysis by defining the patient-specific geometric configuration of the orthodontic system. However, their constitutive behavior and deformation were not mechanically coupled to the analytical force calculation.

Therefore, the present methodology should be interpreted as a geometry-based reduced-order approach for estimating local elastic force components rather than as a complete three-dimensional force–moment analysis of the orthodontic appliance.

Future research should extend the present approach by incorporating continuous three-dimensional beam formulations or finite element models, experimentally determined bracket–archwire interactions, friction and contact effects, nonlinear material behavior, moment transmission, and quantitative mechanical validation against experimentally measured orthodontic forces.

## Figures and Tables

**Table 1:** Mathematical models applied to the jaw.

M_1dr_	C_dr_	I_2dr_	I_1dr_	I1st	I2st	C_st_	M1st		M2st
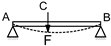									
	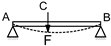								
		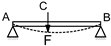							
			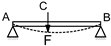						
				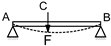					
					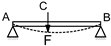				
						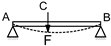			
	F = k·s	F = k·s	F = k·s	F = k·s	F = k·s	F = k·s	F = k·s		
V = V_A_	V = V_A_	V = V_A_ + V_B_	V = V_A_ + V_B_	V = V_A_ + V_B_	V = V_A_ + V_B_	V = V_A_ + V_B_	V = V_A_	V = V_A_	

**Table 2:** Mathematical models applied to the mandible.

M_2dr_	P_2dr_	P_1dr_	C_dr_	I_2dr_	I_1dr_	I1st	I2st	C_st_	P1st	P2st	M2st
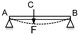											
	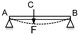										
		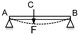									
			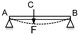								
				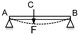							
					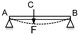						
						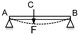					
							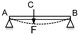				
								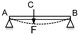			
									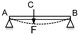		
	F = k·s	F = k·s	F = k·s	F = k·s	F = k·s	F = k·s	F = k·s	F = k·s	F = k·s	F = k·s	

**Table 3:** Mathematical models applied to the jaw.

M_1dr_	C_dr_	I_2dr_	I_1dr_	I1st	I2st	C_st_	M1st	M2st
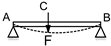								
	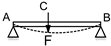							
		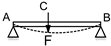						
			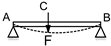					
				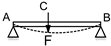				
					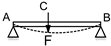			
						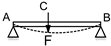		
	F = k·s	F = k·s	F = k·s	F = k·s	F = k·s	F = k·s	F = k·s	
V = V_A_	V = V_A_	V = V_A_ + V_B_	V = V_A_ + V_B_	V = V_A_ + V_B_	V = V_A_ + V_B_	V = V_A_ + V_B_	V = V_A_	V = V_A_

**Table 4:** Mathematical models applied to the mandible.

M_2dr_	P_2dr_	P_1dr_	C_dr_	I_2dr_	I_1dr_	I1st	I2st	C_st_	P1st	P2st	M2st
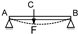											
	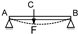										
		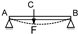									
			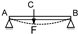								
				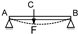							
					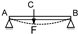						
						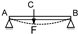					
							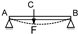				
								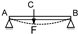			
									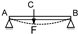		
	F = k·s	F = k·s	F = k·s	F = k·s	F = k·s	F = k·s	F = k·s	F = k·s	F = k·s	F = k·s	

**Table 5:** Physical and mechanical properties of the materials used in Ansys.

Component	Material	Density [Kg/m^3^]	Young’s Module [Pa]	Poisson’s Coefficient
Jaw Mandible	Bone	1.400	1 × 10^10^	0.31
Tooth	Enamel	2.958	7.79 × 10^10^	0.3
Periodontal ligament	Ligament	1.100	6.8 × 10^6^	0.45
Jaw Mandible	Bone	1.400	1 × 10^10^	0.31
Orthodontic archwire	Nitinol	6.450	8.3 × 107	0.33
Bracket-type elements	Ni + Cr Alloy	8.500	2.1 × 10^11^	0.31
Adhesive	Transbond XT	1.950	5 × 10^9^	0.3

**Table 6:** The distances L, a, and s measured along the curve of the orthodontic archwire.

	M_1dr_	C_dr_	I_2dr_	I_1dr_	I1st	I2st	C_st_	M1st	M2st
**L [mm]**		29.75	16.23	23.17	22.34	16.61	31.8	34.93	
**a [mm]**	22.94	7.36	8.87	14.3	8.04	8.57	23.23	11.7	
**s [mm]**		1.41	2.31	2.09	2.22	1.88	1.65	2.29	

**Table 7:** The distances L, a, and s measured along the curve of the orthodontic archwire–mandible.

	M_2dr_	P_2dr_	P_1dr_	C_dr_	I_2dr_	I_1dr_	I1st	I2st	C_st_	P1st	P2st	M2st
**L [mm]**		29.8	16.21	16.45	15.21	15.1	14.02	14.53	16.34	16.9	28.38	
**a [mm]**	22.96	6.93	9.28	7.17	8.04	7.1	6.92	7.61	8.73	8.17	20.21	
**s [mm]**		1.41	0.6	1.41	1.54	1.21	1.01	1.31	1.05	0.97	1.5	

**Table 8:** Equivalent local elastic forces and transverse reaction components calculated for the maxillary orthodontic archwire.

	M_1dr_	C_dr_	I_2dr_	I_1dr_	I1st	I2st	C_st_	M1st	M2st
**F [N]**		0.0612	0.3833	0.12681	0.15626	0.28816	0.04856	0.04419	
**V [N]**	0.04725	0.1738	0.0625	0.30955	0.21775	0.06932	0.17807	0.02938	0.014

**Table 9:** Equivalent local elastic forces and transverse reaction components calculated for the mandibular orthodontic archwire.

	M_2dr_	P_2dr_	P_1dr_	C_dr_	I_2dr_	I_1dr_	I1st	I2st	C_st_	P1st	P2st	M2st
**F [N]**		0.0596	0.1016	0.2272	0.3083	0.24	0.2571	0.3004	0.1698	0.14	0.05	
**V [N]**	0.04	0.0434	0.1420	0.2035	0.2295	0.29	0.2583	0.2700	0.3147	0.18	0.06	0.04

**Table 10:** Reaction values obtained from the FEM simulation for the teeth in the maxilla.

Teeth/Values for Reaction Force, Error	First Right Molar	Right Canine	Right Lateral Incisor	Right Central Incisor	Left Central Incisor	Left Lateral Incisor	Left Canine	Left First Molar	Left Second Molar
**V_A,analytical_**	0.047258093	0.173862005	0.048548243	0.100024353	0.139486	0.013089	0.029389	0.029389	0.014802
**V_B,analytical_**	0	0	0.014029103	0.209532063	0.078268	0.056237	0.148681	0	0
**V_analytical_**	0.047258093	0.173862005	0.062577345	0.309556416	0.217755	0.069326	0.178071	0.029389	0.014802
**V_A,FEM_**	0.045522	0.17723913	0.046496923	0.100209524	0.140379	0.013779	0.02912	0.03126	0.014229
**V_B,FEM_**	0	0	0.012025	0.196659	0.08073	0.055478	0.139181	0	0
**V_FEM_**	0.045522	0.17723913	0.058521923	0.296868524	0.221109	0.069256	0.168301	0.03126	0.014229
**Error [%]**	3.813744827	1.905406205	6.92974855	4.273909439	1.516927	0.10108	5.80475	5.984131	4.027853

**Table 11:** Reaction values obtained from the FEM simulation for the teeth on the mandible.

Teeth/Values for Reaction Force, Error	Right Second Molar	Right Second Premolar	Right First Premolar	Right Canine	Right Lateral Incisor	Right Central Incisor	Left Central Incisor	Left Lateral Incisor	Left Canine	Left First Premolar	Left Second Premolar	Left Second Molar
**V_A,analytical_**	0.0458	0.0434	0.1281	0.1453	0.1305	0.1302	0.1430	0.1430	0.1573	0.0907	0.0682	0.0421
**V_B,analytical_**	0	0	0.0138	0.0581	0.0990	0.1629	0.1152	0.1269	0.1573	0.0907	0	0
**V_analytical_**	0.0458	0.0434	0.1420	0.2035	0.2295	0.2932	0.2583	0.2700	0.3147	0.1815	0.0682	0.0421
**V_A,FEM_**	0.0410	0.0459	0.1403	0.1409	0.1358	0.1311	0.1394	0.1413	0.1571	0.0892	0.0694	0.0418
**V_B,FEM_**	0	0	0.01325	0.0563	0.0106	0.1580	0.1384	0.1289	0.1546	0.0917	0	0
**V_FEM_**	0.0410	0.0459	0.1535	0.1973	0.1465	0.2891	0.2778	0.2702	0.3117	0.181016	0.069446	0.041843
**Error [%]**	11.725	5.4496	7.4869	3.1052	56.694	1.3885	7.00827	0.075578	0.9459	0.28175	1.72446	0.62448

## Data Availability

The original contributions presented in this study are included in the article. Further inquiries can be directed to the corresponding authors.
